# Origin and Evolution of Flavin-Based Electron Bifurcating Enzymes

**DOI:** 10.3389/fmicb.2018.01762

**Published:** 2018-08-03

**Authors:** Saroj Poudel, Eric C. Dunham, Melody R. Lindsay, Maximiliano J. Amenabar, Elizabeth M. Fones, Daniel R. Colman, Eric S. Boyd

**Affiliations:** Department of Microbiology and Immunology, Montana State University, Bozeman, MT, United States

**Keywords:** electron bifurcation, flavin, ferredoxin, anoxic, subsurface, oxidoreductase, LUCA, metagenomes

## Abstract

Twelve evolutionarily unrelated oxidoreductases form enzyme complexes that catalyze the simultaneous coupling of exergonic and endergonic oxidation–reduction reactions to circumvent thermodynamic barriers and minimize free energy loss in a process known as flavin-based electron bifurcation. Common to these 12 bifurcating (Bf) enzymes are protein-bound flavin, the proposed site of bifurcation, and the electron carrier ferredoxin. Despite the documented role of Bf enzymes in balancing the redox state of intracellular electron carriers and in improving the efficiency of cellular metabolism, a comprehensive description of the diversity and evolutionary history of Bf enzymes is lacking. Here, we report the taxonomic distribution, functional diversity, and evolutionary history of Bf enzyme homologs in 4,588 archaeal, bacterial, and eukaryal genomes and 3,136 community metagenomes. Bf homologs were primarily detected in the genomes of anaerobes, including those of sulfate-reducers, acetogens, fermenters, and methanogens. Phylogenetic analyses of Bf enzyme catalytic subunits (oxidoreductases) suggest they were not a property of the Last Universal Common Ancestor of Archaea and Bacteria, which is consistent with the limited and unique taxonomic distributions of enzyme homologs among genomes. Further, phylogenetic analyses of oxidoreductase subunits reveal that non-Bf homologs predate Bf homologs. These observations indicate that multiple independent recruitments of flavoproteins to existing oxidoreductases enabled coupling of numerous new electron Bf reactions. Consistent with the role of these enzymes in the energy metabolism of anaerobes, homologs of Bf enzymes were enriched in metagenomes from subsurface environments relative to those from surface environments. Phylogenetic analyses of homologs from metagenomes reveal that the earliest evolving homologs of most Bf enzymes are from subsurface environments, including fluids from subsurface rock fractures and hydrothermal systems. Collectively, these data suggest strong selective pressures drove the emergence of Bf enzyme complexes via recruitment of flavoproteins that allowed for an increase in the efficiency of cellular metabolism and improvement in energy capture in anaerobes inhabiting a variety of subsurface anoxic habitats where the energy yield of oxidation-reduction reactions is generally low.

## Introduction

**FIGURE 1:**
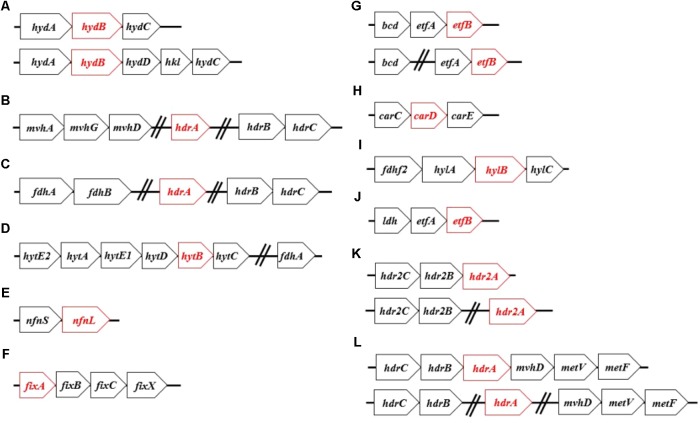
The most commonly identified operon structures for characterized Bf enzyme homologs: **(A)** Hyd, **(B)** Mvh, **(C)** Fdh, **(D)** Hyt, **(E)** Nfn, **(F)** Fix, **(G)** Bf-Bcd, **(H)** Car, **(I)** Hyl, **(J)** Bf-Ldh, **(K)** Hdr2, and **(L)** Met. Names of each abbreviated complex are presented in **Table 1**. Open reading frames that are highlighted in red represent genes encoding the proposed Bf flavoprotein subunit of the specified complex. The genes following the hashed lines indicate that they are not necessarily co-localized and can be found remotely in the genome.

**FIGURE 2:**
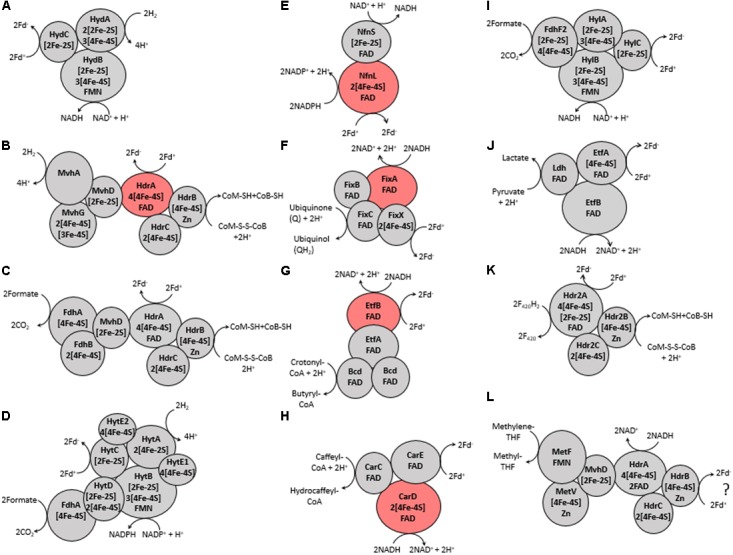
Proposed protein-protein interactions and the number and type of cofactors present in each protein subunit, as defined previously in references characterizing each Bf enzyme (see **Table 1**). **(A)** Hyd, **(B)** Mvh, **(C)** Fdh, **(D)** Hyt, **(E)** Nfn, **(F)** Fix, **(G)** Bf-Bcd, **(H)** Car, **(I)** Hyl, **(J)** Bf-Ldh, **(K)** Hdr2, and **(L)** Met. The ‘?’ in **(L)** indicates that the third substrate is not known for this enzyme but is predicted to be ferredoxin (Fd) (Mock et al., 2014). Names of each abbreviated complex are presented in **Table 1**. The reversibility of the enzyme complexes is not shown to better depict coupling of substrate oxidation/reduction. Subunits that are highlighted red are flavoprotein subunits that have been biochemically shown to be the site of electron bifurcation.

Flavin-based electron bifurcation (FBEB) involves the simultaneous reduction of two electron acceptors using a single electron donor in an enzyme complex, whereby a thermodynamically favorable exergonic reaction drives a thermodynamically unfavorable endergonic reaction (Buckel and Thauer, 2013; Peters et al., 2016). A total of 12 enzymes have been shown to catalyze FBEB to date (**Table 1**) and each of these comprise multiple protein subunits that form a complex (**Figures 1**, **2**). A common theme among bifurcating (Bf) enzymes is the involvement of ferredoxin (Fd) as a substrate as well as coordination of at least one flavin, the proposed site of bifurcation (Buckel and Thauer, 2013; Peters et al., 2016). Studies conducted on NAD(H)-dependent reduced Fd:NADP(H) oxidoreductase (Nfn), a protein complex that catalyzes the simultaneous endergonic reduction of oxidized Fd (Fd^+^) and exergonic reduction of NAD^+^ via oxidation of NADPH (Wang et al., 2010), reveals that the fully reduced flavin (hydroquinone) undergoes one electron oxidation by NAD^+^ to generate an unstable flavin anionic semiquinone that is then further oxidized by one electron by Fd^+^ (Lubner et al., 2017). Thus, an exergonic electron transfer from the flavin hydroquinone to NAD^+^ ‘pays for’ the endergonic reduction of Fd^+^ via the unstable semiquinone intermediate. In this way, Bf Nfn functions to reversibly reduce Fd^+^ that can then be used to drive low potential electron transfer reactions. Nfn also functions to balance the ratio of oxidized to reduced NAD(H) and NADP(H) and is thus a key regulator modulating the favorability of catabolic and anabolic reactions (Wang et al., 2010; Demmer et al., 2015; Lubner et al., 2017).

**Table 1:** The 12 enzyme complexes that have been biochemically shown to bifurcate electrons to date.

**Enzyme**	**Subunits^a^**	**Protein ID**	**E-value**	**Flanking region**	**Reference**
(A) NAD(H)-dependent [FeFe]-hydrogenase (Hyd)	**HydA**	AAD36496	2e-20		Schut and Adams, 2009


	HydB	AAD36495	2e-13	±5	Schut and Adams, 2009
	HydC	AAD36494	1e-07	±5	Schut and Adams, 2009

(B) [NiFe]-hydrogenase/heterodisulfide reductase (Mvh)	**MvhA**	CAF30379	1e-90		Thauer et al., 2008; Kaster et al., 2011


	MvhG	CAF30378	5e-07	±3	Thauer et al., 2008; Kaster et al., 2011
	MvhD	CAF30377	4e-07	±3	Thauer et al., 2008; Kaster et al., 2011
	HdrA	CAF30381	3e-08	All**^b^**	Thauer et al., 2008; Kaster et al., 2011
	HdrB	CAF30711	1e-03	All**^b^**	Thauer et al., 2008; Kaster et al., 2011
	HdrC	CAF30710	2e-06	All**^b^**	Thauer et al., 2008; Kaster et al., 2011

(C) Formate dehydrogenase/heterodisulfide reductase (Fdh)	**FdhA**	CAF30854	6e-12		Costa et al., 2010, 2013


	FdhB	CAF30853	8e-15	±1	Costa et al., 2010, 2013
	HdrA	CAF30381	3e-08	All**^b^**	Costa et al., 2010, 2013
	HdrB	CAF30711	1e-03	All**^b^**	Costa et al., 2010, 2013
	HdrC	CAF30710	2e-06	All**^b^**	Costa et al., 2010, 2013

(D) NADP(H)-dependent [FeFe]-hydrogenase (Hyt)	FdhA	AGT29705	6e-12	±10	Wang et al., 2013a


	HytE2	AGT29714	1e-43	±10	Wang et al., 2013a
	**HytA**	AGT29713	1e-04		Wang et al., 2013a
	HytE1	AGT29712	1e-43	±10	Wang et al., 2013a
	HytD	AGT29711	3e-17	±10	Wang et al., 2013a
	HytB	AGT29710	2e-13	±10	Wang et al., 2013a
	HytC	AGT29709	1e-07	±10	Wang et al., 2013a

(E) NAD(H)-dependent reduced ferredoxin:NADP(H) oxidoreductase (Nfn)	NfnS	AAD36706	9e-22		Demmer et al., 2015


	**NfnL**	AAD36707	2e-96	±1	Demmer et al., 2015

(F) Electron transfer flavoprotein involved in nitrogen fixation (Fix)	**FixA/EtfB**	WP_011665895	1e-43		Edgren and Nordlund, 2004


	FixB/EtfA	WP_011665894	6e-45	±4	Edgren and Nordlund, 2004
	FixC	WP_011665893	4e-35	±4	Edgren and Nordlund, 2004
	FixX	WP_011665892	3e-05	±4	Edgren and Nordlund, 2004

(G) Butyryl-CoA dehydrogenase/electron transfer flavoprotein (Bf-Bcd)	**Bcd**	EDK32509	3e-12		Herrmann et al., 2008; Li et al., 2008


	EtfA	EDK32511	6e-45	±2	Herrmann et al., 2008; Li et al., 2008
	EtfB	EDK32510	1e-43	±2	Herrmann et al., 2008; Li et al., 2008

(H) Caffeyl-CoA reductase/electron transfer flavoprotein (Car)	**CarC**	AFA48354	2e-12		Bertsch et al., 2013


	CarD/EtfB	AFA48355	1e-43	±2	Bertsch et al., 2013
	CarE/EtfA	AFA48356	6e-45	±2	Bertsch et al., 2013

(I) NAD(H)-dependent formate dehydrogenase (Hyl)	FdhF2	AFS79904	6e-12	±4	Wang et al., 2013b


	**HylA**	AFS79905	2e-39		Wang et al., 2013b
	HylB	AFS79906	3e-14	±4	Wang et al., 2013b
	HylC	AFS79907	5e-38	±4	Wang et al., 2013b

(J) Lactate dehydrogenase/electron transfer flavoprotein (Bf-Ldh)	**Ldh**	AFA47664	3e-29		Weghoff et al., 2015


	EtfA	AFA47663	6e-45	±2	Weghoff et al., 2015
	EtfB	AFA47662	1e-43	±2	Weghoff et al., 2015

(K) F_420_H_2_-dependent heterodisulfide reductase (Hdr2)	**Hdr2A**	AAM06247	3e-08	All**^c^**	Yan et al., 2017


	Hdr2B	AAM07582	1e-03	±2 HdrC**^c^**	Yan et al., 2017
	Hdr2C	AAM07581	2e-06	±2 HdrB**^c^**	Yan et al., 2017

(L) Methylene-tetrahydrofolate (H_4_F) reductase/heterodisulfide reductase (Met)	**MetF**	YP_430048	9e-13		Mock et al., 2014


	MetV	YP_430049	0.1	±3	Mock et al., 2014
	MvhD	YP_430050	4e-07	±3	Mock et al., 2014
	HdrA	YP_430051	3e-08	All	Mock et al., 2014
	HdrB	YP_430052	1e-03	All	Mock et al., 2014
	HdrC	YP_430053	2e-06	All	Mock et al., 2014

*^a^Subunits in bold are the primary oxidoreductase catalytic subunits that were used as bait sequences when identifying Bf enzyme homologs.*

*^b^Co-localization of hdrABC with mvh, fdh, or met is not necessary for Bf function in these enzyme complexes (Kaster et al., 2011; Costa et al., 2013; Bertsch et al., 2015). Thus, genomes containing these subunits in any location were considered as having a Bf homolog. ^c^Hdr2A is often not co-localized with Hdr2BC [i.e., as in Methanosarcina acetivorans where Hdr2ABC was first described (Yan et al., 2017)]. Thus, Hdr2A was allowed to be located anywhere in the genome, while Hdr2B and Hdr2C were required to be co-localized for inclusion as a Bf Hdr2 complex. The minimum complement of subunits comprising each enzyme, a reference NCBI protein sequence ID, an oxE-value cutoff for demarcating subunit homologs from closely related paralogs, the number of open reading frames from the catalytic subunit (boldfaced) searched for homologs of companion subunits, and literature references are provided.)*

In addition to allowing for the reduction of the low potential electron carrier Fd^+^ and balancing the redox state of the pyridine nucleotide pool, FBEB has been suggested to improve the efficiency of cellular metabolism by allowing for more complete capture of energy released during substrate oxidation (Herrmann et al., 2008; Buckel and Thauer, 2013). This phenomenon appears to be particularly relevant for anaerobes that inhabit highly reduced environments where metabolic intermediates often accumulate due to difficulty in regenerating endogenous oxidants (i.e., Fd^+^ or NAD^+^) for those compounds. For example, glucose fermentation in *Clostridium pasteurianum* generates 3.0 mol ATP per glucose when non-Bf butyryl-CoA dehydrogenase (Bcd) is involved whereas 3.3 mol ATP per glucose is generated when Bf Bcd is involved, an increase of ∼11% (Jungermann et al., 1973; Herrmann et al., 2008; Buckel and Thauer, 2013). During oxidation of 1.5 mol of glucose in the Embden–Meyerhof–Parnas pathway, a total of three NADH and three reduced Fd (Fd^-^) are generated. All three Fd^-^ are shuttled toward hydrogen (H_2_) generation, while all three NADH are used to produce butyryl-CoA when non-Bf Bcd is involved. However, when Bf Bcd is involved, one NADH is used to reduce Fd^+^ that eventually yields an additional H_2_, one NADH is used to reduce two acetyl-CoA to crotonyl-CoA, and the other NADH is used to further reduce crotonyl-CoA to butyryl-CoA, which generates additional ATP. The reader is referred to several recent reviews (Herrmann et al., 2008; Buckel and Thauer, 2013, 2018b; Peters et al., 2016, 2018; Müller et al., 2018) for additional discussion and examples of enhanced energy capture in the presence of Bf systems.

Despite the multiple, yet interrelated, roles of FBEB in the energy metabolism of anaerobes, little is known of the taxonomic distribution and functional diversity of Bf enzymes among cultivars or in the natural environment. Moreover, despite suggestions that FBEB was integrated into the metabolism of primitive lifeforms on early Earth (Martin, 2012; Nitschke and Russell, 2012; Martin et al., 2017; Baymann et al., 2018; Sousa et al., 2018) little is known of the evolutionary history of these enzymes or of the characteristics of environments that might have led to their evolutionary origin(s). In the present study, we hypothesized that homologs of Bf enzymes would be enriched in the genomes of anaerobes relative to aerobes and in communities that inhabit subsurface environments that tend to be reduced relative to those that inhabit surface environments that tend to be oxidized (Colman et al., 2017). Moreover, we hypothesized that Bf enzymes first evolved in anaerobes that inhabit subsurface environments. Finally, given that Bf enzymes are multi-subunit complexes (as summarized in Buckel and Thauer, 2013; Peters et al., 2016) and often exhibit limited taxonomic distributions (Poudel et al., 2016; Berry et al., 2017; Garcia Costas et al., 2017; Nguyen et al., 2017; Buckel and Thauer, 2018b), we hypothesized that Bf enzyme complexes emerged after the divergence of Archaea and Bacteria from the Last Universal Common Ancestor (LUCA). To address these interrelated hypotheses, we used bioinformatics techniques informed by biochemical data to identify homologs of the 12 known and biochemically characterized Bf enzyme complexes in 4,588 complete genome sequences. The distribution, diversity and evolutionary history of Bf enzyme homologs were analyzed in the context of the physiology of host organisms based on prior characterizations. Phylogenetic analyses were conducted on each of the 12 Bf enzyme homolog datasets to identify microbial lineages that host the earliest evolving enzyme systems. To better define the characteristics of environments that select for microbial cells with electron Bf capability, and that may have precipitated their emergence, we also characterized the distribution and diversity of homologs of the 12 biochemically characterized Bf enzyme complexes in 3,136 available community metagenomes from a range of environments and cross-referenced this information with available metadata for these environments. Phylogenetic reconstructions were then performed to identify characteristics of environments that host early evolving homologs of Bf enzymes. Results are discussed in the context of the physiological and geochemical settings that enabled the multiple independent and recent origins of FBEB enzymes in biological systems and the role of environmental variation in driving the diversification of these enzymes.

## Materials and Methods

### Generation of a Genomic and Metagenomic Sequence Database

All complete genomes (*n* = 4,588) available in the National Center for Biotechnology Information (NCBI) database as of March 2016 were compiled. This compiled database included complete genomes of Archaea (*n* = 230), Bacteria (*n* = 4,343), and Eukarya (*n* = 15). In addition, the protein sequences encoded in each of the environmental metagenomes (*n* = 3,136) in the Department of Energy’s Integrated Microbial Genomes and Microbiomes (DOE-IMG) (Markowitz et al., 2011) as of April, 2017 were compiled. The DOE-IMG database was selected since it includes standardized metadata for metagenomes that are not always included in submissions to other databases such as NCBI or MG-RAST.

### Identification and Compilation of Electron Bf Enzyme Homologs in Genomes and Metagenomic Datasets

#### Overview of Approach

The minimum number of subunits that comprise a Bf enzyme complex (**Table 1**) was identified based on literature surveys and empirical bioinformatics analyses, as described below for each Bf system. Representative sequences of each subunit were used as bait sequences to extract all the homologs of each subunit for each enzyme complex from the complete genome database (described above) using the phmmer program via the HMMER (ver. 3) software package (Eddy, 2015). Compiled homologs of each subunit for each enzyme complex were aligned with Clustal Omega (Sievers et al., 2011) and were further filtered to remove homologs that did not exhibit conservation in key active site motifs if they have been defined, as described for each enzyme system below. As a further screen, we then examined the protein encoding genes that flank the gene coding for the catalytic subunit for each homolog for the presence of the minimum subunits required to constitute a Bf complex and removed those candidate Bf homologs that did not meet our specified criteria. These steps are similar to the approaches that were taken to demarcate homologs of Bf [FeFe]-hydrogenase (Poudel et al., 2016), Bf transhydrogenase (Berry et al., 2017; Nguyen et al., 2017), and Bf electron transfer flavoproteins (Garcia Costas et al., 2017) from non-Bf paralogs. Using this information, a specific E-value was empirically determined to demarcate Bf homologs from their closest paralogs in metagenomic datasets (**Table 1**). Importantly, since assembled genomes from natural environments are often incomplete (only partial genomes or contigs are available), and since our criteria required the presence of genes encoding the minimum complement of proteins that constitute a given Bf enzyme complex to be present for it to be counted as a homolog, the distribution of Bf enzyme homologs reported here for metagenomic sequences is likely to be a conservative estimate of their actual abundance.

For each Bf enzyme homolog, companion subunits for the catalytic subunit were identified in flanking gene regions based on the expected gene distributions from empirical analyses (see below for descriptions for each Bf enzyme as well as **Table 1**). A custom python script was used to assess the presence of companion subunits in each case, and the number of open reading frames that were surveyed for companion subunits are provided for each catalytic subunit in **Table 1**. If the genes encoding subunits necessary for bifurcation capacity were identified within these open reading frames, the enzyme homolog was considered as a putative Bf enzyme, whereas putative non-Bf enzyme homologs were identified by the absence of the specified subunits in the flanking gene regions. We also screened metagenomes for homologs of genes coding for putative Bf enzymes using the same approaches outlined briefly above and as described for each system below in more detail.

#### [FeFe]-Hydrogenase (Hyd)

Our previous bioinformatics work classified putative Bf [FeFe]-hydrogenases (Hyd) as multimeric (**Figure 1A**) and non-Bf [FeFe]-hydrogenases as monomeric or dimeric (Poudel et al., 2016). Putatively Bf trimeric Hyd complexes, first identified in *Thermotoga maritima*, include the catalytic subunit HydA, HydB that contains a flavin binding site, and HydC that contains ligands for iron-sulfur (FeS) cluster(s) (**Figure 2A**) (Schut and Adams, 2009; Schuchmann and Muller, 2012). In addition to HydABC, tetrameric Hyd complexes comprise HydD that includes numerous conserved cysteine residues that are putatively involved in coordinating FeS cluster(s) (Poudel et al., 2016). Therefore, the minimum gene complement that encodes for a putative Bf Hyd is *hydABC* (minimum among both trimeric and tetrameric forms) and these genes tend to be co-localized in genomes (Poudel et al., 2016). Because HydA often includes N- and C-terminal domains with homology to various proteins and FeS cluster binding domains such as is the case for HydA from *T. maritima* (AAD36496, **Table 1**), we first screened our databases using HydA from *Chlamydomonas reinhardtii* (AAL23572) as a query since it does not encode N- and C-terminal FeS cluster binding motifs (Mulder et al., 2010). Extracted HydA homologs (Bf and non-Bf) were then aligned with Clustal Omega (Sievers et al., 2011) and were further filtered to remove homologs that did not exhibit conservation in key active site L1, L2, and L3 motifs, as we and others have previously described (Meyer, 2007; Poudel et al., 2016).

#### [NiFe]-Hydrogenase (Mvh)

Previous bioinformatics analyses have classified [NiFe]-hydrogenase into four phylogenetically and functionally coherent groups, with the “c” subgroup of group 3 (group 3c or Mvh) harboring homologs of Bf enzymes (Vignais et al., 2001; Boyd et al., 2014; Greening et al., 2016). Mvh comprise MvhA (the catalytic subunit where H_2_ oxidation occurs), MvhG, and MvhD; both MvhG and MvhD harbor FeS cluster binding motifs (Peters et al., 2015; Greening et al., 2016). Mvh associates with heterodisulfide reductase (Hdr) forming a hexameric complex. This complex includes HdrA where the proposed Bf flavin is coordinated, and HdrB and HdrC, both of which have FeS cluster binding motifs (**Figures 1B**, **2B**) (Kaster et al., 2011; Buckel and Thauer, 2013). Therefore, the minimum number of subunits required for a Bf Mvh includes MvhADG and HdrABC. All homologs of the large subunit of [NiFe]-hydrogenase were compiled (see **Table 1**), aligned and screened for distal and vicinal cysteine pairs that delineate [NiFe]-hydrogenase from paralogs such as MbxL, FuoD, and NuoD (Schut et al., 2013). MvhA harbors L1 (ICGxCxxxH) and L2 (AYDPCccCATH) sequence motifs that delineate it from other non-Bf [NiFe]-hydrogenase large subunit groups (i.e., groups 1, 2, 3a, 3b, 3d, and 4) (Vignais et al., 2001; Greening et al., 2016). Therefore, the extracted MvhA homologs were further filtered to only include sequences that contained those motifs.

The HdrABC proteins that form a complex with MvhAGD are often not co-localized with MvhAGD [as is the case for *Methanothermobacter marburgensis* where Mvh-Hdr was first described (Kaster et al., 2011)]. Therefore, we relaxed our script to allow for genes that encode for HdrABC to be located anywhere in genomes that also encode MvhAGD. However, this approach cannot be used to scan for the subunits of HdrABC in metagenomes since the possibility exists that these genes could be from a different genome than homologs of MvhAGD. To identify the Mvh homologs in metagenomes we only screened for MvhAGD subunits, since, to date they have only been shown to associate with HdrABC and are co-localized (Kaster et al., 2011; Greening et al., 2016). Thus, if MvhAGD was identified, we assumed that HdrABC were also likely to be encoded in the same genome in our metagenomic screens.

#### Heterodisulfide-Linked Formate Dehydrogenase (Fdh)

Formate dehydrogenase typically functions as a monomeric unit (Ferry, 1990), but can associate with Hdr (i.e., HdrABC) to form a Bf Fdh complex. This enzyme complex was first identified in *Methanococcus maripaludis* (**Figure 1C**; Costa et al., 2010, 2013). In fact, formate dehydrogenase was shown to associate with the same protein domain in MvhD that MvhAG associates with during the Mvh-Hdr bifurcation reaction (Costa et al., 2013). Hence, the MvhAG complex has been suggested to compete with formate dehydrogenase for MvhD (Costa et al., 2010, 2013). The Bf Fdh complex comprises two subunits, FdhA and FdhB, in addition to HdrABC subunits. FdhA exhibits a unique cysteine signature (i.e., CxxCxxCx_26_C ) that distinguishes it from its paralog FdhF2; FdhF2 is involved in another Bf complex that is described below (Wang et al., 2013b). Therefore, FdhA sequences were aligned, and only sequences that contained the specified cysteine motifs were retained for downstream analysis. A similar approach to that described above for Mvh was taken to identify homologs of Fdh in metagenomes. Since genes encoding for HdrABC are often not co-localized with those coding for FdhAB, we assumed that if FdhAB were detected that HdrABC were also likely to be encoded in that same genome in our metagenomic screens.

#### NADP(H)-Dependent Formate Dehydrogenase (Hyt)

The NADP(H)-dependent formate dehydrogenase (Hyt) complex, first identified in *Clostridium autoethanogenum*, comprises seven subunits (**Figure 1D**) that include FdhA that contains a conserved [4Fe-4S] binding motif and HytA that is the catalytic site for H_2_ oxidation (Wang et al., 2013a). In addition, the Hyt complex includes HytB, which is thought to coordinate a flavin, as well as HytC, HytD, HytE1, and HytE2, all of which comprise FeS cluster binding motifs (**Figure 2D**). HytA is homologous to HydA and contains key active site motifs that are largely the same as those in HydA, including the L1 (TSCCPxW), L2 (MPCxxKxxE), and L3 (ExMACxxGCxxGGGxP) motifs. Likewise, HytB and HytC are homologous to HydB and HydC, respectively. Homologs of HytA were aligned and were screened for the L1, L2, and L3 motifs, as defined above; those sequences that did not contain these motifs were discarded. HytE1, HytE2, and HytABCD are co-localized in the genome (Supplementary Table S2), whereas FdhA tends to be located within four genes upstream of HylA (data not shown). These criteria were used to identify Bf Hyt in genomes and metagenomes (**Table 1**).

#### NAD(H)-Dependent Reduced Ferredoxin:NADP Oxidoreductase (Nfn)

Nfn, which was first identified in *Clostridium kluyveri* comprises two subunits: the small subunit (NfnS) that contains one FAD and one [2Fe-2S] cluster and the large subunit (NfnL) that contains one FAD and two [4Fe-4S] clusters (**Figures 1E**, **2E**) (Wang et al., 2010; Demmer et al., 2015; Berry et al., 2017; Lubner et al., 2017; Nguyen et al., 2017). Several classes of Nfn (NfnI, NfnII or Xfn and NfnIII) have been identified (Nguyen et al., 2017). NfnI has been shown to bifurcate electrons (Wang et al., 2010; Demmer et al., 2015; Berry et al., 2017; Lubner et al., 2017; Nguyen et al., 2017). NfnII retains a structure and cofactor composition like NfnI and thus has been proposed to bifurcate, although the high potential electron acceptor is likely to be different than NAD^+^ (Nguyen et al., 2017). While there exists no structural studies on NfnIII, multiple sequence comparison of NfnIII with NfnI and NfnII revealed conserved motifs that potentially ligate the bifurcating flavin suggesting that NfnIII may also bifurcate (Nguyen et al., 2017). For these reasons, we treated all identified Nfn homologs as Bf. Extracted NfnS and NfnL sequences were aligned individually and then screened for the presence of conserved motifs, as identified previously (Demmer et al., 2015), in order to further delineate putative Bf NfnSL homologs.

#### Electron Transfer Flavoprotein Involved in Nitrogen Fixation (Fix)

There are several classes of electron transfer flavoproteins, including those that bifurcate and those that do not, as discussed previously (Garcia Costas et al., 2017). Non-Bf Etf comprise two subunits, EtfA and EtfB, while all known Bf Etf that are involved in nitrogen fixation (i.e., Fix), comprise four subunits: FixA (EtfB), FixB (EtfA), FixC, and FixX (**Figure 1F**) (Edgren and Nordlund, 2004; Garcia Costas et al., 2017; Ledbetter et al., 2017). Fix from *Azotobacter vinelandii* was the first enzyme shown to bifurcate electrons (Ledbetter et al., 2017). FixA, FixB, and FixC all contain flavin binding domains, but FixA is the proposed site of bifurcation (Ledbetter et al., 2017). FixX contains two [4Fe-4S] cluster binding motifs (**Figure 2F**).

#### Butyryl-CoA Dehydrogenase/Electron Transfer Flavoprotein (Bf-Bcd)

Butyryl-CoA dehydrogenase (Bcd) is typically involved in fatty-acid metabolism (Djordjevic et al., 1995) and has not been shown to bifurcate electrons. However, Bcd is homotetrameric and can associate with EtfAB and form a Bf complex, as first identified in *C. kluyveri* (termed Bf-Bcd; Herrmann et al., 2008). A crystal structure of Bf-Bcd revealed that it indeed forms a complex with EtfA and EftB (Li et al., 2008; Demmer et al., 2017). All three subunits of this complex contain flavin binding motifs, while the flavin in EtfB has been shown to be the Bf site (**Figures 1G**, **2G**).

#### Caffeyl-CoA Reductase/Electron Transfer Flavoprotein (Car)

To our knowledge, caffeyl-CoA reductase (CarC) has not yet been reported to function on its own (see caveat below). Rather, characterized enzymes form a complex that consists of three subunits: CarC, EtfA (CarD), and EtfB (CarE). This complex was first identified in *Acetobacterium woodii* (Bertsch et al., 2013). All three subunits contain flavin binding domains and CarE potentially harbors the Bf flavin (**Figures 1H**, **2H**). In addition to containing a flavin binding domain, CarD also contains two [4Fe-4S] cluster binding motifs (**Figure 2H**).

#### NAD(H)-Dependent Formate Dehydrogenase (Hyl)

The NAD(H)-dependent formate dehydrogenase (Hyl) complex is tetrameric and was first identified in *Clostridium acidurici* (**Figure 1I**) (Wang et al., 2013b). The tetrameric complex includes HylA, HylB, HylC, and FdhF2 (i.e., a formate dehydrogenase). All of the subunits contain FeS cluster binding motifs (**Figure 2I**). In addition to putatively binding FeS clusters, HylB also contains a flavin binding motif (**Figure 2I**). HylA is homologous to HydA but lacks conservation in the three aforementioned active site motifs, L1, L2, and L3 (Wang et al., 2013b). Extracted HylA homologs were aligned and demarcated from HydA and HytA homologs by screening for the absence of conserved L1, L2, and L3 signature motifs.

#### Lactate Dehydrogenase/Electron Transfer Flavoprotein (Bf-Ldh)

Lactate dehydrogenase (Ldh) is involved in the reduction of pyruvate to lactate (Garvie, 1980). Ldh can associate with EtfAB and form a trimeric Bf complex (termed Bf-Ldh; **Figure 1J**; Weghoff et al., 2015). Bf-Ldh was first identified in *A. woodii* and Ldh and EtfAB contain flavin binding motifs (**Figure 2J**) (Weghoff et al., 2015). In addition to flavin binding motifs, EtfA also encodes a [4Fe-4S] cluster binding motif, and EtfB potentially houses the Bf flavin. Previous studies have found that Ldh and EtfAB tend to be co-localized in genomes (Weghoff et al., 2015), which guided our flanking gene analyses (**Table 1**).

#### F_420_H_2_-Dependent Heterodisulfide Reductase (Hdr2)

HdrABC are components of Bf Fdh and Mvh complexes. A functionally distinct paralog of HdrABC was discovered in *Methanosarcina acetivorans* (Buan and Metcalf, 2010) that was recently shown to function alone by coupling the oxidation of coenzyme F_420_H_2_ with the reduction of Fd^+^ and heterodisulfide from coenzyme M (CoM) and coenzyme B (CoB) (termed Hdr2) (**Figures 1K**, **2K**, Yan et al., 2017). Like the Hdr complex identified in the Fdh and Mvh complexes, Hdr2 is comprised of three subunits that include the large Bf subunit Hdr2A that contains motifs to bind FAD, four [4Fe-4S] clusters, and one [2Fe-2S] cluster. Hdr2B and Hdr2C contain motifs for coordinating one [4Fe-4S] and two [4Fe-4S] clusters, respectively (**Figure 2K**). Like HdrABC, the catalytic subunit of Hdr2A is not co-localized [as is the case for *M. acetivorans* (Yan et al., 2017)]. Furthermore, multiple copies of HdrA have previously been shown to exist in a single genome (Buan and Metcalf, 2010; Yan et al., 2017). Therefore, we classified HdrABC as Hdr2ABC only if the genome contained extra copies of HdrBC that were unaccounted for after considering the presence of other complexes that HdrABC forms associations with (i.e., Mvh, Fdh and Met). Metagenomes typically comprise multiple organisms which makes it difficult to assess whether the Hdr2A is from the same genome as the Hdr2BC subunits and whether an identified Hdr complex functions alone or in complex with Mvh, Fdh, or Met. For these reasons, we did not assess the distribution of Hdr2 in metagenomes.

#### Methylene-H_4_F Reductase/Heterodisulfide Reductase (Met)

Methylene-tetrahydrofolate (H_4_F) reductase, in its simplest form (i.e., as a single subunit), catalyzes the reduction of methylene-H_4_F with reducing power from NADH (Guenther et al., 1999). It can also couple with other subunits that include MetV or Rnf where it then functions in a variety of anabolic and catabolic reactions (Bertsch et al., 2015). Furthermore, it has been shown to interact with HdrABC to form a Bf complex (termed Met), first identified in *Moorella thermoacetica* (Mock et al., 2014). In addition to HdrABC, the Bf Met complex comprises the three subunits, MetF, MetV, and MvhD (**Figure 1L**). MetF contains a flavin binding site whereas MetV and MvhD encode motifs predicted to ligate a [4Fe-4S] cluster and a [2Fe-2S] cluster, respectively (**Figure 2L**) (Bertsch et al., 2015). Importantly, MetF that forms a Bf complex contains a motif involved in flavin binding that is different from that found in MetF from *Escherichia coli* which does not form a Bf complex (Bertsch et al., 2015). Hence, MetF homologs were aligned and only sequences that encoded the specified conserved residues as described in (Bertsch et al., 2015) were retained for downstream analysis. A similar approach to that described above for Mvh and Fdh was used to identify homologs of Met in metagenomes. MetFVD are co-localized, but are not co-localized with HdrABC. Since genes encoding for HdrABC are often not co-localized with those coding for MetFVD, we assumed that if MetFVD were detected that HdrABC were also likely to be encoded in that same genome in our metagenomic screens.

### Statistical Analysis

A binary table was created to represent the presence or absence of specified Bf enzymes in each genome and metagenome. The binary table was used as input to generate an abundance plot and was also subjected to co-occurrence analysis using the co-occur package in R (Griffith et al., 2016).

### Phylogenetic Analysis

All 16S ribosomal RNA (rRNA) genes from genomes that encoded at least one homolog of a Bf enzyme were compiled via BLASTn. The extracted sequences were subjected to multiple sequence alignment using the SILVA rRNA gene database (Quast et al., 2013) as an alignment reference and the mothur program (version. 1.39.5) (Schloss et al., 2009). The aligned sequences were filtered to remove all gaps and incomplete 16S rRNA gene sequences as previously described (Lindsay et al., 2017). The filtered 16S rRNA gene sequence alignment block was used to generate a phylogenetic tree with RAxML (version 7.3.0) (Stamatakis, 2014) specifying the LG substitution matrix and the GTRGAMMA option to cluster the sequences into unique groups. FigTree (version 1.4.2) (Bogaardt, 2014) was used to visualize the tree.

To further define the potential taxonomic origin of the 12 Bf enzyme complexes among extant organisms with available genome sequences, and to identify environment types that harbor organisms with the most deeply rooted homologs of each of the 12 Bf complexes, we also subjected our curated database of oxidoreductase catalytic subunit homologs (i.e., HydA, HytA, MvhA, FdhA, FdhF2, NfnSL, FixAB, Bcd, CarC, HylA, Ldh, and MetF) from complete genomes and metagenomes to phylogenetic analysis. We did not conduct phylogenetic analysis of the catalytic subunit of Hdr homologs (i.e., HdrA and Hdr2A) identified in metagenomes because of difficulty in identifying whether the subunits of Hdr2 (i.e., HdrABC) were from the same genome and if they function alone or in association with Met, Mvh, or Fdh, as described above. Likewise, while phylogenetic analyses were conducted on HdrA, we did not assign homologs as belonging to Met, Fdh, Mvh, or Hdr2 complexes since it is not possible to link these functions based on genome context. Briefly, paralogs (described below) of each of the aforementioned catalytic subunits were identified and the curated putative Bf homologs were aligned using Clustal Omega (Sievers et al., 2011). The aligned sequences were then subjected to phylogenetic reconstruction using RAxML specifying the LG substitution matrix and the PROTGAMMA option to empirically cluster the sequences into unique groups. Itol was used to visualize the trees (Letunic and Bork, 2016).

Paralogs of the catalytic subunits for each Bf enzyme complex have already been identified in previous studies for many of the enzymes and these served as outgroups in phylogenetic reconstructions. These include the eukaryotic Nar-like protein that lacks the conserved L1, L2, and L3 motifs present in HydA/HytA (Meyer, 2007; Wang et al., 2010), non-Bf [NiFe]-hydrogenase large subunits from group 3d for MvhA (Boyd et al., 2014), the PyrK subunit of dihydroorotate dehydrogenase and the beta subunit of glutamate synthase, for Bf NfnSL, respectively (Demmer et al., 2015), the non-Bf group 5 EtfBA for FixAB (Garcia Costas et al., 2017), thioredoxin reductase for HdrA (Hedderich et al., 1994), and non-Bf MetF for Bf MetF (Bertsch et al., 2015). Paralogs of the remaining catalytic subunits (FdhA, FdhF2, Bcd, CarC, and Ldh) were empirically determined by subjecting the representative catalytic sequences to BLASTp against the non-redundant protein database of NCBI. FdhA and FdhF2 are closely related paralogs and thus were used as outgroups for each other in phylogenetic reconstructions. Likewise, Bcd and CarC are closely related paralogs and thus were used as outgroups for each other in phylogenetic reconstructions. The most closely related sequence to Ldh was identified as alkyl dihydroxyacetone phosphate synthase, and this was used as an outgroup for Ldh in phylogenetic reconstructions.

The numbers of Nfn and Met homologs in metagenomes were much larger (i.e., >1,500 homologs) than those associated with other classes of Bf enzymes. Therefore, to reduce the computational time necessary to analyze the evolutionary history of homologs of these two enzymes, we first clustered them into homolog ‘bins’ using CD-HIT (Li and Godzik, 2006; Fu et al., 2012) at the 60% sequence identity level. As such, each unique bin contained closely related homologs. Representative sequences from each bin of NfnSL and MetF were used in the phylogenetic analyses and we then cross-referenced the sequences in the phylogeny with those within the bins to identify patterns in the distribution of homologs on the final tree and the environment types that hosted those homologs.

### Pairwise Sequence Identity

To determine the extent to which individual subunits comprising each of the Bf complexes have co-evolved, we first aligned homologs of individual subunits using Clustal Omega (Sievers et al., 2011). Pairwise distances using the p-distance model (Kumar et al., 2016) were calculated in MEGA (Tamura et al., 2007), and a dissimilarity matrix was generated using these distances for each specified protein subunit. Mantel tests were performed to calculate matrix correlations and their statistical significance with the R package ape (Paradis et al., 2004; R Core Team, 2014).

Lastly, we determined the variance in amino acid identities of homologs of the catalytic subunits of Bf complexes identified among the complete genome datasets as a proxy for the functional diversity of those homologs. Pairwise sequence comparisons of the homologs of each Bf enzyme catalytic subunit identified in complete genomes were used to calculate pairwise E-values using phmmer. E-values identified for each pairwise comparisons were used as a proxy for amino acid identity differences. E-values were normalized by multiplying by -10^4^ and are presented as this transformed value. High E-values indicate that homologs exhibit a lower diversity and thus are more similar phylogenetically. Low E-values indicate that homologs are more diverse and are less similar phylogenetically. We then subjected homologs of the catalytic subunits of Bf complexes identified among the metagenome datasets to pairwise sequence comparisons against the dataset comprising the homologs identified in genome sequences using phmmer. This analysis was performed to assess the extent that homologs identified in the complete genome dataset adequately captured the natural diversity detected in metagenomes for each Bf enzyme. Homologs identified in metagenomes that exhibit a lower average or range of E-values than those identified in complete genomes are underrepresented in complete genome databases (i.e., there exists unsampled diversity).

## Results and Discussion

### Distribution of Electron Bf Enzyme Homologs in Genome Sequences

Of the total 4,588 archaeal, bacterial, and eukaryal genomes available, 681 (15%) coded for at least one homolog of a Bf enzyme (Supplementary Table S1). Of these 681 genomes, 169 were from Archaea while 512 were from Bacteria while genomes from Eukarya did not code for homologs of any of the 12 FBEB enzymes. Among Archaea, homologs of Bf enzymes were detected in genomes from three out of four phyla considered in our database: Euryarchaeota (*n* = 126 genomes), Crenarchaeota (*n* = 42 genomes), and the candidate division Korarchaeota (*n* = 1 genome). The majority (83%) of Bf enzyme homologs were identified in genomes from the bacterial phyla Firmicutes (*n* = 184 genomes), Proteobacteria (*n* = 147 genomes), Bacteroidetes (*n* = 44 genomes), Thermotogae (*n* = 25 genomes), and Spirochaetes (*n* = 22 genomes) (Supplementary Table S1).

We also classified organisms whose genomes coded for Bf enzyme homologs as a function of their ability to integrate oxygen (O_2_) into their energy metabolism based on information acquired from the DOE-IMG database and previous physiological characterizations. Of the 681 identified organisms whose genomes encoded at least one Bf enzyme homolog, 16% (*n* = 110 genomes) were aerobes, 9% (*n* = 59 genomes) were facultative anaerobes, and 74% (*n* = 503 genomes) were strict anaerobes. The prevalence of Bf enzyme homologs among obligate anaerobes is consistent with our hypothesis suggesting the importance of FBEB for organisms inhabiting energy limited environments.

#### [FeFe]-Hydrogenase (Hyd)

**FIGURE 3:**
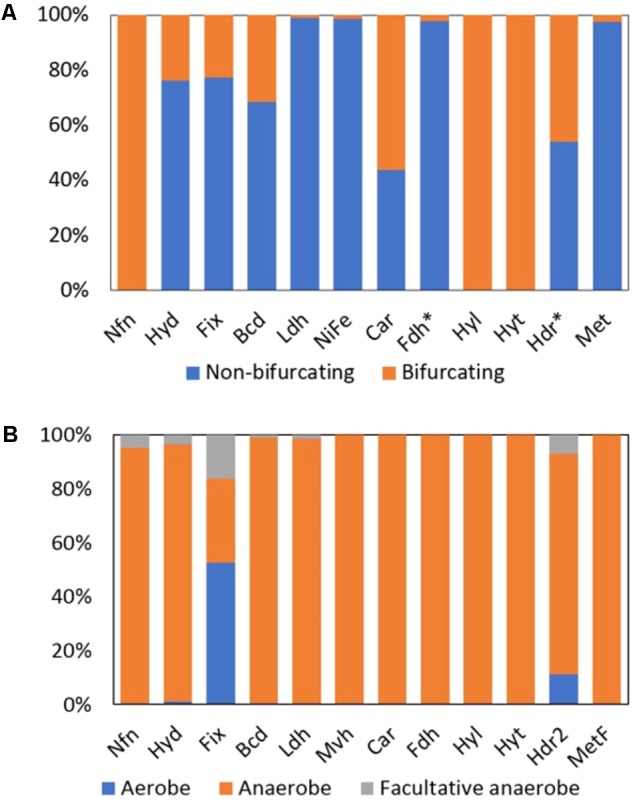
Distribution of homologs of putative Bf and non-Bf enzymes in complete genome sequences. **(A)** Histogram depicting the percent of homologs that are predicted to bifurcate electrons and those that are predicted to not bifurcate electrons in complete genomes. **(B)** Percent of enzyme homologs predicted to bifurcate electrons that are derived from the genomes of aerobes, anaerobes, or facultative anaerobes. Fdh^∗^ and Hdr^∗^ includes all the Bf enzymes that form a complex with Fdh (i.e., Hyt, Hyl, and Hdr associated Fdh) and Hdr (i.e., Fdh associated Hdr, Mvh, Hdr2, and Met), respectively. Names of each abbreviated enzyme complex are provided in **Table 1**. Note, Mvh are the only known Bf subclass of [NiFe]-hydrogenase (labeled [NiFe] in **(A)**).

Among the total 961 Hyd (putatively Bf and non-Bf) homologs detected in complete genome sequences, 24% were multimeric (i.e., HydABC or HydABCD) and hence can potentially bifurcate electrons (**Figure 3A**). In total, 229 homologs of Bf Hyds were identified in complete genome sequences, of which 95% were from the genomes of anaerobes, 4% were from the genomes of facultative anaerobes, and 1% were from the genomes of putative aerobes that belong to the phylum Spirochaetes [e.g., *Turneriella parva* DSM 21527 (AFM13386) and *Salinispira pacifica* (AHC13718)] (**Figure 3B**); the ability of *T. parva* and *S. pacifica* to use O_2_ has not been robustly tested. The genomes that coded for homologs of Bf Hyds belonged exclusively to the bacterial domain, which is consistent with previous studies (Meyer, 2007; Calusinska et al., 2010; Mulder et al., 2010; Peters et al., 2015; Poudel et al., 2016).

Hyd is one of three evolutionarily distinct hydrogenases that catalyze the reversible reduction of protons to hydrogen (H_2_) (Vignais et al., 2001; Peters et al., 2015). Bf Hyds couple the simultaneous and reversible reduction of NAD^+^ and Fd^+^ to the oxidation of H_2_ (**Figure 2A**; Schut and Adams, 2009; Schuchmann and Muller, 2012). Bf Hyd thus plays a key role in balancing the ratio of oxidized to reduced NAD(H) and Fd pools in the cell. Although Bf Hyd homologs were primarily identified among the genomes of Firmicutes (63% of the total genomes) and Thermotogae (13% of the total genomes) (Supplementary Table S2), they were also identified, albeit sporadically, among a wide diversity of organisms (Supplementary Figure S1). These organisms are supported by a variety of metabolisms that include metal reduction (e.g., *Alkaliphilus metalliredigens* QYMF), arsenate reduction (e.g., *Alkaliphilus oremlandii* OhILAs), nitrate reduction (e.g., *Clostridium perfringens*), sulfur reduction (e.g., *Pelobacter carbinolicus*), and sulfate reduction (e.g., *Desulfotomaculum acetoxidans* DSM 771), although it is not necessarily clear if these homologs are active under these physiological conditions. Moreover, homologs of the Bf Hyd were identified in the genomes of organisms that inhabit a range of environments based on where they were isolated from, including those that are of moderate (e.g., *Clostridium beijerinckii*) to high temperature (e.g., *T. maritima*).

#### [NiFe]-Hydrogenase (Mvh)

Only 1% of the total 3,808 homologs of [NiFe]-hydrogenase that were identified (putatively Bf and non-Bf) in complete genomes were predicted to bifurcate electrons based on those genomes also coding for MvhADG and HdrABC and the presence of unique sequence motifs in MvhA (**Figure 3A**). Mvh homologs were identified in the genomes of obligate anaerobes (**Figure 3B**) and were largely confined to members of the archaeal domain (74% of the total Mvh encoding genomes) with the remaining 26% of the genomes that encode Mvh being from members of the bacterial domain (Supplementary Table S2).

Bf Mvh couples the simultaneous and reversible reduction of Fd^+^ and heterodisulfide from CoM-CoB to the oxidation of H_2_ (**Figure 2B**; Thauer et al., 2008, 2010; Kaster et al., 2011). Mvh was primarily identified in the genomes of methanogens that belong to the phylum Euryarchaeota (Supplementary Table S2), although homologs were also detected in the genomes of sulfate-reducing bacteria (e.g., *Thermodesulfatator indicus* DSM 15286), halophilic bacteria (e.g., *Desulfohalobium retbaense* DSM 5692), ammonifying bacteria (e.g., *Ammonifex degensii* KC4), and sulfate-reducing archaea (e.g., *Archaeoglobus profundus* DSM 563). This suggests that the reversible reduction of Fd^+^ and CoM-CoB with H_2_ may be involved in a diversity of metabolisms (Supplementary Figure S1).

#### Heterodisulfide-Linked Formate Dehydrogenase (Fdh)

Formate dehydrogenase can form a Bf complex with (i) a Hdr complex (termed Fdh; **Table 1**), (ii) a [FeFe]-hydrogenase-like complex (termed Hyl), and (iii) a NADP(H)-dependent [FeFe]-hydrogenase complex (termed Hyt). Among the total 2,516 homologs of formate dehydrogenase that were detected in available complete genomes, 2% were predicted to form a complex that would allow for bifurcation of electrons (either as Fdh, Hyl, or Hyt); the remaining formate dehydrogenase homologs are unlikely to bifurcate electrons and likely function as canonical formate dehydrogenases which reversibly oxidize formate to CO_2_ (**Figure 3A**; Ferry, 1990). Among the putative Bf Fdh, 73% were associated with Hdr (termed Fdh), all of which were from obligate anaerobes (**Figure 3B**) that were primarily within the archaeal phylum Euryarchaeota (Supplementary Table S2).

Fdh couples the simultaneous and reversible reduction of Fd^+^ and heterodisulfide from CoM-CoB to the oxidation of formate (**Figure 2C**; Costa et al., 2013). Interestingly, genes encoding homologs of Fdh were primarily detected in the genomes of methanogens and in the genomes of only two sulfate-reducing Proteobacteria, *Desulfobacterium autotrophicum* HRM2 and *Desulfobacula toluolica* Tol2 (Supplementary Figure S1). The role of putative Bf Fdh enzymes in the metabolism of these sulfate reducing bacterial taxa is not known.

#### NADP(H)-Dependent Formate Dehydrogenase (Hyt)

In total, the genomes of five acetogenic and obligately anaerobic bacteria that belong to the genus *Clostridium* within the phylum Firmicutes encoded Hyt (**Figures 3A,B** and Supplementary Table S2). Hyt couples the simultaneous and reversible reduction of Fd^+^ and NADP^+^ to the oxidation of formate or H_2_ (**Figure 2D**; Wang et al., 2013a). Formate is suggested to bind to FdhA and it is thought that either formate or H_2_ can serve as an electron donor to the Hyt complex (Wang et al., 2013a). However, it is not clear from previous biochemical characterizations where Fd, NADH, and H_2_ bind in the complex. Several of the subunits that comprise Hyt (i.e., HytABC) are homologous to HydABC of the Bf [FeFe]-hydrogenase complex (Hyd). However, Hyt appears to be specific for NADP(H) whereas Hyd utilizes NAD(H) (Wang et al., 2013a). Nonetheless, based on homology, we suggest that H_2_ binds to HytA in a manner like H_2_ binds to HydA (Peters et al., 1998), Fd binds to HytC in a manner like Fd binds to HydC (Schut and Adams, 2009), and NADP(H) binds to HytB in a manner like NAD(H) binds to HydB (Schut and Adams, 2009) (**Figure 2D**). The roles of HytE1, HytE2, and HytD in the functioning of Hyt are not presently known, however, they harbor conserved cysteine residues that may serve as ligands for FeS clusters. This may suggest that they are involved in electron transfer within the enzyme complex.

#### NAD(H)-Dependent Reduced Ferredoxin:NADP Oxidoreductase (Nfn)

There are several classes of Nfn that have been termed NfnI, NfnII (also termed Xfn), and NfnIII, which are all proposed to bifurcate electrons (Nguyen et al., 2017). However, the high potential electron acceptor in NfnII is not defined but is known to not be NAD(H) like in NfnI. Based on this precedent in the literature, all Nfn identified in genome sequences herein are proposed to bifurcate electrons (**Figure 3A**). Of the 397 archaeal and bacterial genomes that encoded Nfn, 72 were from Archaea and 325 were from Bacteria. The majority (82%) of the archaeal and bacterial genomes that coded for Nfn belonged to the bacterial phyla Firmicutes (*n* = 138 genomes), Proteobacteria (*n* = 53 genomes), Bacteroidetes (*n* = 44 genomes), Thermotogae (*n* = 24 genomes), and the archaeal phylum Euryarchaeota (*n* = 66 genomes) (Supplementary Table S2). All the genomes that encoded Nfn were from anaerobic organisms with the exception of *Candidatus* Koribacter versatilis Ellin345 (ABF41796), which has been characterized as an aerobe (**Figure 3B** and Supplementary Table S2) (Sait et al., 2002).

Nfn is one of the four unique Bf enzymes whose crystal structure has been solved (Demmer et al., 2015; Lubner et al., 2017). Biophysical data collected on Nfn from *Pyrococcus furiosus* shows that flavin is indeed the site of bifurcation (Lubner et al., 2017). Nfn catalyzes the simultaneous and reversible reduction of NAD^+^ and Fd^+^ with the oxidation of NADPH (**Figure 2E**; Demmer et al., 2015; Lubner et al., 2017) and hence plays a critical role in balancing the ratio of oxidized and reduced pyridine [(i.e., NAD(H) and NADP(H)] and Fd pools. Our taxonomic distribution data strongly suggests that Nfn is present in a diversity of microorganisms that operate a variety of metabolisms in diverse environmental settings. For example, Nfn is encoded in the genomes of strict anaerobes and facultative anaerobes (and possibly an aerobe, as described above) and in mesophilic and thermophilic taxa (Supplementary Figure S1). Moreover, Nfn was identified in the genomes of organisms that catalyze methanogenesis (e.g., *Methanosarcina barkeri*), acetogenesis (e.g., *M. thermoacetica*), elemental sulfur reduction (e.g., *Desulfurococcus mucosus*, *Thermosulfidibacter takaii*), dinitrogen reduction (e.g., *Rhodopseudomonas palustris*), heterotrophy (e.g., *P. furiosus*), and phototrophy (e.g., *Chlorobium tepidum*, *R. palustris*), among others (Supplementary Table S2).

#### Electron Transfer Flavoprotein Involved in Nitrogen Fixation (Fix)

Among the 930 Etf homologs identified in sequenced genomes, 19% were demarcated as Fix and are predicted to bifurcate electrons (**Figure 3A**). Unlike other Bf enzymes that are largely confined to anaerobic or facultatively anaerobic taxa, the majority (53% of total) of the homologs of Fix were identified in the genomes of aerobes (**Figure 3B**). Of the 178 genomes that coded for homologs of Fix, 25% were from Archaea within the phylum Crenarchaeota (*n* = 36) (Supplementary Table S2). The remaining 75% of the Fix encoding genomes were from Bacteria within the phyla Proteobacteria (*n* = 85 genomes) and Firmicutes (*n* = 33) (Supplementary Table S2).

A recent structural bioinformatics and phylogenetics study categorized Etfs into five unique groups, of which only group 2 contained putative Bf Etfs (Garcia Costas et al., 2017). Formally, Etf enzyme complexes that are involved in supplying Fd^-^ for use in nitrogen fixation (diazotrophy) are termed Fix (Ledbetter et al., 2017) and these belong to group 2D2 (Garcia Costas et al., 2017). Fix catalyzes the simultaneous and reversible reduction of Fd^+^ and quinone to the oxidation of NADH (**Figure 2F**; Earl et al., 1987; Edgren and Nordlund, 2004; Ledbetter et al., 2017). Additional Fix homologs were identified among group 2 Etf that have the subunit architecture and sequence motifs that suggest an ability to bifurcate. These were identified in the genomes of elemental sulfur oxidizing aerobic archaea (e.g., *Sulfolobus islandicus*), iron reducing anaerobic archaea (e.g., *Pyrobaculum islandicum*), anaerobic acetogenic bacteria (e.g., *M. thermoacetica*), anaerobic sulfate-reducing bacteria (e.g., *Caldimicrobium thiodismutans*), and anaerobic fermentative thermophilic bacteria (e.g., *T. maritima*) (Supplementary Figure S1). The role of these enzymes in the physiology of these cells is not known, nor has it been biochemically shown that these homologs bifurcate electrons. However, for simplicity we have designated all Fix-like Etf (that include Etf identified in diazotrophs and non-diazotrophs) as Fix in this study.

#### Electron Transfer Flavoprotein/Butyryl-CoA Dehydrogenase (Bf-Bcd)

Butyryl-CoA dehydrogenase (Bcd) can form a complex with EtfAB and bifurcate electrons (Herrmann et al., 2008). In total, 309 homologs of Bcd were identified among genome sequences and 31% of these are predicted to form a complex with EtfAB (termed Bf-Bcd; **Table 1**) and hence can potentially bifurcate electrons (**Figure 3A**). Ninety-eight percent of Bf-Bcd encoding genomes were from anaerobic taxa (**Figure 3B**). Bf-Bcd homologs were confined to Bacteria where the enzyme was detected in the phyla Firmicutes (*n* = 56 genomes), Fusobacteria (*n* = 15 genomes), and Bacteroidetes (*n* = 12 genomes) (Supplementary Table S2).

Bf-Bcd was the first enzyme reported to be able to bifurcate electrons (Herrmann et al., 2008; Li et al., 2008). Composed of only three subunits (i.e., EtfAB and Bcd), the enzyme complex was shown to couple the simultaneous reduction of Fd^+^ and crotonoyl-CoA to the oxidation of NADH during clostridial fermentation (**Figure 2G**; Chowdhury et al., 2014). Thermodynamically, the reaction should be reversible; however, the enzyme has not been shown to be reversible yet. In addition to Clostridia, pathogenic bacteria from the phyla Bacteroidetes (e.g., *Porphyromonas gingivalis*), Firmicutes (e.g., *Clostridium botulinum*), and Fusobacteria (e.g., *Fusobacterium nucleatum*) encoded Bf-Bcd. In addition, homologs of Bf-Bcd were detected in the genomes of metal reducers (e.g., *A. metalliredigens* QYMF), acetogens (e.g., *C. beijerinckii*), and thermophilic heterotrophs (e.g., *Fervidobacterium nodosum*) (Supplementary Figure S1).

#### Electron Transfer Flavoprotein/Caffeyl-CoA Reductase (Car)

Caffeyl-CoA reductase was identified by itself (i.e., CarC) or in complex with Etfs (i.e., CarCDE) (Bertsch et al., 2013). In total, 41 CarC homologs were identified and 56% of these are predicted to form a complex with CarDE (termed Car; **Table 1**), and thus are predicted to bifurcate electrons (**Figure 3A**). The Car complexes were identified in the genomes of anaerobic bacteria (**Figure 3B**), primarily within the phylum Firmicutes (Supplementary Table S2).

The Car complex was first described in *A. woodii* where it was shown to couple the simultaneous and reversible reduction of Fd^+^ and caffeyl-CoA with the oxidation of NADH (**Figure 2H**; Bertsch et al., 2013). Although it was first described in an acetogenic bacterium, data presented here indicates that Car may also function in the metabolism of an arsenite oxidizer (e.g., *A. oremlandii* OhILAs), a fermentative halophile (i.e., *Halanaerobium praevalens*), and in several fermentative thermophiles within the Thermotogales (e.g., *Thermosipho africanus*), among others (Supplementary Figure S1), based on the presence of homologs encoded in these organisms’ genomes.

#### NAD(H)-Dependent Formate Dehydrogenase (Hyl)

As previously mentioned, formate dehydrogenases can function alone or form a complex with Hdr, Hyt, or a trimeric hydrogenase-like complex comprising HylABC (termed Hyl; Wang et al., 2013b). Of the 2,516 formate dehydrogenase homologs identified, 19% are predicted to form a complex with HylABC (**Figure 3A**) and these homologs were detected only in the genomes of anaerobes (**Figure 3B**) within the phylum Firmicutes (Supplementary Table S2).

Hyl couples the simultaneous and reversible reduction of Fd^+^ and NAD^+^ with the oxidation of formate (**Figure 2I**; Wang et al., 2013b). Based on biochemical characterization of this complex (Wang et al., 2013b), it is not yet clear where substrates bind. However, the subunits of Hyl (i.e., HylABC) are homologous with those of the Bf Hyd (HydABC) with the exception that HylA lacks three conserved motifs (L1, L2, and L3) that are involved in ligating the H cluster in HydA (Meyer, 2007). Thus, it is possible that Fd binds to HylC in a manner like Fd binds to HydC and NAD(H) binds to HytB in a manner like NAD(H) binds to HydB (Schut and Adams, 2009; Schuchmann and Muller, 2012). Given the presence of conserved cysteine motifs, it is possible that HylA plays a role in electron transfer. Hyl was detected in the genomes of fermentative bacteria (e.g., *Clostridium acetobutylicum*), metal reducers (e.g., *A. metalliredigens* QYMF), arsenite oxidizers (e.g., *A. oremlandii* OhILAs), acetogens (e.g., *M. thermoacetica*), sulfate-reducers (e.g., *D. acetoxidans* DSM 771), and thermophilic heterotrophs (e.g., *Thermacetogenium phaeum* DSM 12270) (Supplementary Figure S1).

#### Electron Transfer Flavoprotein/Lactate Dehydrogenase (Bf-Ldh)

Lactate dehydrogenase (Ldh) can form a complex with EtfAB (termed Bf-Ldh; **Table 1**) and bifurcate electrons (Weghoff et al., 2015). Among the total 6,158 homologs of Ldh that were identified, 1% are predicted to form a complex with EtfAB and thus potentially bifurcate electrons (**Figure 3A**). The Bf-Ldh homologs identified belonged to the bacterial domain and were from either obligate anaerobes or facultative anaerobes (**Figure 3B**).

Bf-Ldh couples the reduction of Fd^+^ and pyruvate with oxidation of NADH (**Figure 2J**; Weghoff et al., 2015). Most Bf-Ldh homologs were detected among genomes from the phylum Firmicutes (*n* = 55) and these included metal reducers (e.g., *A. metalliredigens* QYMF), acetogens (e.g., *A. woodii*), sulfate-reducers (e.g., *Desulfosporosinus acidiphilus* SJ4), and anaerobic phototrophs (e.g., *Heliobacterium modesticaldum* Ice1) (Supplementary Figure S1).

#### F_420_H_2_-Dependent Heterodisulfide Reductase (Hdr2)

A total of 770 homologs of heterodisulfide reductase (HdrABC) were identified, of which 46% were predicted to form a complex with either FdhAB, MvhAGD, or MetFV (described below) or to function alone (termed Hdr2; **Table 1**). In total, 63% (*n* = 226 homologs) of the HdrABC homologs identified were identified in genomes that encoded copies of HdrABC attributable to Fdh, Mvh, or Met complexes. The extra copies of HdrABC in these genomes were thus designated as Hdr2 (**Figure 3A**). Hdr2 homologs were primarily identified in genomes from the phyla Euryarchaeota (*n* = 66 genomes), Proteobacteria (*n* = 44 genomes) and Firmicutes (*n* = 25 genomes) (Supplementary Table S2). Furthermore, these homologs were identified in the genomes of anaerobes (i.e., 80% of the total genomes), aerobes (i.e., 7% of the total genomes) and facultative anaerobes (10% of the total genomes) (**Figure 3B**).

Hdr2 couples the reduction of Fd^+^ and CoM-CoB with the oxidation of coenzyme F_420_H_2_ (**Figure 2K**; Yan et al., 2017). Hdr2 homologs were detected in methanogens (e.g., *M. acetivorans*), as previously noted (Yan et al., 2017), acetogens (e.g., *M. thermoacetica*), sulfate-reducers (e.g., *D. acidiphilus* SJ4), and anaerobic phototrophs (e.g., *H. modesticaldum* Ice1) suggesting that it is widely distributed among organisms with diverse metabolic backgrounds (Supplementary Figure S1). While HdrABC complexes are widely found in strict anaerobes (Thauer et al., 2008; Buan and Metcalf, 2010; Yan et al., 2017), we were surprised to detect homologs of Hdr2 in the genomes of aerobic and microaerophilic organisms. These organisms primarily belonged to the phylum Aquificae that include *Aquifex aeolicus*, *Hydrogenobaculum* sp., and *Thermocrinis albus* (Supplementary Table S2). However, a recent study showed that the HdrABC (i.e., Hdr2 since it could not be accounted for by Fdh, Mvh, or Met) in *Aquifex aeolicus* was expressed in cultures grown with thiosulfate or elemental sulfur leading to the speculation that Hdr could be involved in sulfur oxidation through an undefined mechanism (Boughanemi et al., 2016).

#### Methylene-H_4_F Reductase/Heterodisulfide Reductase (Met)

A total of 1,017 methylene-H_4_F reductase homologs were identified and 3% (*n* = 25 homologs) of these were from genomes that also encoded MetFV, MvhD, and HdrABC. Thus, these homologs can putatively bifurcate (termed Met; **Table 1**) (**Figure 3A**). Homologs of Met were primarily confined to the phyla Firmicutes (*n* = 12 genomes) and Proteobacteria (*n* = 8 genomes) and all were from obligate anaerobes (**Figure 3B**).

The Bf Met complex couples the reduction of methylene-H_4_F to the oxidation of NADH and a yet to be identified electron acceptor that is thought to be Fd^+^ (**Figure 2L**; Mock et al., 2014). Met homologs were also detected in a methanogen (i.e., *Methanomassiliicoccus intestinalis*), acetogens (e.g., *M. thermoacetica*), sulfate-reducers (e.g., *D. acidiphilus* SJ4), and nitrate reducers (e.g., *A. degensii*) (Supplementary Figure S1), suggesting a widespread distribution in anoxic habitats.

#### Putative Interactions Among Bf Enzymes

Homologs of multiple (up to seven) different Bf enzymes were detected in a single genome and this was particularly true for organisms belonging to the phyla Firmicutes, Thermotogae, and Euryarchaeota (Supplementary Figure S2 and Supplementary Table S2). Among Firmicutes genomes, varying combinations of homologs of Hyd, Bf-Ldh, Car, Bf-Bcd, and Nfn were often detected whereas among Thermotogae genomes it was common to detect varying combinations of the homologs of Hyd, Nfn, and Fix. Likewise, euryarchaeote genomes, in particular those from methanogens, often encoded a combination of Nfn, Mvh, Hdr2, and Fdh.

**FIGURE 4:**
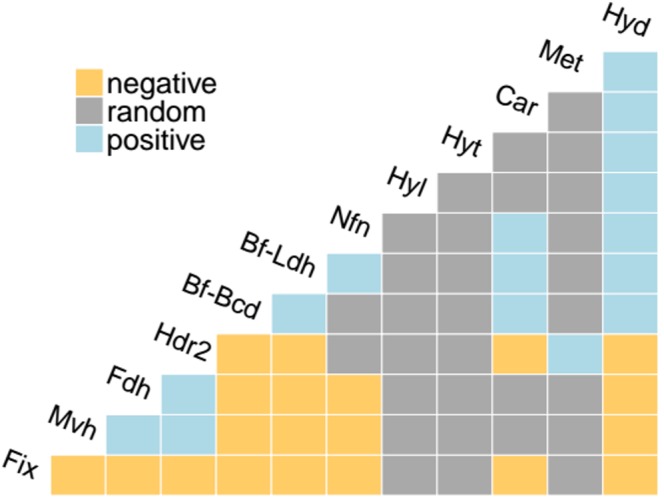
Co-occurrence analysis of the distribution of homologs of Bf enzymes in complete genome sequences. Positive co-occurrences (exceeding the 95% statistical significance threshold) are represented in cyan, negative co-occurrences are represented in yellow, and random co-occurrences are represented in gray. Abbreviations for enzyme complexes are provided in **Table 1**.

To quantify the extent that homologs of the 12 Bf complexes co-distribute in complete genome sequences, we conducted a co-occurrence statistical analysis. Fdh, Mvh, and Hdr2, which are primarily found in methanogens (**Figures 2B,C,K**, respectively), positively co-occurred (**Figure 4**). This is consistent with biochemical data that indicates both [NiFe]-hydrogenase (MvhAG) and formate dehydrogenase (FdhAB) compete to bind MvhD that is associated with HdrABC (Costa et al., 2013). Maintaining homologs of both MvhAG and FdhAB likely allows the host to generate reduced Fd in environments with a dynamic supply of H_2_ or formate (Costa et al., 2010, 2013). Hdr2 was also positively associated with the distribution of Mvh and/or Fdh in genomes (**Figure 4**), consistent with previous reports (Buan and Metcalf, 2010; Yan et al., 2017).

The distributions of Bf-Ldh, Car, Bf-Bcd, Hyd, and Nfn, all of which were primarily detected in bacterial genomes, were also positively correlated (**Figure 4**). Importantly, since these five enzymes share common substrates [i.e., NAD(H) and Fd], several authors have proposed that the reductant generated from one Bf enzyme can be utilized by another Bf enzyme (Bertsch et al., 2013; Buckel and Thauer, 2013; Weghoff et al., 2015), thereby allowing for tight control on the balance of oxidized/reduced substrates/products in a cell. For example, it has been proposed that Car partially relies on Hyd to generate NADH for use in reducing caffeyl-CoA to hydrocaffeyl-CoA, and in the process Fd^-^ is generated (Bertsch et al., 2013). The membrane bound Rnf complex, which catalyzes the reversible NAD^+^-dependent oxidation of Fd^-^ by coupling to the electrochemical gradient (Biegel et al., 2011; Sarkar et al., 2012; Hess et al., 2013) could use the Fd^-^ to generate more NADH which can then feed into the bifurcation reaction carried out by Car (Bertsch et al., 2013). By coordinating these Bf enzymes and Rnf, oxidants (NAD^+^, Fd^+^) are generated and caffeyl-CoA is kept from building up, thereby allowing for metabolism to continue (Bertsch et al., 2013).

We further examined the co-distribution of Bf-Ldh, Car, Bf-Bcd, Hyd, and Nfn in complete genome sequences to identify evidence for potential interactions between these Bf complexes. Genomes that encode Bf-Bcd often also encode Nfn (68% of the Bf-Bcd encoding genomes) and Hyd (46% of the Bf-Bcd encoding genomes) (Supplementary Table S1) suggesting Fd^-^ generated during the reduction of crotonyl-CoA may be used by either Nfn or Hyd to generate either NADPH or H_2_, respectively. The bifurcation reaction for Nfn and confurcation reaction of Hyd requires NADH and this has been suggested to be provided through glycolytic reactions (Buckel and Thauer, 2013). Other examples of co-distributed Bf enzyme homologs are presented in Supplementary Table S2. Together, these observations suggest that coupling multiple redox reactions through Bf reactions allows microorganisms a continual provision of NAD^+^ or Fd^+^ to allow efficient balancing of energy metabolism in oxidant limited anoxic environments inhabited by anaerobes.

### Evolution of Bf Enzymes

#### Overview

**FIGURE 5:**
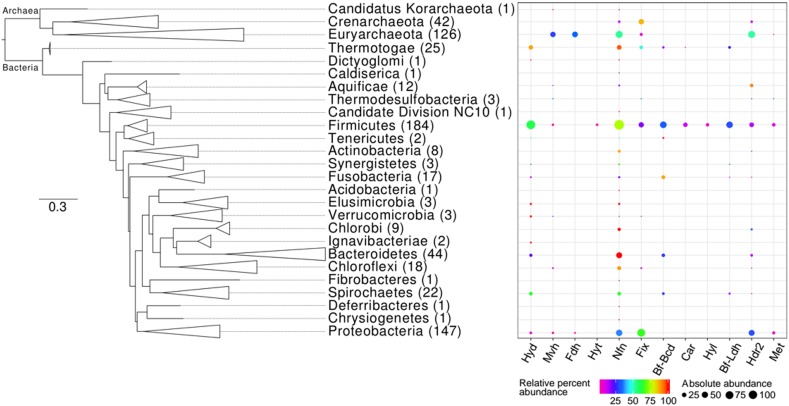
Phylogenetic distribution of homologs of enzymes that are predicted to bifurcate electrons in complete genome sequences. The Maximum-Likelihood phylogenetic tree was constructed using 16S rRNA gene sequences obtained from genomes that also encode at least one homolog of a Bf enzyme. Phylum-level lineages were collapsed to conserve space. The number in parentheses next to each phylum level designation indicates the total number of genomes within a specified lineage that code for homologs of at least one of the 12 Bf enzymes (Supplementary Table S2). The percent of genomes within a given lineage that code for a homolog of the specified Bf enzyme (i.e., relative percent abundance) is indicated by the color of the bubble. The percent of the total number of homologs of the specified Bf enzyme that are encoded in genomes affiliated with the specified lineage (i.e., absolute percent abundance) is indicated by the size of the bubble. Names for each abbreviated enzyme complex are provided in **Table 1**.

Twenty eight of the 41 phyla represented in our complete genome sequence dataset coded for at least one homolog of a Bf enzyme (Supplementary Table S2). We examined the distribution of enzyme homologs in bacterial and archaeal genomes to, at first order, provide evidence of whether these enzymes were likely a property of the LUCA (i.e., homologs present in both archaeal and bacterial domains) or if they more likely evolved after the divergence of Archaea and Bacteria from the LUCA (i.e., homologs present in only a single domain). Similar analyses have been conducted on [FeFe]-hydrogenase (Mulder et al., 2010), molybdenum-dependent nitrogenase (Boyd et al., 2011a), and mercuric reductase (Barkay et al., 2010) to determine the likelihood that these enzyme complexes were a property of the LUCA. Secondly, we reconstructed the phylogeny of 16S rRNA genes (proxy for taxonomic evolution) recovered from genomes that code for homologs of at least one of the 12 Bf enzymes for use in examining the phylogenetic distribution of Bf homologs (**Figure 5**).

Six (i.e., Nfn, Fix, Mvh, Fdh, Hdr2, and Met) of the 12 Bf enzymes were identified in genomes from both bacterial and archaeal domains indicating that they may have been a property of the LUCA. To more precisely assess this possibility, we examined (i) the extent that subunits comprising a complex co-evolved and (ii) the phylogeny of the oxidoreductase subunit for each of the Bf enzymes relative to paralogs as described below; the phylogeny of Hdr2 was not assessed (see section “Materials and Methods”). Moreover, the oxidoreductase phylogenies were used to assess if subunits that allow for bifurcation (flavoproteins) were recruited or lost (or both through multiple events) during the evolution of the oxidoreductases.

#### Hyd, Hyl, and Hyt

Hyd, Hyl, and Hyt were only detected in bacterial genomes, suggesting that these three enzymes evolved after the divergence of Archaea and Bacteria from the LUCA. This conclusion agrees with a previous analysis of the evolution of Hyd (Mulder et al., 2010), but is the first to suggest this for Hyl and Hyt. The reconstructed phylogeny reveals that monomeric, non-Bf HydA homologs predate Bf HydA, HylA, and HytA homologs with high bootstrap support (Supplementary Figure S3). This suggests that additional subunits (i.e., Hyd/Hyl/HytBC and D in the case of tetrameric Hyd) were recruited to function in complex with Hyd/Hyl/HytA, enabling FBEB.

A mantel test conducted on the pairwise distances of all the subunits of the Hyd complex (i.e., HydABC) reveals positive correlations (Supplementary Figure S4) suggesting that once these subunits were recruited they were largely maintained and were under selection to co-evolve. The earliest evolving lineage of HydA homologs were from thermophiles that belonged to the phyla Thermotogae [e.g., *T. maritima* (NP_229226) and *T. africanus* (ACJ75249)] and Dictyoglomi [e.g., *Dictyoglomus thermophilum* (ACI20052)]. These lineages were well-supported, suggesting that Hyd likely evolved and diversified in an ancestor of Thermotogae or Dictyoglomi (**Table 2**). Importantly, the taxa comprising the earliest evolving clade are thermophilic anaerobes, suggesting that Bf Hyd may have evolved in an anoxic, high temperature environment. Numerous clades of Bf HydA homologs formed interspersed lineages among monomeric non-Bf HydA lineages. The positive, strong relationships observed in the pairwise distances of HydABC (Supplementary Figure S4) suggests this observation to be the result of multiple loss events of genes encoding for HydBC rather than independent recruitment events.

**Table 2:** Proposed taxonomic origin of Bf enzyme complexes based on phylogenetic delineation of the earliest evolving extant lineage of the oxidoreductase subunits associated with each Bf enzyme complex in complete genome sequences and metagenomes (see Supplementary Figures S3, S7, S9, S12, S14, S17, S19, S20, S23–S30).

**Bifurcating System^a^**	**Complete genomes**		**Metagenomes**	
	**Earliest evolving lineage**	**Taxon example**	**Environment type**	**Closely related organism(s) (% sequence identity/% sequence coverage)**
HydA	Thermotogae/Dictyoglomi	*Thermotoga maritima/Dictyoglomus thermus*	Soil/surface water/hydrothermal vents/springs/groundwater	*Thermodesulfatator indicus* (49/97)/*Desulfotomaculum australicum* (53/96)
MvhA	Methanomicrobiales	*Methanobacterium* sp.	Hydrothermal vents/springs	*Acetomicrobium thermoterrenum* (57/94)
FdhA	δ-Proteobacteria	*Desulfobacula toluolica*	Surface water	*Desulfobacterium autotrophicum* (50/94)
HytA	Firmicutes	*Clostridium scatologenes*	NA	NA
NfnSL	Actinobacteria/ Proteobacteria	*Mobiluncus curtisii*/*Shewanella halifaxensis*	Surface water/hydrothermal vents/springs	*Carboxydothermus islandicus* (70/99)
FixAB	Crenarchaeota	*Aeropyrum camini*	Hydrothermal vents/springs	*Desulfotomaculum thermocisternum* (52/99)
Bcd	Firmicutes	*Eubacterium limosum*	Soil	*Caldisalinibacter kiritimatiensis* (70/99)
CarC	Firmicutes	*Natranaerobius thermophilus*	Deep subsurface/soil	*Sporobacter termitidis* (60/99)
HylA	Firmicutes	*Thermincola potens*	Saline/hydrothermal vents/springs	*Caloranaerobacter ferrireducens* (70/99)
Ldh	Synergistetes/ Thermotogae	*Thermovirga lienii*/*Pseudothermotoga lettingae*	Deep subsurface	*Anaerotruncus rubiinfantis* (100/97)/*Desulfotignum balticum* (58/99)
Hdr2A	Firmicutes	*Moorella thermoacetica*	NA	NA
MetF	δ-Proteobacteria	*Desulfobaca acetoxidans*	Surface sediments	*Candidatus* Latescibacteria (95/100)

*^a^The subunit or suite of subunits (i.e., concatenation of NfnSL and FixAB) of oxidoreductases subjected to phylogenetic analysis is shown for each Bf system. NA, not available.*

*The taxonomic rank and a representative with a homolog comprising the earliest evolving, well-supported lineages, are provided. Multiple taxa are shown when no single group was strongly supported. The percent sequence identity and percent sequence coverage of each early evolving homolog to the most closely related homolog from a complete genome sequence is provided. Names of abbreviated protein complexes are provided in **Table 1***.

Homologs of HylA exhibited a very narrow taxonomic distribution and were only identified in the bacterial phylum Firmicutes (**Figure 5**). HylA homologs formed a single monophyletic clade suggesting that Hyl evolved once during its evolution (Supplementary Figure S3) through recruitment of HylBC and FdhF2, enabling FBEB. HylA homologs were nested among lineages comprising homologs of both Bf and non-Bf HydA, making it difficult to discern whether the ancestor of Hyl was already capable of FBEB (i.e., BC subunits were in place) or whether these subunits were recruited. Nonetheless, the pairwise distances of all the subunits of Hyl (i.e., HylABC) showed strong, positive correlations suggested they have been under selection to co-evolve (Supplementary Figure S5). The weak correlation between pairwise distances of FdhF2 and HylABC suggests that FdhF2 may have been recruited independently of HylABC and multiple times during the evolution of Hyl, potentially representing the trigger to diversify from Bf Hyd or non-Bf HydA.

The earliest evolving Hyl is from an anaerobic thermophile [*Thermincola potens* (ADG81609)] belonging to the phylum Firmicutes (100% bootstrap support). The next branching lineage also comprised homologs from thermophilic Firmicutes. This suggests an origin for Hyl among an anaerobic ancestor of the Firmicutes in an anoxic high temperature environment (**Table 2**).

Like Hyl, Hyt also exhibited a limited taxonomic distribution (**Figure 5**) and homologs were only detected in the genomes of autotrophic and acetogenic *Clostridium* strains (**Table 2**), suggesting an origin in an anoxic environment. Phylogenetically, HytA homologs were confined to a single lineage nested among non-Bf, monomeric HydA homologs. This indicates that HytBCDE1E2 and FdhA were likely recruited to an ancestor of HytA thereby allowing for FBEB (Supplementary Figure S3). Mantel tests conducted on matrices describing the pairwise distances of the subunits that comprise Hyt (i.e., HytABCDE1E2 and FdhA) show positive correlations suggesting that all these subunits are under selection and have co-evolved (Supplementary Figure S6). Interestingly, the HytA lineage comprises additional non-Bf HydA homologs, and these branching differences are well-supported (i.e., >96% bootstrap support). Thus, like Hyd, additional subunits that were recruited to HytA and that allow for FBEB were apparently easily lost.

#### Mvh

Previous phylogenetic reconstructions of the large subunit of Mvh (i.e., MvhA) suggests that it is derived from a non-Bf [NiFe]-hydrogenases through recruitment of the Hdr complex (i.e., HdrABC) and other subunits (i.e., MvhGD) (Schut et al., 2013; Boyd et al., 2014), an assessment that is supported by our phylogenetic reconstruction (Supplementary Figure S7). Mantel tests conducted on matrices describing the pairwise distances of the subunits that comprise Mvh (i.e., MvhAGD and HdrABC) show strong positive correlations (Supplementary Figure S8), indicating that once these subunits were recruited they were maintained and co-evolved. Phylogenetic reconstruction of MvhA homologs revealed a single monophyletic clade that comprised both bacterial and archaeal homologs. The earliest evolving MvhA in this clade contained archaeal homologs from anaerobic methanogens (i.e., *Methanobacterium* and *Methanocella*) within the order Methanomicrobiales, with bacterial sequences nested among methanogen lineages. A second more recently evolved clade of MvhA was identified that comprised homologs from anaerobic members of the Chloroflexi and Proteobacteria, suggesting acquisition of MvhA via horizontal gene transfer from a methanogen (Supplementary Figure S7). This observation, coupled with the limited distribution of Mvh in archaeal and bacterial genomes at a taxonomic level (**Figure 5**), suggests that Mvh was not a property of the LUCA. Rather, Mvh likely emerged in an anaerobe after the divergence of Archaea and Bacteria from the LUCA through recruitment of several additional subunits, including HdrABC, to an existing [NiFe]-hydrogenase isoform.

#### Fdh

Bf Fdh homologs exhibit a narrow taxonomic distribution but were identified in both archaeal (Euryarchaeota) and bacterial (Proteobacteria) genomes. Phylogenetic reconstruction of the oxidoreductase subunit of Fdh (i.e., FdhA) suggests that Bf Fdh postdates non-Bf formate dehydrogenases (Supplementary Figure S9). This suggests that the subunits (i.e., FdhB and HdrABC) that form Bf Fdh were recruited to FdhA. The earliest branching putatively Bf FdhA lineages comprise homologs from anaerobic Bacteria [e.g., the δ-proteobacterium *D. autotrophicum* (ACN14546)] with high bootstrap support, suggesting that Bf Fdh likely emerged in a bacterium in an anoxic environment. Bf archaeal FdhA lineages are nested among, and are paraphyletic to, bacterial FdhA lineages. This suggests that FdhB/HdrABC may have been recruited to an ancestor of proteobacterial FdhA. Moreover, these observations suggest that once FdhB/HdrABC were recruited to function with FdhA as a Bf Fdh complex, the Fdh complex was then laterally transferred to Archaea. This interpretation is supported by strong positive correlations between the pairwise distances of the subunits of Fdh complex (i.e., HdrABC and FdhAB) (Supplementary Figure S10), indicating that once these subunits were recruited, they were largely maintained and co-evolved. We interpret the patchy taxonomic distribution of Bf Fdh (only identified among Euryarchaeota and Proteobacteria; **Figure 5**), the lack of monophyly among archaeal and bacterial Bf FdhA homologs, and the observation that euryarchaeote homologs are nested among bacterial FdhA homologs to indicate that Bf Fdh was unlikely to be a property of the LUCA.

#### Nfn

In the case of Nfn, all known homologs have been proposed to be capable of Bf electrons based on conserved motifs (Nguyen et al., 2017). Due to this observation, we assume that the ancestor of Nfn also bifurcated electrons. Moreover, paralogs of Nfn with similar activities but which lack one of the subunits (NfnS or NfnL) were not detected in genomes which precludes a detailed assessment of the nature of the first Nfn complexes. Nonetheless, a mantel test conducted on the pairwise distances of NfnS and NfnL show a strong positive correlation (Supplementary Figure S11), suggesting that NfnS and NfnL have been under selection and co-evolved.

Despite evidence presented here indicating that NfnSL have likely co-evolved, our phylogenetic reconstruction revealed polyphyly with regards to taxonomic domains, with several interspersed bacterial and archaeal lineages. This suggests several successful lateral gene transfers of *nfnSL* have taken place between Bacteria and Archaea and these events may have occurred after the divergence of Bacteria and Archaea from the LUCA. The earliest evolving well-supported Nfn lineage comprises a homolog from *Mobiluncus curtisii* (ADI67453) belonging to the phylum Actinobacteria (**Table 2** and Supplementary Figure S12). The next branching lineage comprised a homolog from a proteobacterium. This suggests a bacterial origin for Nfn with early diversification among an ancestor of Actinobacteria and possibly Proteobacteria. Consistent with our interpretation that Nfn originated after the divergence of Archaea and Bacteria from the LUCA, several studies have indicated that both Actinobacteria and Proteobacteria are recently evolved phyla (Forterre, 2015; Hug et al., 2016).

#### Fix, Car, and Bf-Bcd

Previous bioinformatics analyses conducted on Etf suggests that non-Bf Etf, Bf Car, and Bf Bcd predate the emergence of Bf Fix, an assessment that was based on high bootstrap support (Garcia Costas et al., 2017) and that is consistent with the analyses conducted here. This suggests that FixCX were recruited to function with EtfBA (i.e., FixAB). Once these subunits were recruited, they co-evolved as indicated by mantel tests conducted on the pairwise distances of FixABCX that reveal strong positive correlations (Supplementary Figure S13).

Phylogenetic reconstruction of FixAB reveals multiple monophyletic clades of archaeal and bacterial homologs that are paraphyletic, suggesting several lateral transfers of this gene between these domains (Garcia Costas et al., 2017). Early evolving Fix lineages were generally not well-supported and comprised members of the Crenarchaeota as well as the Proteobacteria (**Table 2**). Paraphyly among bacterial and archaeal Fix lineages, combined with data indicating that Fix is primarily encoded in the genomes of aerobes and facultative anaerobes (**Figure 3B**), suggests that Fix was not a property of the LUCA. Rather, these features suggest a more recent origin for this Bf complex and that this likely occurred after the advent of oxygenic photosynthesis and the increase of atmospheric O_2_ ∼ 2.4 Ga (as summarized in Lyons et al., 2014). This interpretation is consistent with a recent report suggesting that Fix emerged relatively recently to supply Fd^-^ for nitrogenase in aerobic and anoxygenic phototrophic taxa (Poudel et al., 2018).

Bf-Bcd and Car are closely related, but functionally distinct paralogs that were only detected in bacterial genomes (**Figure 5**), suggesting that they emerged after the divergence of Archaea and Bacteria from the LUCA. Phylogenetically, the catalytic subunits of Bf-Bcd and Car (i.e., Bcd and CarC, respectively (**Table 1**)] each form coherent lineages that are interspersed with putatively non-Bf lineages (Supplementary Figure S14). This suggests that recruitment of electron transfer flavoprotein protein subunits (i.e., EtfAB and CarDE, respectively) that allow FBEB in these lineages each took place in singular events, but that loss of these subunits was common during the evolution of each of these functional complexes. This interpretation is supported by results from mantel regressions of matrices showing the pairwise phylogenetic distances of Bf-Bcd (i.e., EtfAB and Bcd) and Car (i.e., CarCDE) subunits, all of which show strong positive correlations (Supplementary Figures S15, S16, respectively). This indicates that subunits that comprise Bf-Bcd and Car have been under selection and have co-evolved, with several instances of loss of genes coding for EtfAB and CarDE, respectively, during evolution.

The earliest evolving putatively Bcd (the catalytic subunit of Bf-Bcd) lineage is from *Eubacterium limosum* (ALU16222) within the phylum Firmicutes (100% bootstrap support), which is followed by several lineages comprising additional homologs from Firmicutes. These lineages are well-supported, suggesting that Bf-Bcd likely evolved first in an anaerobic member of the Firmicutes. Likewise, the earliest evolving putatively Bf CarC (the catalytic subunit of Car) homologs were identified in the genome of *Natranaerobius thermophilus* (ACB85453) within the phylum Firmicutes (100% bootstrap support) (Supplementary Figure S14), followed by several additional well-supported lineages comprising homologs from anaerobic Firmicutes. Early evolving Bch and CarC homologs that putatively associate with EtfAB and CarDE, respectively, and are therefore predicted to bifurcate, form sister lineages. This suggests that these enzyme complexes may have evolved by duplication and subsequent diversification of the catalytic subunit after EtfAB/CarDE homologs had been recruited. Collectively, these results suggest that both Bf-Bcd and Car likely evolved in an anaerobic ancestor of the Firmicutes, possibly through duplication of genes, and that this event(s) postdated the divergence of Archaea and Bacteria from the LUCA.

#### Bf-Ldh

Homologs of Ldh (the catalytic subunit of Bf-Ldh) were only detected in bacterial taxa (**Figure 5**) suggesting that Bf-Ldh also evolved after the divergence of Archaea and Bacteria from the LUCA (Supplementary Figure S17). Moreover, Ldh that are predicted to associate with EtfAB based on gene context and thus are predicted to bifurcate are nested among putatively non-Bf Ldh (not associated with EtfAB) suggesting that EtfAB were recruited to Ldh allowing for FBEB. Three distinct lineages of Bf-Ldh were identified, and these were interspersed among non-Bf homologs. Mantel regressions conducted on matrices describing the pairwise phylogenetic distances of subunits that comprise Bf-Ldh (i.e., EtfAB and Ldh) show positive correlations (Supplementary Figure S18). This suggests that the multiple lineages of Bf-Ldh likely have a common origin and that the non-Bf lineages that intersperse lineages of putatively Bf-Ldh result from gene loss.

The earliest evolving Bf-Ldh lineage comprised a homolog from the genome of the anaerobic, moderate thermophile *Thermovirga lienii* (AER67312) within the phylum Synergistetes (100% bootstrap support). The next branching lineage comprised homologs from genomes affiliated with the Thermotogae. Collectively, these observations suggest that Bf-Ldh evolved in an anaerobic ancestor of the Synergistetes or Thermotogae after the divergence of Archaea and Bacteria from the LUCA (Supplementary Figure S17).

#### Hdr2

HdrABC homologs were identified in both archaeal and bacterial genomes that were unaccounted for by Mvh, Fdh, and Met, and therefore were denoted as Hdr2. However, it is not possible to definitively determine which HdrABC homologs associate with Mvh, Fdh, or Met, unless HdrABC homologs are identified in genomes that do not encode Mvh, Fdh, or Met, which was uncommon. This in turn, makes it impossible to evaluate the evolutionary history of HdrA as it relates to bifurcation. However, regardless of whether HydrABC homologs can be definitively shown to form a complex with Bf complexes (i.e., Mvh, Fdh, Met, and Hdr2), we can evaluate the evolutionary history of HdrA to determine whether HdrABC were likely to be a property of the LUCA.

Early evolving homologs of HdrA were from bacterial genomes that belonged to the phyla Proteobacteria (e.g., *Desulfobacca acetoxidans*) and Firmicutes (e.g., *M. thermoacetica*) (Supplementary Figure S19). Archaeal HdrA homologs were nested among bacterial HdrA homologs suggesting that Archaea acquired HdrA through horizontal gene transfer from Bacteria. These observations suggest that HdrA is not a property of the LUCA.

#### Met

A narrow taxonomic distribution of Bf Met homologs was detected among both archaeal (Euryarchaeota) and bacterial (Firmicutes) taxa. Phylogenetic reconstruction of homologs of MetF suggests that Bf Met postdates non-Bf methylene-H_4_F reductases (Supplementary Figure S20). This suggests that the subunits that comprise the Met complex (MetV, MvhD, and HdrABC) were recruited to MetF to enable FBEB. Two distinct lineages of putatively Bf MetF were identified, and these were interspersed by non-Bf homologs. Mantel regressions conducted on matrices describing the pairwise phylogenetic distances of subunits that comprise Met (i.e., MetFV, MvhD, and HdrABC) show positive correlations (Supplementary Figure S21). This suggests that once the subunits were recruited they were under selection and have co-evolved.

The earliest evolving lineage of Met comprised only one homolog from the anaerobe *D. acetoxidans* (AEB08698) that belongs to the phylum Proteobacteria, with high bootstrap support. This suggests that Met likely evolved in an anaerobic ancestor of Proteobacteria. Only one archaeal Met homolog was identified, and this was encoded in the genome of the methanogen *M. intestinalis*. Phylogenetic reconstruction of MetF from this putative Bf complex reveals that it is nested among bacterial MetF homologs suggesting that it was likely acquired via horizontal gene transfer from Bacteria. Collectively, our findings suggest that Met was not a property of the LUCA but rather emerged in a bacterial anaerobe and was laterally transferred to Archaea.

### Environmental Distribution and Phylogenetic Ecology of Electron Bf Enzyme Homologs

Results from our screening and analysis of complete genome sequences reveal that homologs of Bf enzymes are enriched in anaerobes and strongly suggest that each enzyme system has an independent origin in anaerobic taxa, with the potential exception being Fix. We therefore hypothesized that microbial communities inhabiting anoxic environments would (i) be enriched in homologs of Bf enzymes relative to oxic environments and (ii) host the earliest evolving homologs of enzymes involved in FBEB. Specifically, we hypothesized that subsurface environments, which are characteristically limited in redox gradients and feature a low flux of oxidants and other nutrients (Edwards et al., 2012; Colman et al., 2017), would select for organisms reliant on anaerobic metabolism and Bf enzymes and may have promoted the origin of Bf enzyme complexes.

To test these hypotheses, we compiled 3,136 available metagenomic sequences and classified them as being from ‘surface’ or ‘subsurface’ environments based on a previously described classification scheme (Supplementary Table S3; Colman et al., 2017). Briefly, metagenomes were classified as surface or subsurface based on available metadata associated with the metagenome, where subsurface environments were defined as the following environments: groundwater, deep subsurface, hydrothermal vents/springs, subsurface sediments, or marine sediments > 1 m below sea floor. Surface environments included all others that did not fit the above classification, including saline environments, surface waters, and soils. We then screened these metagenomes for homologs of the 11 characterized Bf enzymes and subjected the catalytic oxidoreductase subunits to informatics analysis and phylogenetic reconstruction (metagenomes were not screened for Hdr2; see section “Materials and Methods”).

Screening of the 3,136 metagenomes revealed that 893 metagenomes coded for a homolog of at least one Bf enzyme (Supplementary Table S3 and Supplementary Files S1–S10). We identified homologs of nearly all 11 Bf enzymes in at least one metagenome except for homologs of the heptameric NADP(H)-dependent formate dehydrogenase (Hyt) complex. The lack of Hyt detection may be due to the stringent requirements that we imposed for identification of a Hyt complex (all six genes must be present in near synteny to be considered a Hyt homolog). However, while the number of subunits for a Bf enzyme (2–7 subunits) and the abundance of those enzyme homologs in metagenome sequences was inversely correlated, the relationship was not significant (*R*^2^ = 0.18) (data not shown). This suggests that the lack of detection of homologs with more subunits was not due simply to the lower probability of assembling contigs with the minimal number of specified genes in near synteny.

In general, the abundance of Bf homologs identified in metagenomes (Supplementary Figure S22A) reflected the distribution identified in complete genomes (Supplementary Table S1), with a few important exceptions. Mvh, and Met were enriched in the metagenomic dataset when compared to the genomic dataset. These enzyme complexes are all either involved in alkane-related metabolisms such as methanogenesis/methanotrophy, sulfate reduction, acetogenesis, or other potentially diverse anaerobic metabolisms (Buan and Metcalf, 2010; Kaster et al., 2011; Buckel and Thauer, 2013, 2018a,b; Mock et al., 2014; Yan et al., 2017). Conversely, Bf-Bcd and Fix were enriched in the complete genome dataset when compared to the metagenomic dataset. Bf-Bcd is involved in fermentation (Djordjevic et al., 1995; Li et al., 2008; Chowdhury et al., 2014) and is predominantly identified among members of the Firmicutes and Bacteroidetes (Supplementary Table S1) whereas Fix is involved in nitrogen fixation (Earl et al., 1987; Edgren and Nordlund, 2004; Ledbetter et al., 2017) within the Proteobacteria and Firmicutes, although homologs predicted to bifurcate were identified in other non-diazotrophic taxa (Supplementary Table S1).

Differences in enrichment of Bf enzyme homologs in genomic versus metagenomic datasets might be explained by differences in the coverage of certain metabolic guilds in whole-genome databases (generally derived from cultivars) when compared to metagenomic datasets. For example, several taxonomic groups of methanogens or anaerobic alkane degraders have only recently been discovered or characterized via environmental genomics surveys (Paul et al., 2012; Evans et al., 2015; Laso-Perez et al., 2016; Vanwonterghem et al., 2016; Sorokin et al., 2017), suggesting that they exhibit much greater diversity than is currently represented in genomic databases from cultivars. This could account for the relative enrichment of Mvh in metagenomic datasets. In contrast, Bf-Bcd and Fix may be overrepresented in the whole-genome dataset due to overrepresentation of cultivated fermenters that tend to encode Bf-Bcd (Firmicutes and Bacteroidetes phyla) and diazotrophic Proteobacteria that tend to encode Fix in whole-genome datasets (Rinke et al., 2013).

**FIGURE 6:**
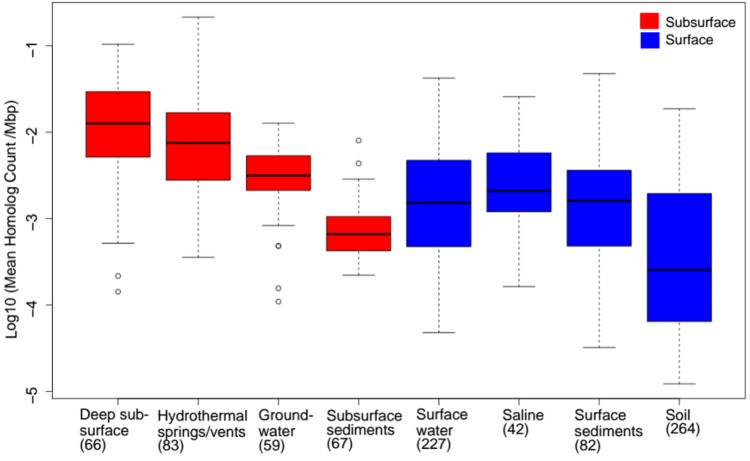
Abundance of homologs of the 11 (see **Table 1**) Bf enzyme complexes in metagenomic sequences (Hyt homologs were not identified in metagenomes and therefore are not included for this analysis). Here, the abundances of homologs of all Bf enzyme complexes identified among non-redundant metagenomic contigs were normalized to the total number of megabase pairs (Mbp) of sequence in those contigs to account for differences in sequence depth between datasets. Metagenomes were classified into different environmental groups based on metadata provided for each dataset (see Supplementary Table S3). The number in parentheses next to the environmental groups, as specified on the *x*-axis, represents the total number of metagenomes classified into a given environmental group. Only environmental (e.g., non-engineered and non-host-associated) metagenomes were used in the analyses.

Of the 893 metagenomes that encode Bf enzyme homologs, 275 were classified as being derived from subsurface-like environments, and 618 were classified as being derived from surface-associated environments (Supplementary Table S3). Metagenomes with Bf enzyme homologs were further classified into eight sub-categories (**Figure 6**), with most being derived from surface soils (30% of the total metagenomes) and surface waters (25% of the total metagenomes) that included lakes, freshwater streams, and oceans (Supplementary Table S3). When metagenomes from the subcategories of subsurface environments or surface environments were considered together, those from subsurface locales harbor a significantly greater abundance of Bf enzyme homologs per mega base pair (Mbp) of assembled sequence than those from surface environments (Welch’s two sample *t*-test, *P* < 2.2 × 10^-16^) (Supplementary Figure S22B). This is consistent with our hypothesis that the capacity to bifurcate electrons should be enriched in subsurface-like environments that generally exhibit minimal redox gradients, highly reducing conditions, and low nutrient fluxes (Edwards et al., 2012; Kieft, 2016; Colman et al., 2017). In particular, homologs of the ten Bf enzymes (Hyt was not detected and Hdr2 was not considered), when considered together, were significantly enriched in deep subsurface rocks and fracture fluids as well as hydrothermal springs/vents (**Figure 6**), but were surprisingly not enriched in subsurface sediment microbiomes relative to those from surface environments.

**FIGURE 7:**
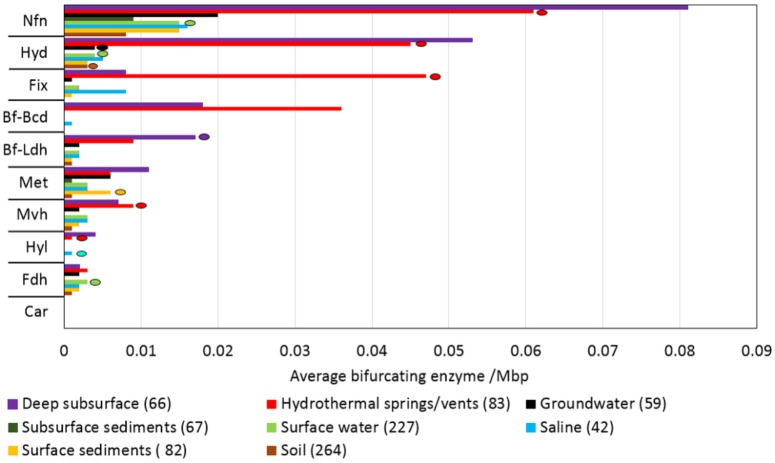
Average abundance of homologs of the ten (see **Table 1**) Bf enzyme complexes within metagenomes (*n* = 928 metagenomes). Hyt homologs were not identified in metagenomes and therefore are not depicted in the graph. Hdr homologs are difficult to demarcate from the ones that form a complex with Mvh, Fdh, or Met so were not included in the metagenome analysis. The average abundances of homologs of all Bf enzyme complexes in non-redundant metagenomic contigs were normalized to the total number of megabase pairs (Mbp) of sequence in those metagenomes to account for differences in sequence depth between metagenomes. The colored circle in the graph represents the environment (colored accordingly) that contained the early evolving homologs of specific Bf enzymes (also included in **Table 2**). Only a small number of Bf-Bcd and Car homologs were identified in metagenomes and thus, resolving the environment of their presumed origin was deemed not appropriate from their phylogenies. Metagenomes were classified using metadata provided for each dataset. The total number of metagenomes classified into each environmental group is given in parentheses. Names for each abbreviated protein complex are provided in **Table 1**.

We further scrutinized the distribution of Bf enzyme homologs among more specific environmental types (**Figure 7**). Most of the Bf complexes were identified in at least seven of the eight metagenome subcategories, with the exception of Hyt (not detected), Hdr2 (not included) and Car, the latter of which was only identified in one deep subsurface and one soil metagenome. Homologs of Bf enzyme complexes were generally overrepresented in metagenomes associated with deep subsurface habitats, such as subsurface rock fracture fluids and were overrepresented in environments that share characteristics with subsurface environments, such as hydrothermal springs/vents (**Figure 7**). This was particularly true for Nfn, Hyd, Fix, and Bf-Bcd. Like deep subsurface environments, hydrothermal springs/vents often exhibit highly reducing conditions and minimal energetic gradients (Jannasch and Mottl, 1985; Amend and Shock, 2001; Martin et al., 2008; Shock et al., 2010; Martin, 2012; Amenabar et al., 2015). Consequently, the capacity to bifurcate electrons in these environments is likely ecologically advantageous, allowing anaerobic or facultatively anaerobic microorganisms to leverage minimal energetic gradients to support efficient metabolisms.

To assess whether subsurface-like environments promoted the origin of Bf enzymes complexes, we investigated the phylogenetic distribution of Bf homologs. We reconstructed phylogenies of homologs of catalytic subunits of each of the ten complexes identified in the metagenomic data, mapped the environment types to the phylogenies, and then determined the environment types that are associated with the earliest branching homologs for each complex. These analyses indicated that most (7 of the 10 enzyme complexes that were detected/considered) of the Bf complexes featured deep-branching lineages comprising homologs from either deep subsurface or hydrothermal springs/vents metagenomes (**Figure 7**, **Table 2** and Supplementary Figures S23–S30), although other environmental origins could not be summarily excluded for several of these enzymes.

Deep subsurface or hydrothermal spring/vent environments host early evolving lineages of the catalytic subunits of Nfn, Hyd, Fix, Mvh, Bf-Ldh, Hyl, and Car. The three enzymes (includes Met, Fdh, and Bf-Bcd) where phylogenetic data potentially suggest an origin in a surface like environment are nonetheless found almost exclusively in the genomes of anaerobes (**Figure 3B**). Many surface environments considered to be broadly oxidized (e.g., soils, freshwaters, saline microbial mats) harbor anoxic microcompartments (Stein et al., 2001; Shen et al., 2016) that may support anaerobic taxa that encode Bf enzyme homologs, potentially helping to explain this observation. In addition, high rates of microbial respiration have been observed in many surface environments such as in marine sediments of the ocean margins where oxygen (O_2_) penetrates only millimeters to centimeters (D’Hondt et al., 2015). Thus it is not unusual to find anaerobic microorganisms that may encode Bf enzyme homologs in environments that were classified as surface environments.

The taxonomic identities of the earliest branching homologs of Bf enzymes from metagenomes broadly agree with our analyses of complete genomes (**Table 2**). Specifically, both analyses indicate that several of the Bf enzymes are likely to have originated in thermophiles, sulfate reducers, and members of the Firmicutes. Thermophiles were well-represented in the earliest branching homologs of Bf hydrogenases (Hyd and Mvh), Nfn, Fix, and Hyl. Origins for Bf enzymes in thermophiles is consistent with the generalized lack of O_2_ in high temperature environments due to the inverse relationship between O_2_ solubility and temperature (Amend and Shock, 2001) and to the limited availability of high potential oxidants in these environments (Shock et al., 2010). These characteristics would select for anaerobic taxa with metabolisms such as methanogenesis, acetogenesis, fermentation, and sulfur/sulfate reduction and for adaptations that maximize the efficiency of these metabolisms, such as FBEB (Buckel and Thauer, 2018a,b).

For several classes of Bf enzymes, the deepest-branching lineages of homologs were phylogenetically divergent from those that are currently represented in our complete genome database (**Table 2**). It is possible that the function of these Bf homologs may differ from those that have been biochemically characterized. For example, the deepest-branching [NiFe]-hydrogenase Mvh homolog was identified in a deep sea hydrothermal vent community and is distantly related to Mvh from *Acetomicrobium thermoterreneum* (**Table 2**). To date, Bf Mvh complexes have only been characterized in Archaea (Kaster et al., 2011), where they function to reduce the disulfide bond in CoM-CoB during methanogenesis (Thauer et al., 2008; Buckel and Thauer, 2013). The bacterium harboring this Bf Mvh-like complex almost certainly is not a methanogen, since methanogenesis has not been observed outside of Archaea (Vanwonterghem et al., 2016; Sorokin et al., 2017). Importantly, in addition to this deeply rooted Mvh homolog from a putative hydrothermal vent bacterium, homologs of Mvh were identified in the genomes of other non-methanogenic taxa, including those that catalyze sulfate, sulfur, and nitrate reduction (Supplementary Figure S1). This may suggest a unique functional role for Mvh in these non-methanogenic taxa.

With few exceptions, the amino acid sequence identities of earliest branching homologs for the ten Bf enzymes identified/considered in our metagenomic sequence dataset to the nearest characterized cultivar in our genome dataset were low (**Table 2**). The lack of close representation in existing genomic databases or culture collections for many of these early evolving homologs from metagenomic datasets suggests the presence of a greater diversity of Bf enzyme complexes in natural systems than is currently available in genome databases from cultivars. To assess the diversity of Bf enzyme homologs in genome and metagenomic datasets and to determine the extent that the diversity present in metagenomic datasets is captured by genomic datasets, we calculated the pairwise sequence identities of each pair of homologous sequences within each of the ten Bf enzymes that were detected/considered in our metagenomic sequence analysis. Specifically, we conducted pairwise sequence comparisons of (1) metagenome versus complete genome homologs and (2) complete genome versus complete genome homologs. We quantified an E-value (see section “Materials and Methods”) as a proxy for protein identity distance, where higher values indicate that average pairwise identities among sequences are higher and that they are thus less diverse on average.

**FIGURE 8:**
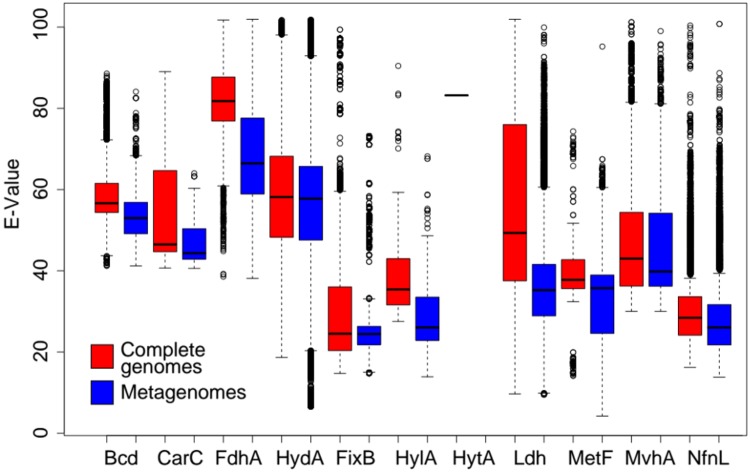
Distribution of E-values of homologs of the catalytic oxidoreductase subunit of the 11 (see **Table 1**; Hyt was not identified in our metagenomic sequence database) Bf enzyme complexes identified in metagenomes (in red) and complete genomes (in blue) against those of the complete genomes. Homologs identified in genome sequences were subjected to pairwise phmmer analysis to obtain E-values as an indication of their diversity. Similarly, homologs identified in metagenome sequences were subjected to phmmer analysis against a database comprising homologs obtained from genomes to assess the extent of the diversity of sequences in taxa that are either yet to be cultivated or that lack complete genome sequences. To simplify data presentation, the E-values were then normalized by multiplying by –10000 which is depicted as ‘E-Value’ on the *y*-axis. Higher distributions of E-Values indicate that homologs exhibit a lower diversity and thus are more similar phylogenetically. Lower distributions of E-Values indicate that homologs are more diverse and are less similar phylogenetically. The box represents the interquartile range with the whiskers showing the full range of the data. The circles outside of those whiskers represent outliers and the horizontal black bold line within the box represents the median value. Homologs identified in metagenomes that exhibit a lower average or range of E-values than those identified in complete genomes are underrepresented in complete genome databases (i.e., there is unsampled diversity).

Calculation of E-values for homologs indicates that those identified in complete genomes have a relatively narrow distribution of amino acid identities when compared to the homologs identified in the metagenomes (**Figure 8**). In particular, the significantly lower median E-values for Bcd, FdhA, HylA, Ldh, and MetF homologs in metagenomic sequences when compared to those from complete genome sequences indicates that a greater diversity of Bf enzymes exists in natural environments than currently exists in genomic databases from cultivars. Although the median E-values for the remaining Bf enzymes were not significantly different between the datasets, they were all comparatively lower in the metagenomes than in the complete genomes and many metagenomes comprised unique outlier homologs (e.g., HydA), suggesting the presence of novel protein sequences. Collectively, these results suggest that there is a far greater diversity of uncharacterized homologs of Bf enzymes in natural environments that may harbor unique functional properties that are distinct from those homologs that have been biochemically characterized.

## Conclusion

Complete and publicly available archaeal, bacterial, and eukaryal genomes were screened for homologs of the 12 enzyme complexes that have been biochemically shown to bifurcate electrons. Homologs of putative Bf enzymes were identified in organisms with diverse metabolisms, including those of aerobes and anaerobes, chemotrophs and phototrophs, and autotrophs and heterotrophs. Homologs were identified in both archaeal and bacterial taxa, however, they were heavily concentrated in the genomes of anaerobic taxa and were enriched among members of the Euryarchaeota, Firmicutes, Bacteroidetes, and Proteobacteria. The limited taxonomic distribution of homologs of the 12 Bf complexes, the observed paraphyly of archaeal and bacterial homologs of catalytic subunits of 11 of these 12 complexes (Hdr2 not evaluated), and inferred origins of homologs among different taxonomic domains suggests that the ability to bifurcate electrons evolved after the divergence of Archaea and Bacteria from the LUCA and that these enzyme complexes evolved independently multiple times. This suggests an ecological advantage to integrate FBEB into the energy metabolism of functionally distinct and phylogenetically diverse taxa, specifically those that are anaerobic. Importantly, interspersed lineages of Bf and non-Bf homologs in phylogenetic reconstructions and strong evidence for co-evolution of protein subunits indicates that loss of flavoproteins and other subunits was common during the evolution of the oxidoreductase subunits.

Interestingly, our conclusion that homologs of the 12 currently known Bf enzymes were not likely a property of the LUCA indicates that extant Bf enzyme complexes are not primordial and contrasts with conclusions from previous reports on the natural history of FBEB (Baymann et al., 2018) and the origins of FBEB (Martin, 2012, Martin et al., 2017; Baymann et al., 2018; Sousa et al., 2018). These studies based their conclusions on apparent structural conservation within three groups of flavoproteins that form complexes with oxidoreductases that enable FBEB. In at least one of these flavoprotein groups (i.e., the Etf group that comprises four of the 12 Bf enzyme families), phylogenetic approaches suggest that flavoproteins that associate with Bf enzymes are derived from non-Bf enzymes (Garcia Costas et al., 2017). This indicates that the Bf EtfB flavoproteins are recently evolved and is consistent with our conclusions based on the taxonomic distribution of these complexes and the evolutionary history of their oxidoreductase subunits. While the evolutionary histories of the other two structural groups of flavoproteins have not yet been fully resolved, the combination of the taxonomic distributions of the enzyme complexes and evolutionary analyses of their oxidoreductase subunits compiled here leads us to conclude that the Bf flavoproteins in the remaining two groups are also unlikely to be primordial. Regardless of whether the Bf flavoproteins themselves are primordial, the current configuration of these Bf flavoproteins and oxidoreductases are unlikely to be reflective of primordial complexes, based on the evolutionary analyses conducted herein.

Our analysis of the distribution and composition of Bf enzyme homologs in metagenomic sequences revealed enrichment of homologs in subsurface environments relative to surface environments. This is particularly true for deep subsurface rock fracture fluids and thermal spring/hydrothermal vent environments, where homologs of Bf enzymes were shown to be highly enriched. Moreover, phylogenetic reconstructions of the catalytic subunits of Bf enzyme complexes identified in metagenomic sequences revealed that the earliest evolving lineages were often (7 out of 10 instances; Hyt not identified in metagenomic datasets, Hdr2 not assessed) from deep subsurface rock fracture fluid and thermal spring/hydrothermal vent communities. This suggests that the characteristics of these environments, that at one time promoted the origin of these Bf complexes, may also help to explain the prevalence of Bf enzyme homologs in these environments today. The specific characteristics of these environments that may have promoted the origins of several of the Bf enzyme classes, and that promote the establishment of contemporary communities enriched in these protein complements, includes minimal redox gradients, highly reducing conditions, and low nutrient fluxes (Edwards et al., 2012; Kieft, 2016; Colman et al., 2017). Many of the homologs of Bf enzymes recovered from these environmental genomes, including those that branch early in phylogenetic reconstructions, exhibit low sequence identities with homologs available in complete genome databases. This suggests the existence of novel and diverse Bf homologs in natural environments that could be prioritized for characterization. Moreover, the apparent ease by which phylogenetically distinct flavoproteins can be recruited to function with oxidoreductases (12 independent origins of Bf enzymes, as revealed here) suggests the possibility of numerous other yet to be discovered Bf systems in both genomic and metagenomic sequence datasets.

The observations made here suggest that FBEB is a widely distributed strategy in organisms inhabiting anoxic environments where the energy yield of redox reactions is generally low and where increased efficiency of energy capture would be highly selectable (Peters et al., 2016, 2018; Buckel and Thauer, 2018a,b). The prevalence of homologs of putatively Bf enzymes in the genomes of anaerobes and in subsurface environments that are presumably anoxic is consistent with the hypothesized role of FBEB in supporting the energy metabolism of anaerobic life on early Earth (Martin, 2012; Sousa et al., 2018), when O_2_ concentrations were low (Lyons et al., 2014) and energetic gradients capable of supporting metabolism are thought to have been minimal (Schut et al., 2016). Phylogenetic data indicates that most Bf enzyme systems evolved from putatively non-Bf ancestors. Thus, as biology evolved new metabolic strategies allowing it to diversify into new ecological niches, many existing oxidoreductases were fine-tuned through recruitment of flavoproteins allowing FBEB. This in turn, would have allowed for coupling of new oxidation-reduction reactions involving a variety of substrates in cells that function to increase the metabolic efficiency and thus competitiveness of those cells. The near complete absence of Bf enzyme homologs in aerobic cells (exception being Fix) suggests that as biology diversified from anoxic to oxic environments, the selective advantage for Bf systems decreased, presumably due to toxicity of O_2_ to the cells or the sensitivity of the enzymes themselves to O_2_. In the case of Fix, it appears that aerobic diazotrophic cells specifically acquired and maintained this enzyme complex as an adaptation to generate Fd^-^ for use in supporting nitrogenase activity (Poudel et al., 2018), which evolved first in an anaerobe and later diversified into aerobes (Boyd et al., 2011a,b, 2015; Boyd and Peters, 2013).

Significantly more work needs to be completed to identify the specific triggers that led to the emergence and extinction of Bf systems as biology diversified to occupy the breadth of niche space as it does today. Moreover, additional work needs to be conducted to identify the unique environmental characteristics that selected for the emergence of Bf systems. Such studies will shed new light on the evolutionary history of FBEB and provide new perspective on the physiological and ecological role of Bf enzymes in these cells and the natural environment. The insights and perspectives obtained from conductance of these evolutionary ecology studies, framed within a robust understanding of the physiology and biochemistry of these enzymes, will provide a plethora of exciting new questions to guide future thinking and research into the nature of microbial life on early Earth and the role of FBEB in the physiology and ecology of that life. Insights from such studies will ultimately be required to evaluate the merits of placing the process of FBEB as a property of the LUCA, as has been suggested elsewhere (Martin, 2012; Martin et al., 2017; Baymann et al., 2018; Sousa et al., 2018), and the merits of placing the emergence of FBEB after the divergence of Bacteria and Archaea from the LUCA, as suggested here. If it turns out that FBEB was not a property of the LUCA, as advocated here, new challenging questions will emerge such as how to reconcile the energy metabolism of cells whose contemporary energetics are dependent on FBEB. In the case of hydrogenotrophic methanogens, which are often advocated as operating energy metabolisms reminiscent of that which operated in the LUCA (Weiss et al., 2016), it would be necessary to envision a primitive energy metabolism within a specific environmental context that favored reaction energetics that were not dependent on FBEB (Thauer et al., 2008). Similar scenarios would need to be envisioned for other fermentative and autotrophic cells whose contemporary energy metabolisms are also dependent on FBEB.

## Author Contributions

SP and EB designed the study and wrote the paper. SP conducted informatics analyses. SP, ED, ML, MA, EF, DC, and EB downloaded the metagenomes, provided feedback, and contributed to the writing of the manuscript.

## Conflict of Interest Statement

The authors declare that the research was conducted in the absence of any commercial or financial relationships that could be construed as a potential conflict of interest.
