# Supplementary material for: Origin and Evolution of Flavin-Based Electron Bifurcating Enzymes
Source: Front Microbiol. 2018 Aug 3;9:1762. doi: 10.3389/fmicb.2018.01762 (PMC6085437; doi:10.3389/fmicb.2018.01762)
Supplement: Supplementary file 14 [file Image_1.PDF]

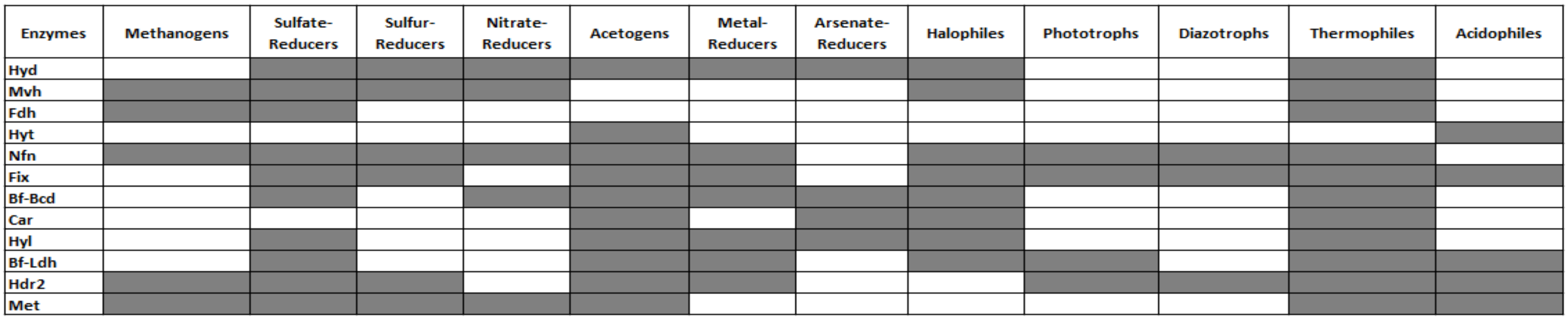

Supplementary Figure 1. Presence (grey) or absence (white) of homologs of one or more of the twelve bifurcating enzyme complexes encoded in the genomes of organisms with specified physiological attributes.

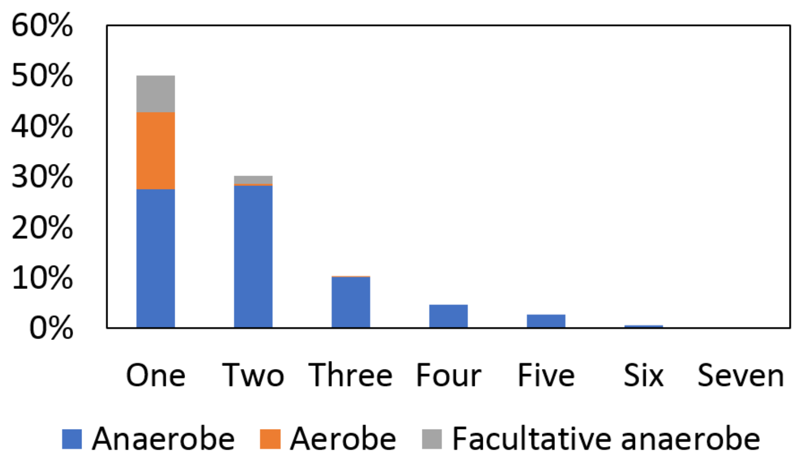

Supplementary Figure 2. Histogram depicting the percent of complete genome sequences that encode homologs of one to seven Bf enzyme complexes, out of 681 genomes found to contain at least one Bf enzyme complex. The bar chart also shows the distribution of these genomes as they relate to the ability of the host organism to integrate oxygen into their energy metabolism.

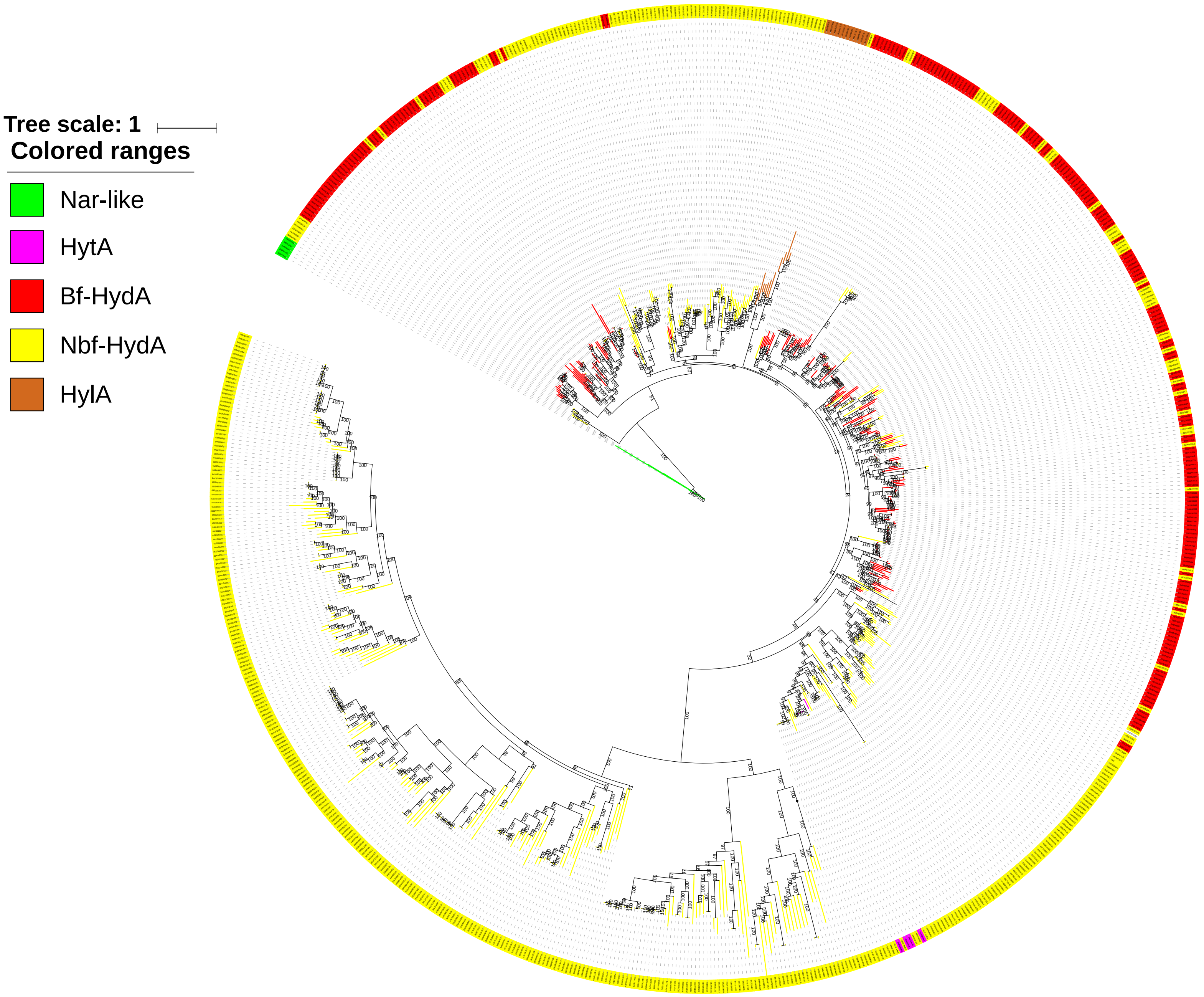

Supplementary Figure 3. Maximum-likelihood phylogenetic reconstruction of Bf [(Bf)-HydA, HytA, and HylA] and non-Bf HydA homologs [(Nbf)-HydA] and Nar-like proteins in complete genome sequences. The phylogeny was rooted with Nar-like proteins from *Homo sapiens* (NP_071938 and NP_036468), *Danio rerio* (A2RRV9), *Thalassiosira pseudonana* (XP_002289272) and *Ostreococcus lucimarinus* (XP_001416706). Sequence terminals are color coded, with Bf-HydA in red, HytA in purple, HylA in rust, Nbf-HydA in yellow, and Nar-like homologs in green. Names for each abbreviated protein complexes are provided in **Table 1**. Note, homologs of Bf-HydA, HytA, HylA were only identified among Bacteria, thereby negating the need to add a colored strip demarcating archaeal and bacterial homologs. Bootstrap values for each node are shown as a percentage (out of 1000 bootstrap replicates).

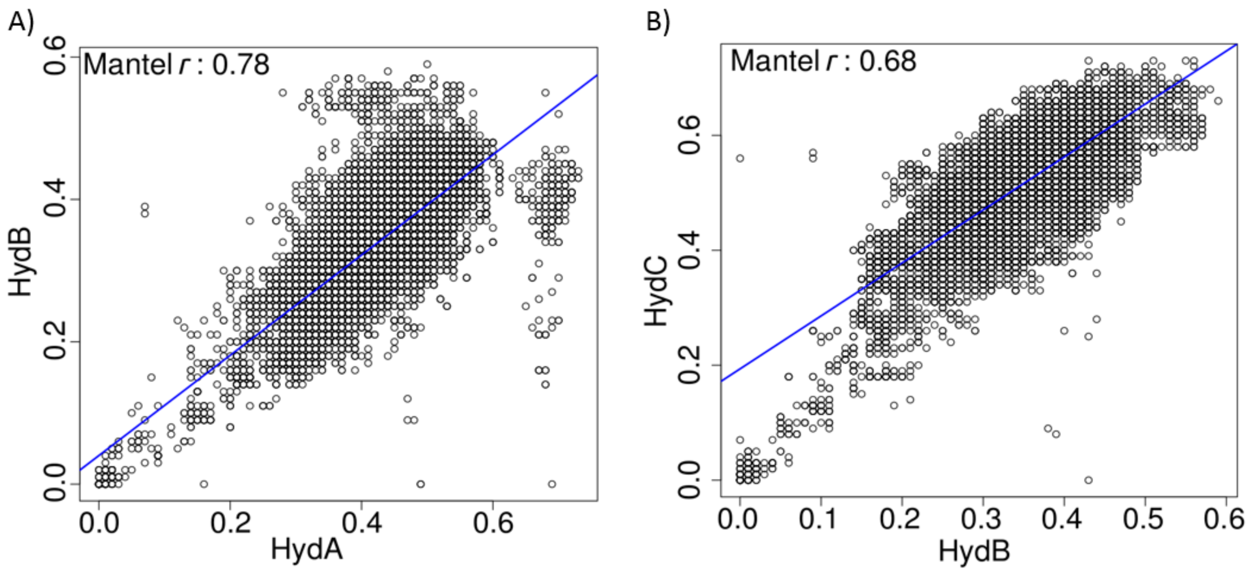

Supplementary Figure 4. Mantel regression of the pairwise distances of homologs of subunits of the Hyd complex plotted as a function of each other: **A**) HydA and HydB, **B**) HydB and HydC. Mantel *r* values are provided and the significance values (*p*-value) for both regressions were <0.001. Names for each abbreviated protein complex and their subunits are provided in **Table 1**.

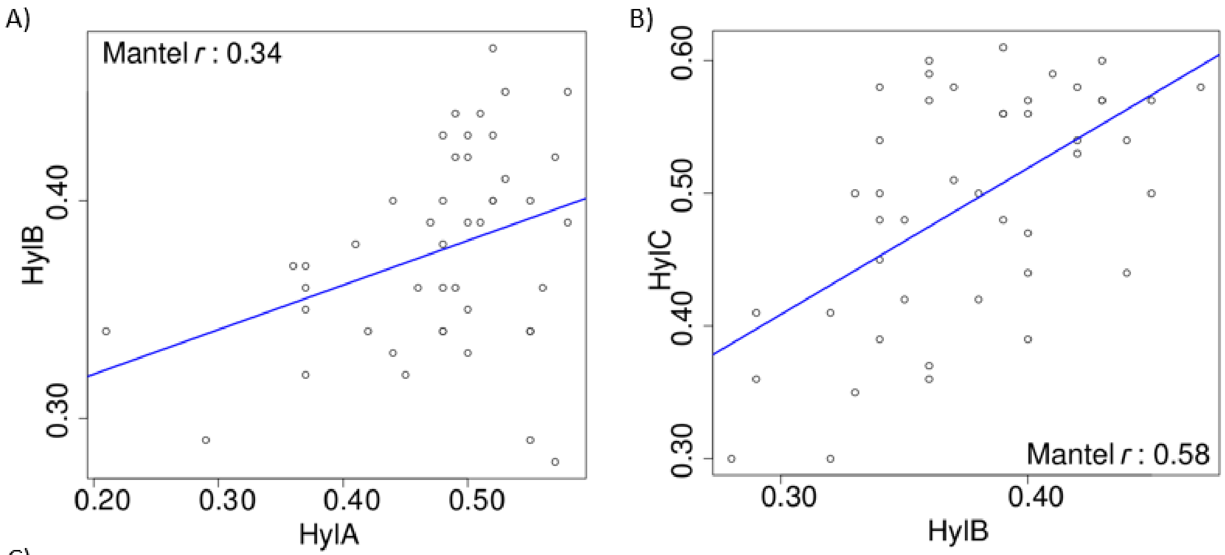

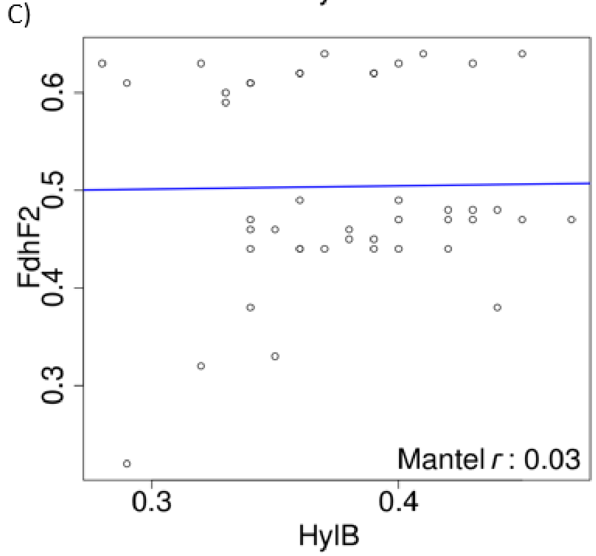

Supplementary Figure 5. Mantel regression of the pairwise distances of homologs of subunits of the Hyl complex plotted as a function of each other: **A**) HylA and HylB, **B**) HylB and HylC and **C**) HylB and FdhF2. Mantel *r* values are provided and the significance values (*p*-value) for all regressions were <0.02. Names for each abbreviated protein complex and their subunits are provided in **Table 1**

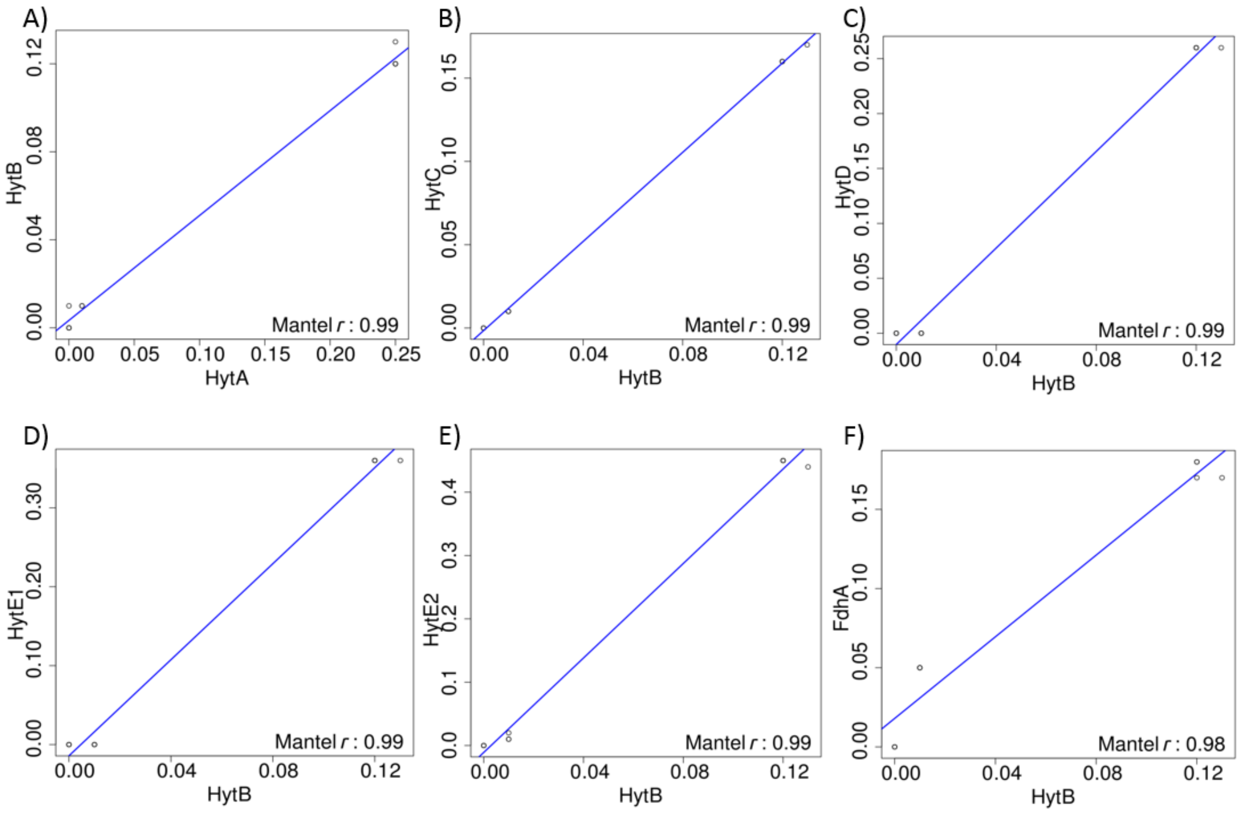

Supplementary Figure 6. Mantel regression of the pairwise distances of homologs of subunits of the Hyt complex plotted as a function of each other: **A**) HytA and HytB, **B**) HytB and HytC, **C**) HytB and HytD, **D**) HytE1 and HytB, **E**) HytE2 and HytB, and **F**) HytB and FdhA. Mantel *r* values are provided and the significance values (*p*-value) for all regressions shown were <0.2. Names for each abbreviated protein complex and their subunits are provided in **Table 1.**

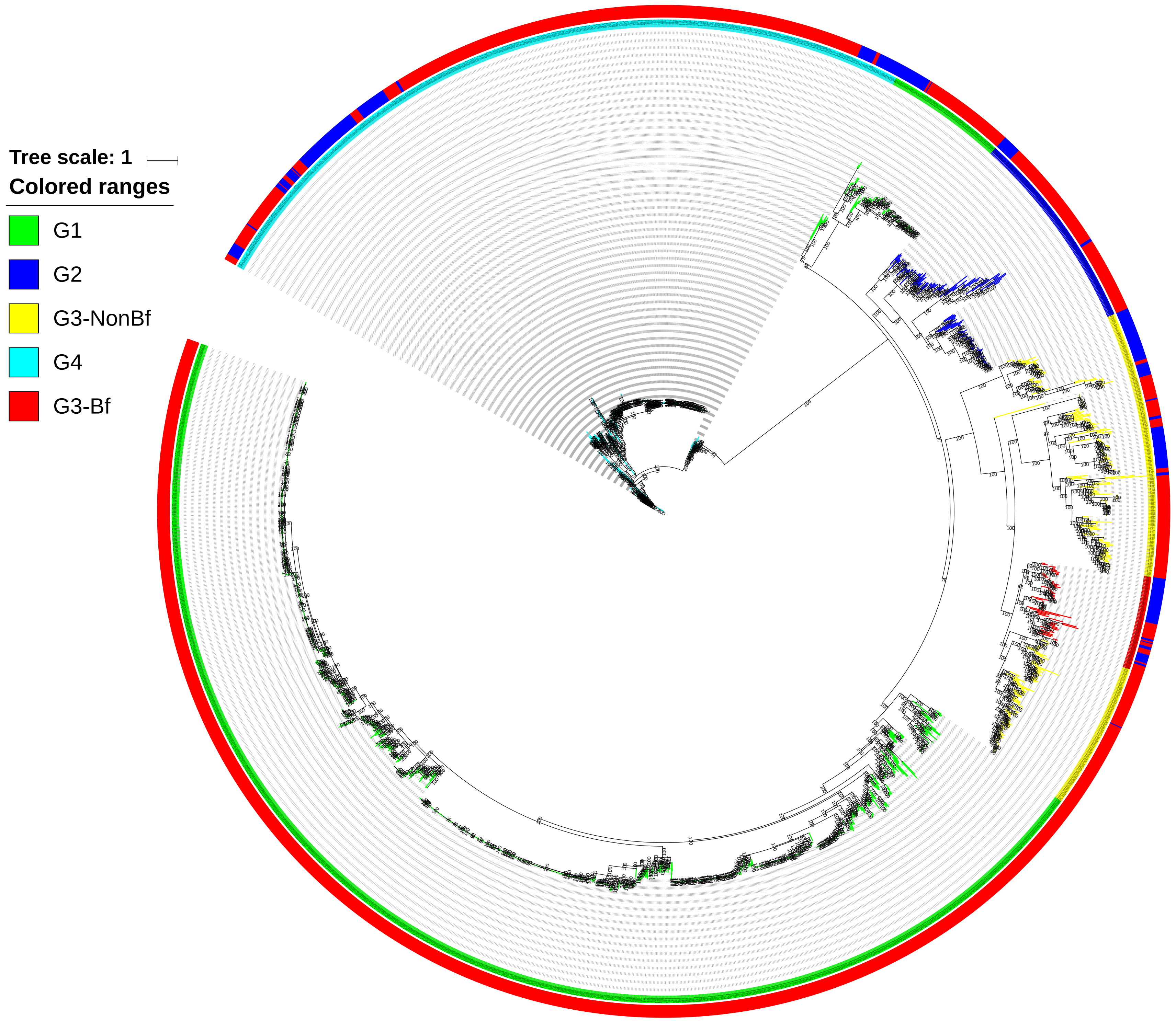

Supplementary Figure 7. Maximum-likelihood phylogenetic reconstruction of homologs of archaeal (blue in outer strip) and bacterial (red in outer strip) large subunits of Bf (i.e., MvhA that form group 3c) and non-Bf (groups 1, 2, 3a, 3b, 3d, and 4) [NiFe]-hydrogenase in complete genomes extracted from (Boyd et al., 2014). The phylogeny was by default rooted to the large subunit of group 4 by the RAxML which was used as the root to evaluate the evolution of MvhA homologs. The group designations for [NiFe]-hydrogenase are defined as in (Vignais et al., 2001; Boyd et al., 2014) and are represented by different colors at sequence terminals (inner ring and as projected on lineages). Bootstrap values for each node are shown as a percentage (out of 1000 bootstrap replicates).

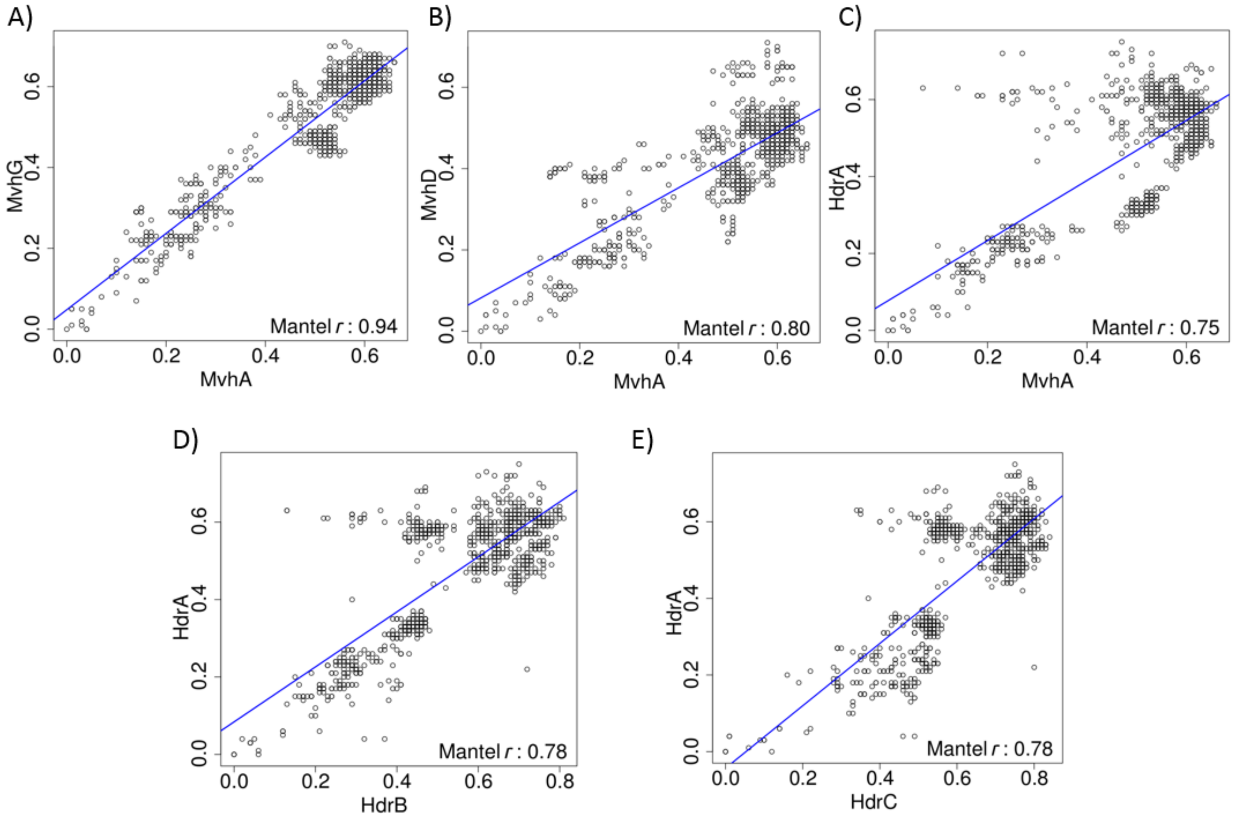

Supplementary Figure 8. Mantel regression of the pairwise distances of homologs of subunits of the Mvh complex plotted as a function of each other: **A**) MvhA and MvhG, **B**) MvhA and MvhD, **C**) MvhA and HdrA (only associated with Mvh complex), **D**) HdrA and HdrB (both associated with only Mvh complex), and **E**) HdrC and HdrA (both associated with only Mvh complex). Mantel *r* values are provided and the significance values (*p*-value) for all regressions shown were <0.001. Names for each abbreviated protein complex and their subunits are provided in **Table 1.**

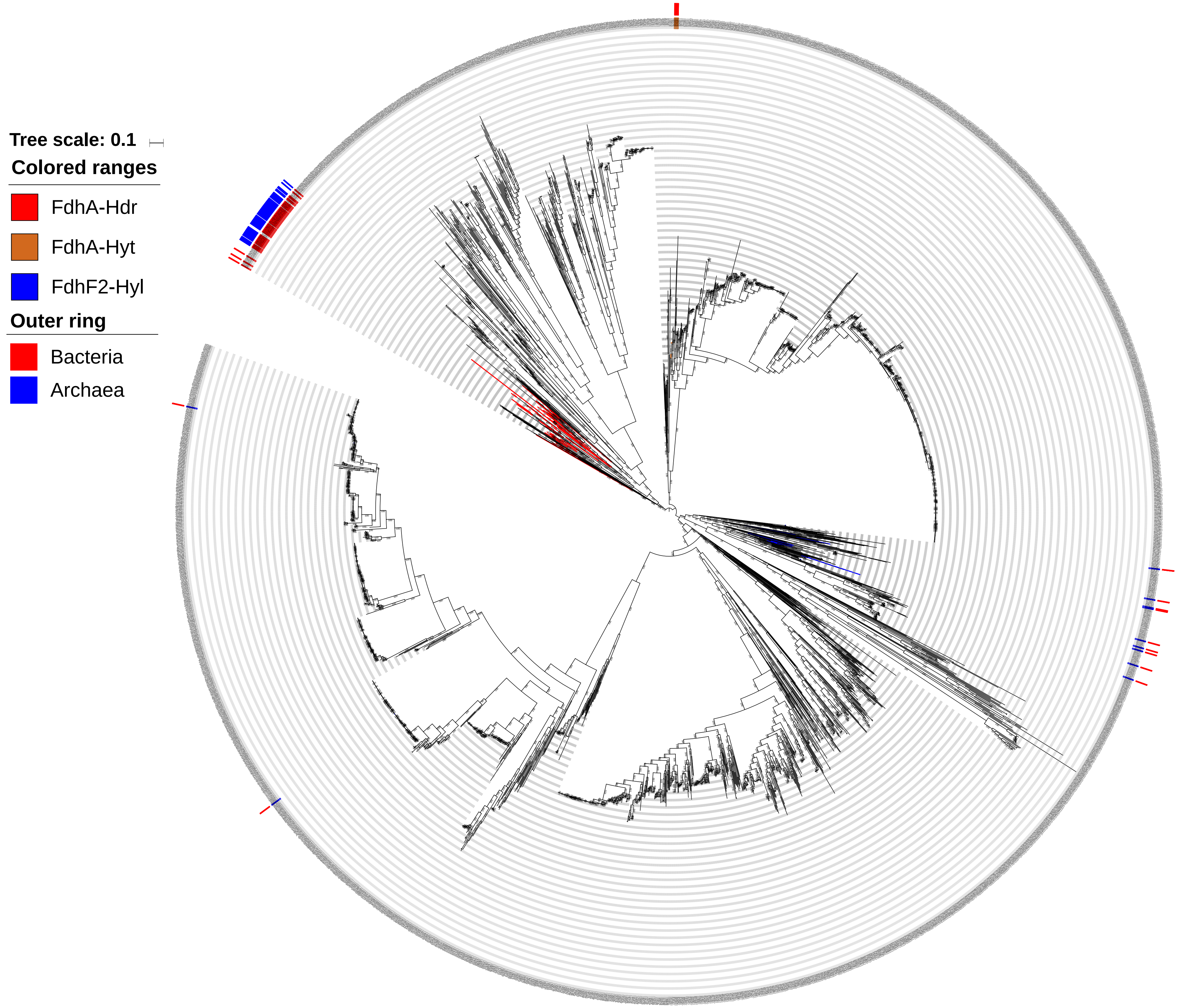

Supplementary Figure 9. Maximum-likelihood phylogenetic reconstruction of archaeal (blue in the outer strip) and bacterial (red in outer strip) Bf (FdhA-Hdr, FdhA-Hyt, and FdhF2-Hyl) and non-Bf FdhA homologs from complete genome sequences. Mid-point rooting was used since FdhA and FdhF2 are paralogous. Sequence terminals of homologs of bifurcating FdhA are color-coded, with FdhA-Hdr in red, FdhA-Hyt in rust, and FdhF2-Hyl in blue. Sequence terminals that are not colored depict FdhA homologs that are not predicted to bifurcate. Names for each abbreviated protein complexes are provided in **Table 1**. Bootstrap values for each node are shown as a percentage (out of 1000 bootstrap replicates).

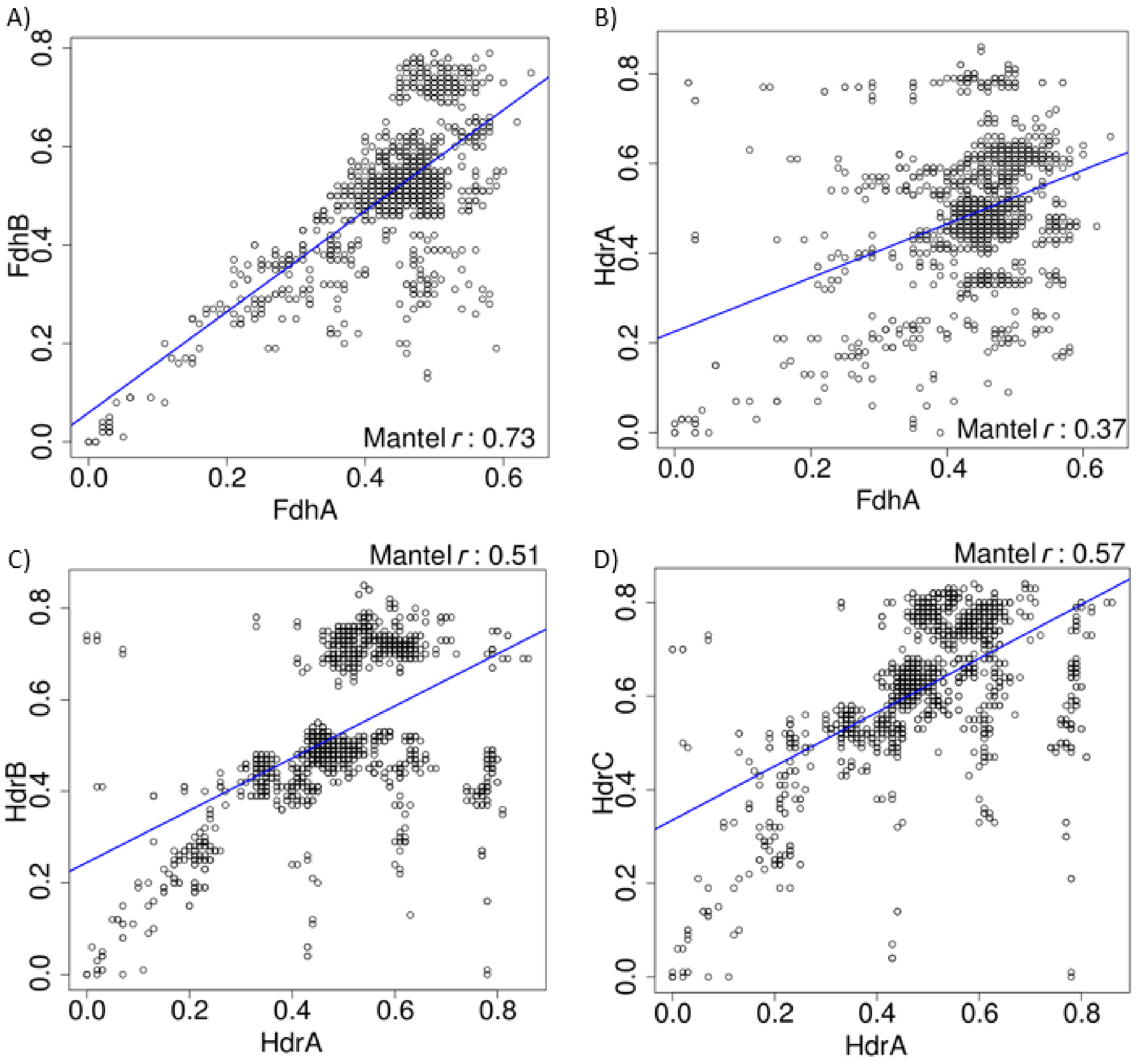

Supplementary Figure 10. Mantel regression of the pairwise distances of homologs of subunits of the Fdh complex plotted as a function of each other. **A**) FdhA and FdhB, **B**) FdhA and HdrA (only associated with Fdh complex), **C**) HdrB and HdrA (both only associated with Fdh complex), and **D**) HdrA and HdrC (both only associated with Fdh complex). Mantel *r* values are provided and the significance values (*p*-value) for all regressions shown were <0.001. Names for each abbreviated protein complex and their subunits are provided in **Table 1.**

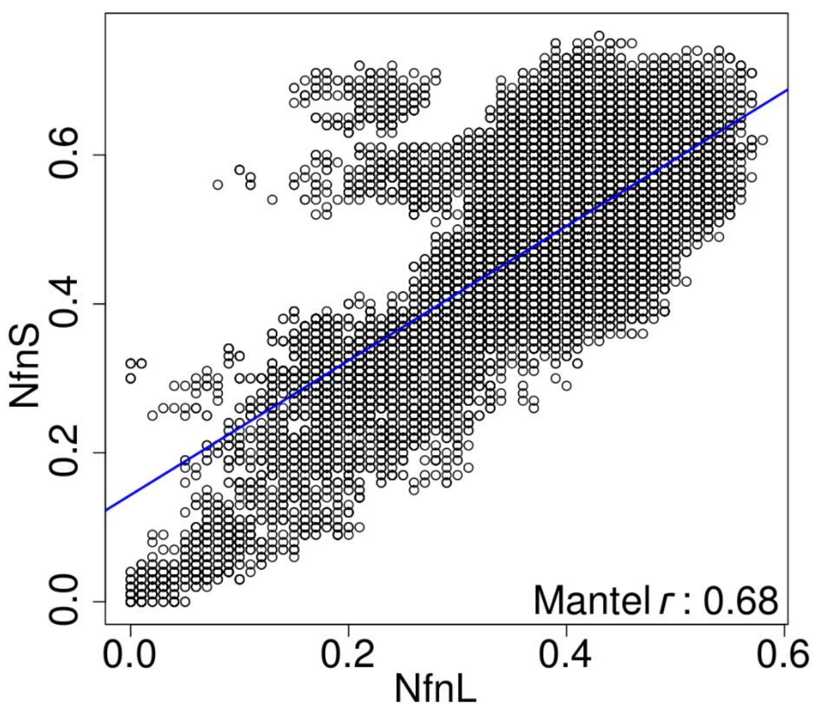

Supplementary Figure 11. Mantel regression of the pairwise distances of homologs of NfnS as a function of the pairwise distances of homologs of NfnL. The Mantel *r* value is provided and the significance value (*p*-value) for the regression shown was <0.001. Names for each abbreviated protein complex and their subunits are provided in **Table 1**.

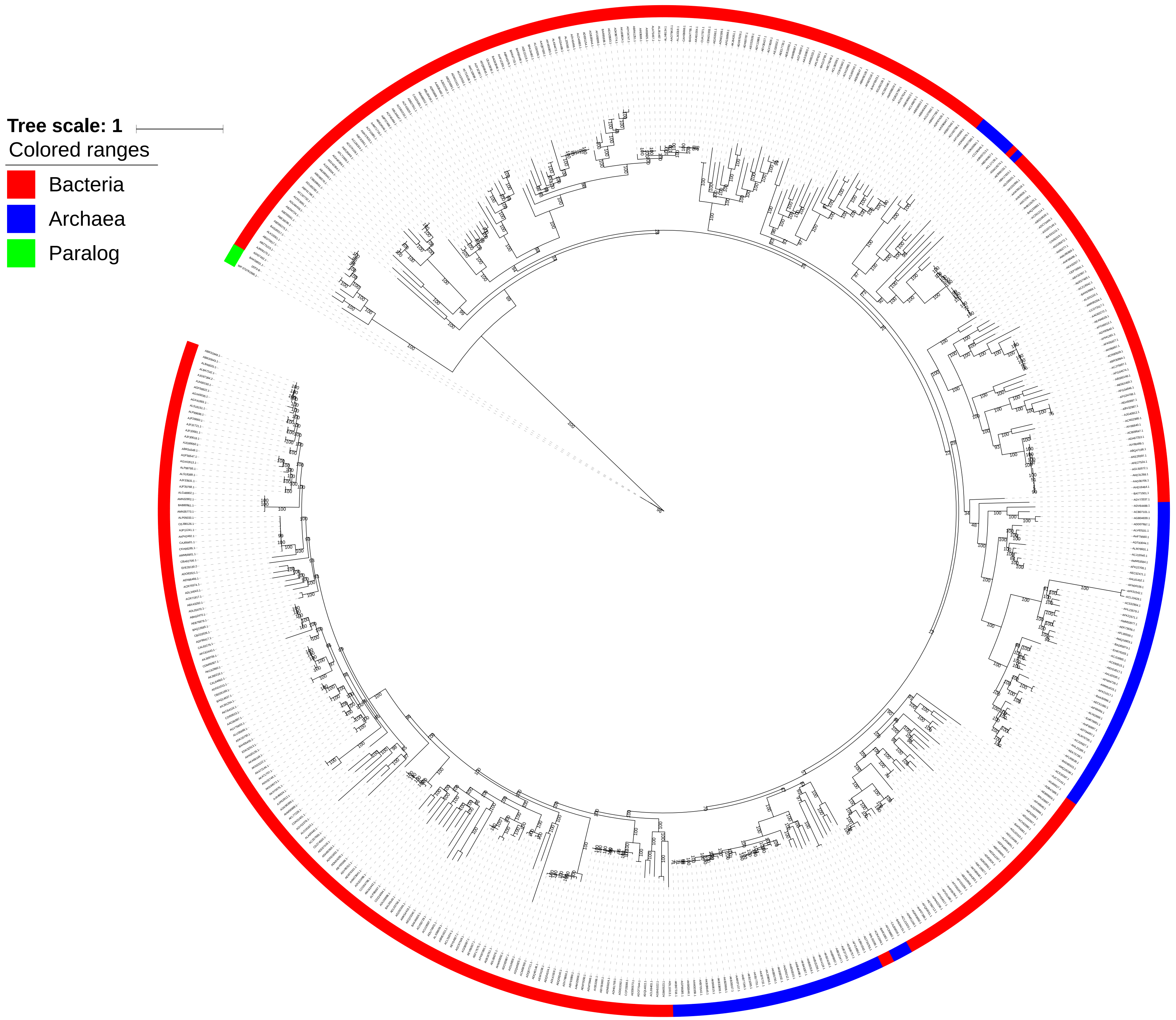

Supplementary Figure 12. Maximum-likelihood phylogenetic reconstruction of a concatenation of archaeal (blue in outer strip) and bacterial (red in outer strip) NfnSL in complete genome sequences rooted (green outer strip) with concatenated paralogous proteins which include dihydroorotate dehydrogenase from *Lactococcus lacticus* (WP_011835013) and glutamate synthase from *Azospirillum brasilense* (WP_035677957), dihydroorotate dehydrogenase from *Lactococcus garvieae* (BAK58851) and glutamate synthase from *Azospirillum oryzae* (WP_085087092), and dihydroorotate dehydrogenase from *Floricoccus tropicus* (WP_070791886) and glutamate synthase from *Azospirillum humicireducens* (WP_063635528). Bootstrap values for each node are shown as a percentage (out of 1000 bootstrap replicates).

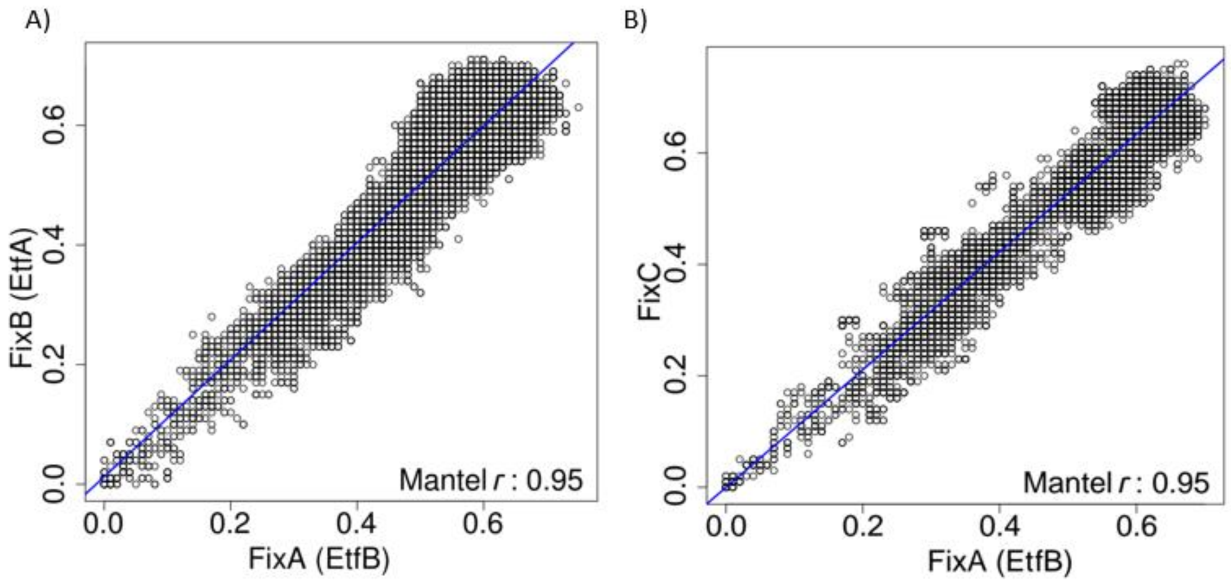

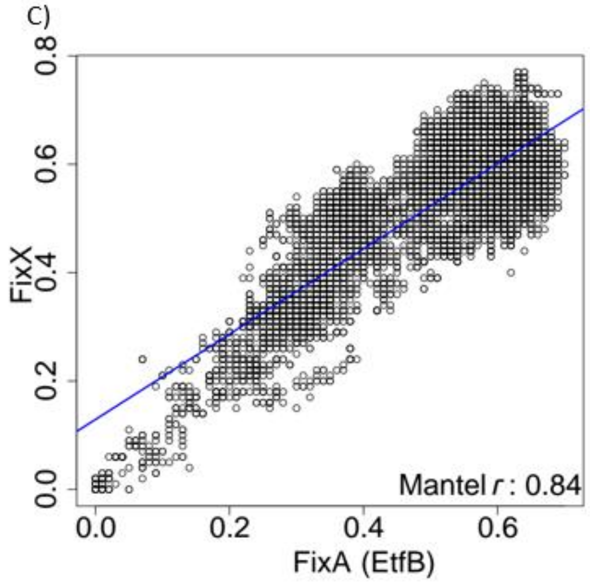

Supplementary Figure 13. Mantel regression of the pairwise distances of homologs of subunits of the Fix complex plotted as a function of each other. **A**) FixA (or only EtfB associated with Fix complex) and FixB (or only EtfA associated with Fix complex), **B**) FixC and FixA (or only EtfB associated with Fix complex), **C**) FixX and FixA (or only EtfB associated with Fix complex), Mantel *r* values are provided and the significance values (*p*-value) for all regressions shown were <0.001. Names for each abbreviated protein complex and their subunits are provided in **Table 1.**

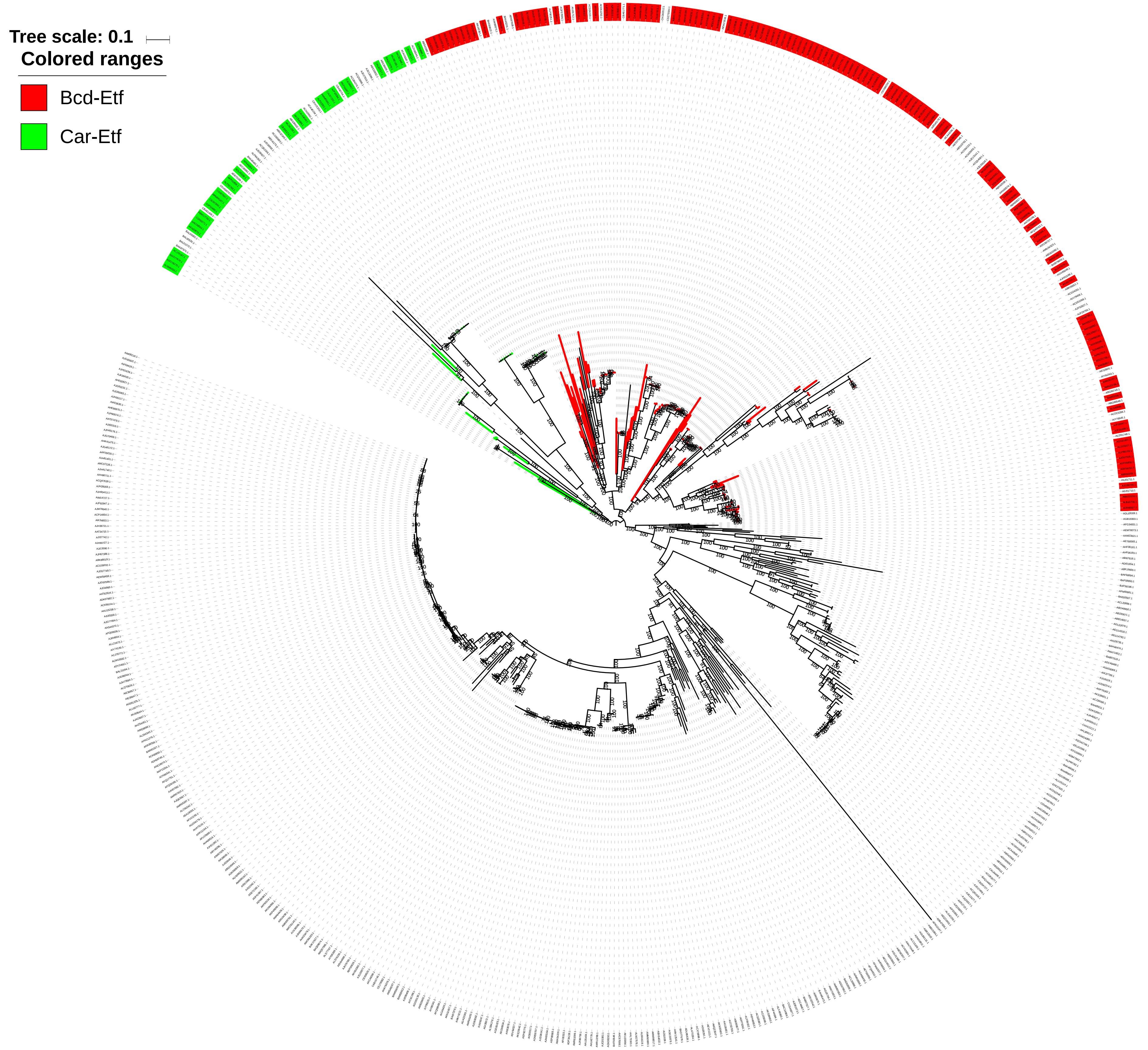

Supplementary Figure 14. Maximum-likelihood phylogenetic reconstruction of butyryl-CoA dehydrogenase (Bcd) (red outer strip) and CarC (green outer strip) subunits that are associated with Etf in complete genomes and proposed to bifurcate and those that are not associated with Etf and thus are predicted to not bifurcate (terminals are uncolored). Mid-point rooting was used since Bcd and CarC are paralogous. Names for each abbreviated protein complexes are provided in **Table 1**. Note, homologs of Bcd and CarC subunits associated with Etf were only detected among Bacteria, thereby negating the need to add a colored strip demarcating archaeal and bacterial homologs. Bootstrap values for each node are shown as a percentage (out of 1000 bootstrap replicates).

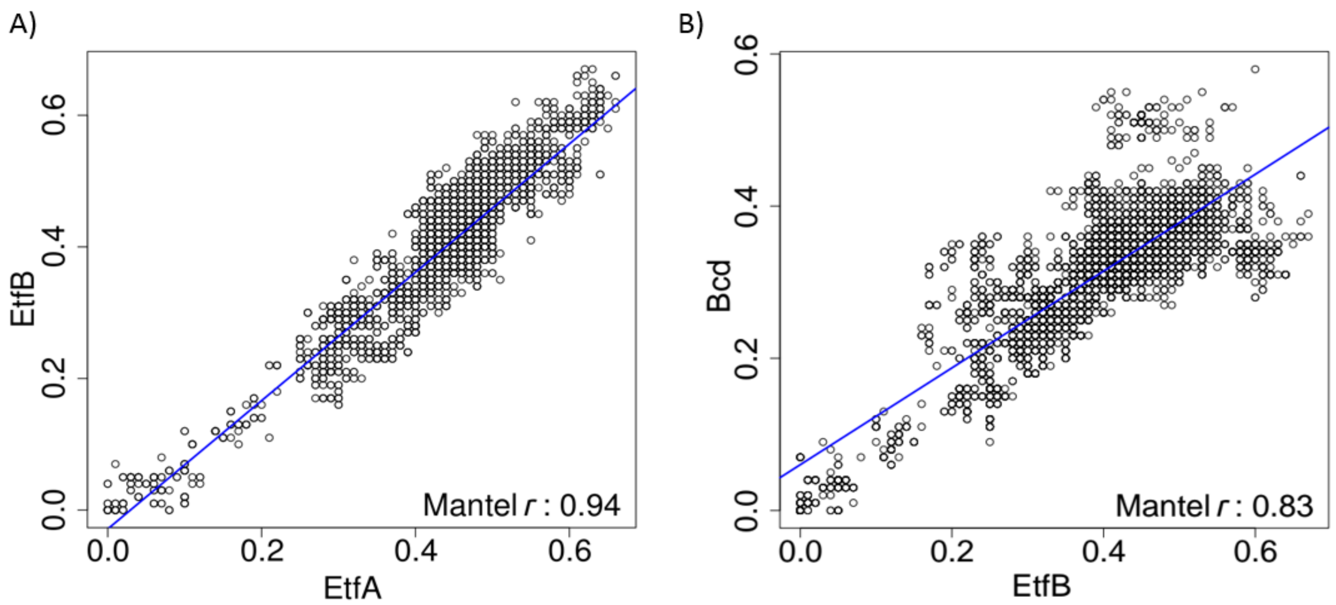

Supplementary Figure 15. Mantel regression of the pairwise distances of homologs of subunits of the Bf-Bcd complex plotted as a function of each other:**A**) EtfB and EtfA (both of which are associated with only Bf-Bcd complex), **B**) EtfB (associated with only Bf-Bcd complex) and Bcd. Mantel *r* values are provided and the significance values (*p*-value) for all regressions shown were <0.001. Names for each abbreviated protein complex and their subunits are provided in **Table 1.**

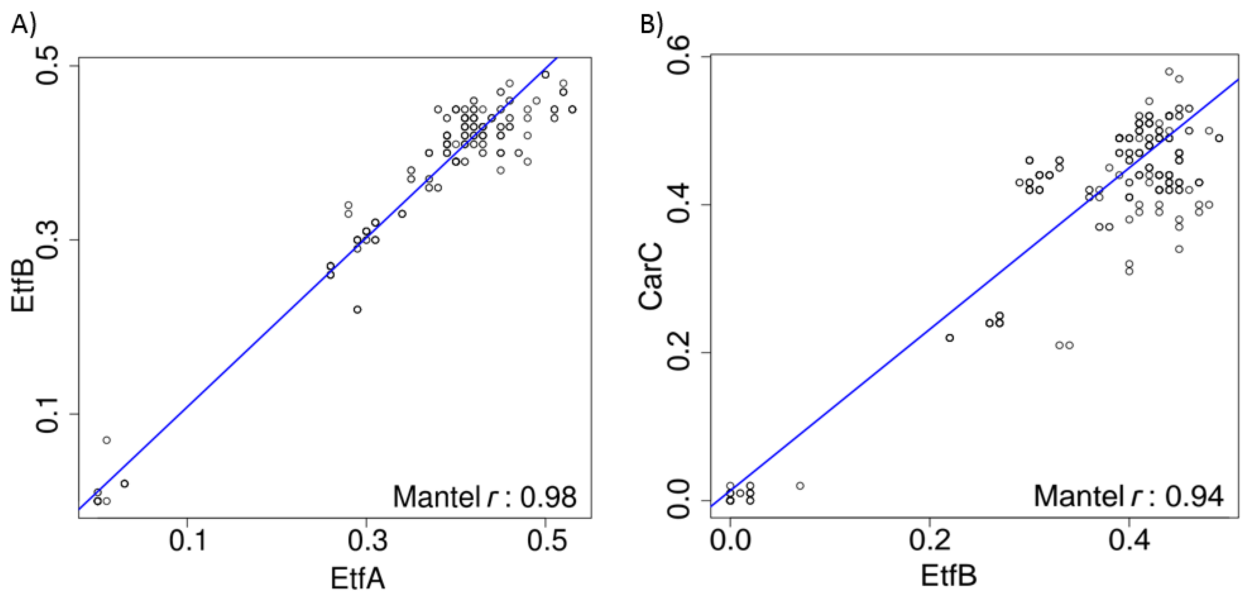

Supplementary Figure 16. Mantel regression of the pairwise distances of homologs of subunits of the Car complex plotted as a function of each other: **A**) EtfB and EtfA (both of which are associated with only Car complex), **B**) EtfB (associated with only Car complex) and CarC. Mantel *r* values are provided and the significance values (*p*-value) for all regressions shown were <0.001. Names for each abbreviated protein complex and their subunits are provided in **Table 1.**

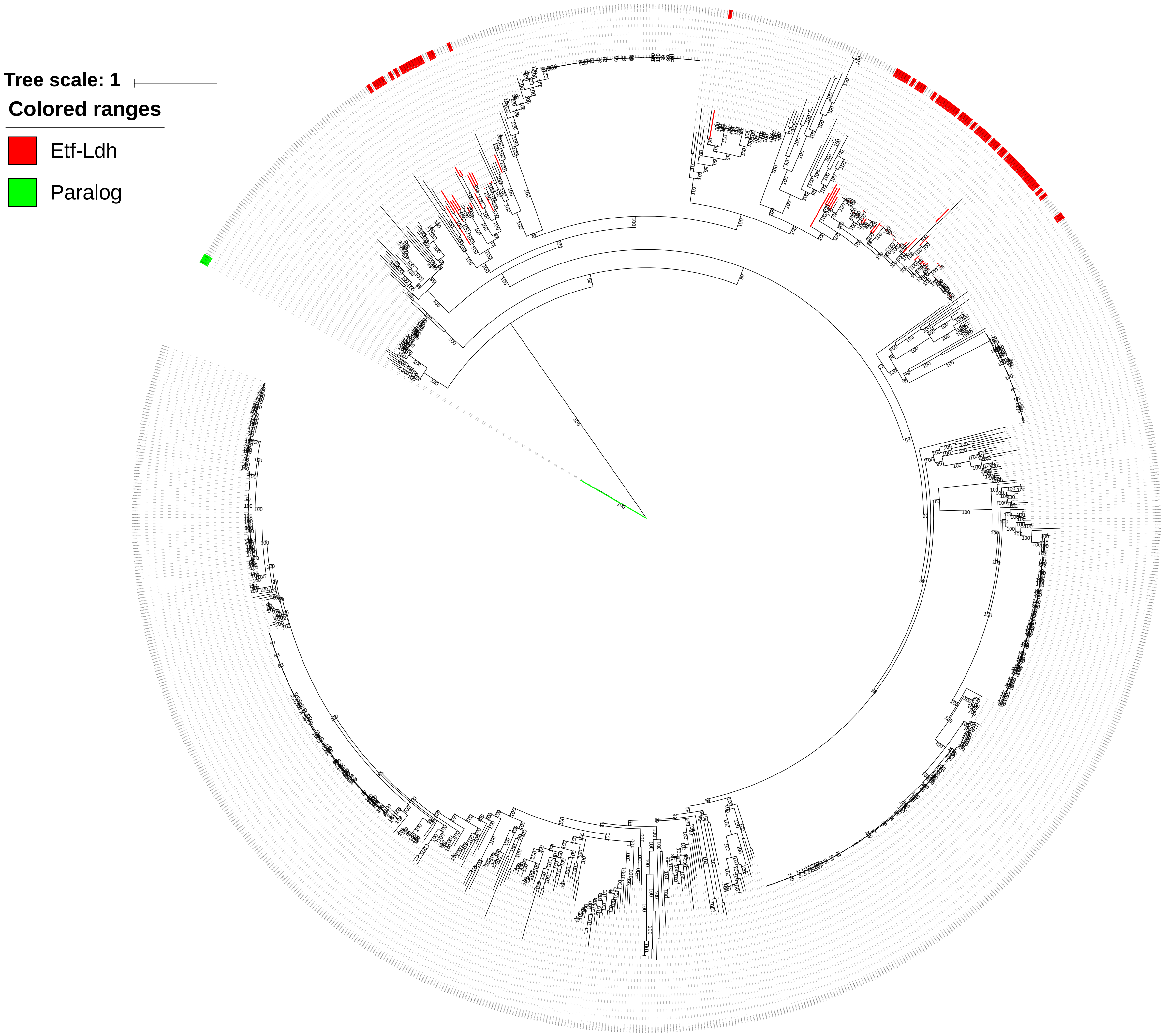

Supplementary Figure 17. Maximum-likelihood phylogenetic reconstruction of lactate dehydrogenase (Ldh) homologs that associate with Etf in complete genomes and are predicted to bifurcate (terminals colored in red, designated as Etf-Ldh) and those that do not associate with Etf and thus are not predicted to bifurcate (terminals are uncolored). The tree was rooted with the paralog (terminals colored in green) alkyl dihyroxyacetone phosphate synthase from *Dictyostelium discoideum* (XP_637836) and *Trypanosoma brucei* (XP_845272). Names for each abbreviated protein complexes are provided in **Table 1**. Note, homologs of Ldh associated with Etf were only detected among Bacteria, thereby negating the need to add a colored strip demarcating archaeal and bacterial homologs. Bootstrap values for each node are shown as a percentage (out of 1000 bootstrap replicates).

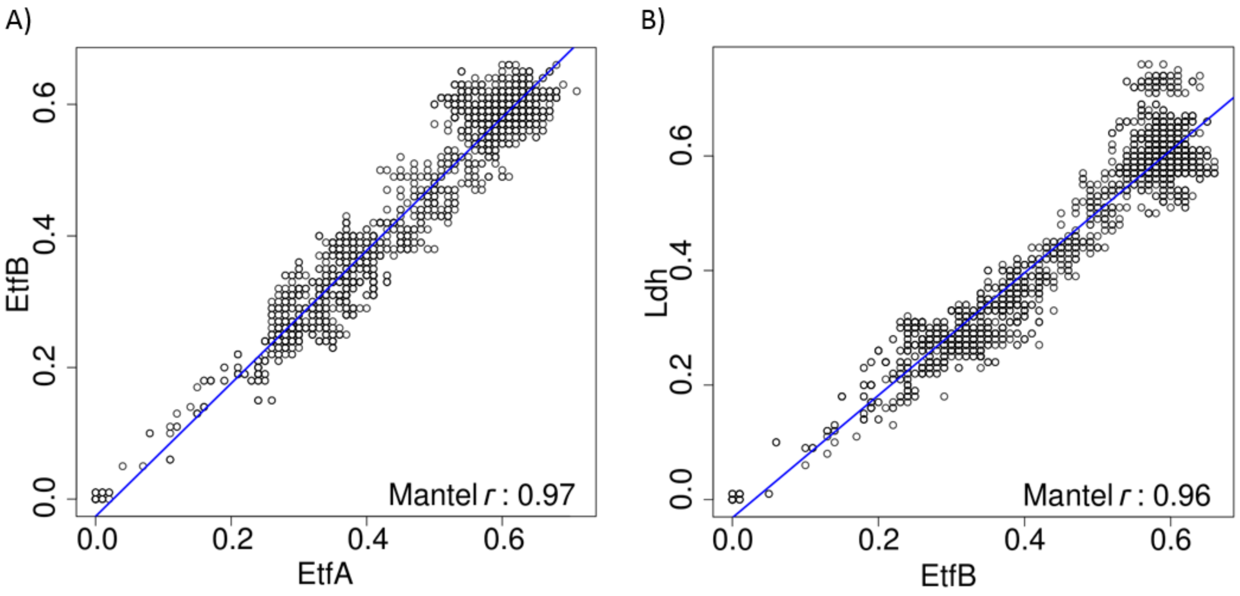

Supplementary Figure 18. Mantel regression of the pairwise distances of homologs of subunits of the Bf-Ldh complex plotted as a function of each other: **A**) EtfB and EtfA (both of which are associated with only Bf-Ldh complex), **B**) EtfB (associated with only Bf-Ldh complex) and Ldh. Mantel *r* values are provided and the significance values (*p*-value) for all regressions shown were <0.001. Names for each abbreviated protein complex and their subunits are provided in **Table 1.**

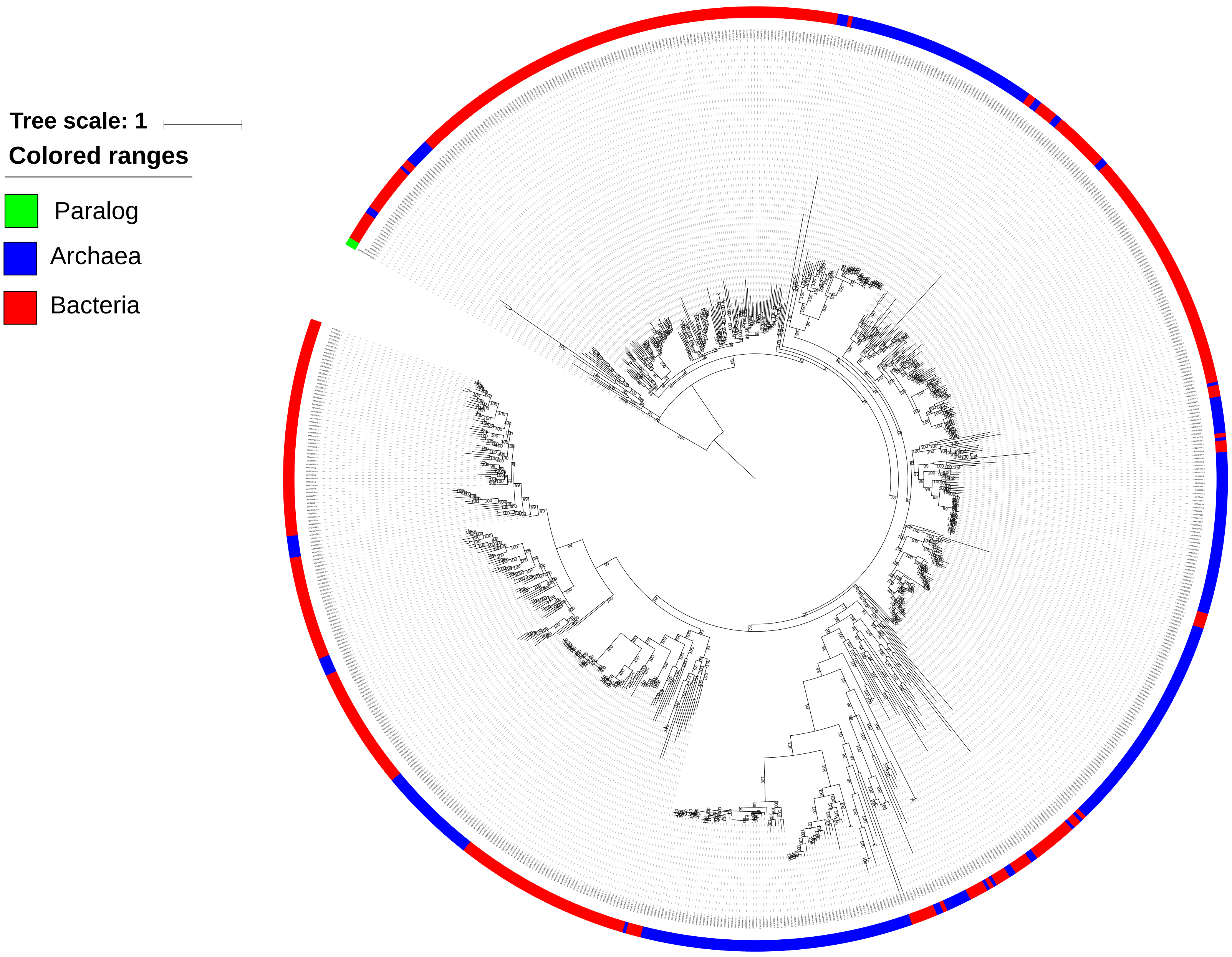

Supplementary Figure 19. Maximum-likelihood phylogenetic reconstruction of all Hdr homologs that associate with Fdh, Met, Mvh, and Hdr2 that functions in complete bacterial (red in outer strip) and archaeal (blue in outer strip) genomes. The tree was rooted with the paralog (terminals colored in green) thioredoxin reductase from *Escherichia coli* (P0A9P4) and *Shigella* sp. (WP_000537416). Names for each abbreviated protein complexes are provided in **Table 1**. Bootstrap values for each node are shown as a percentage (out of 1000 bootstrap replicates).

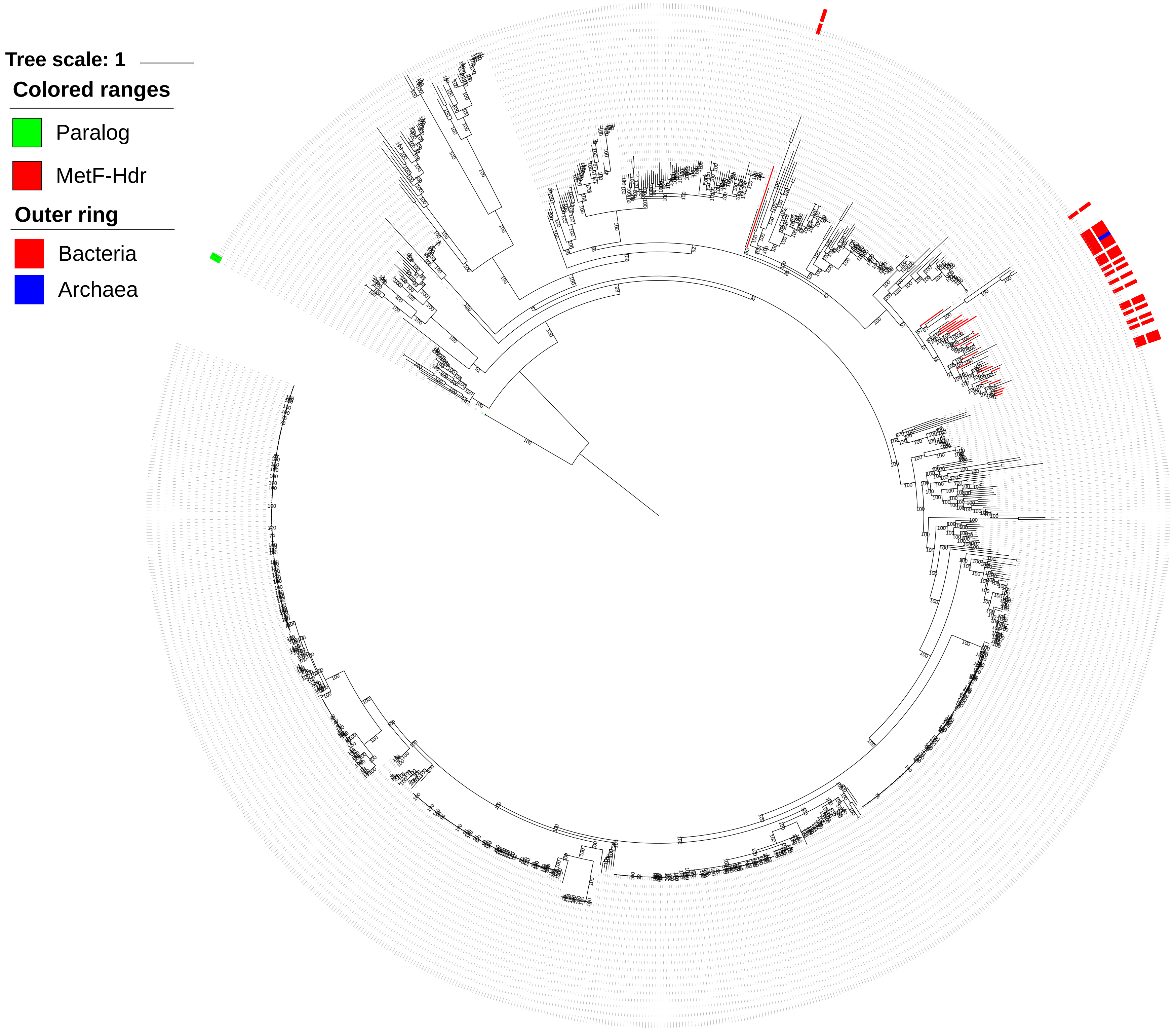

Supplementary Figure 20. Maximum-likelihood phylogenetic reconstruction of MetF homologs that associate with Hdr in complete bacterial (red in outer strip) and archaeal (blue in outer strip) genomes and are predicted to bifurcate (terminals colored in red). Those homologs that do not associate with Hdr and thus are not predicted to bifurcate are represented by uncolored terminals. The tree was rooted with the paralog (terminals colored in green) methylenetetrahydrofolate reductase from *Escherichia coli* (WP_089580839) and *Shigella flexneri* (OUZ65700). Names for each abbreviated protein complexes are provided in **Table 1**. Bootstrap values for each node are shown as a percentage (out of 1000 bootstrap replicates).

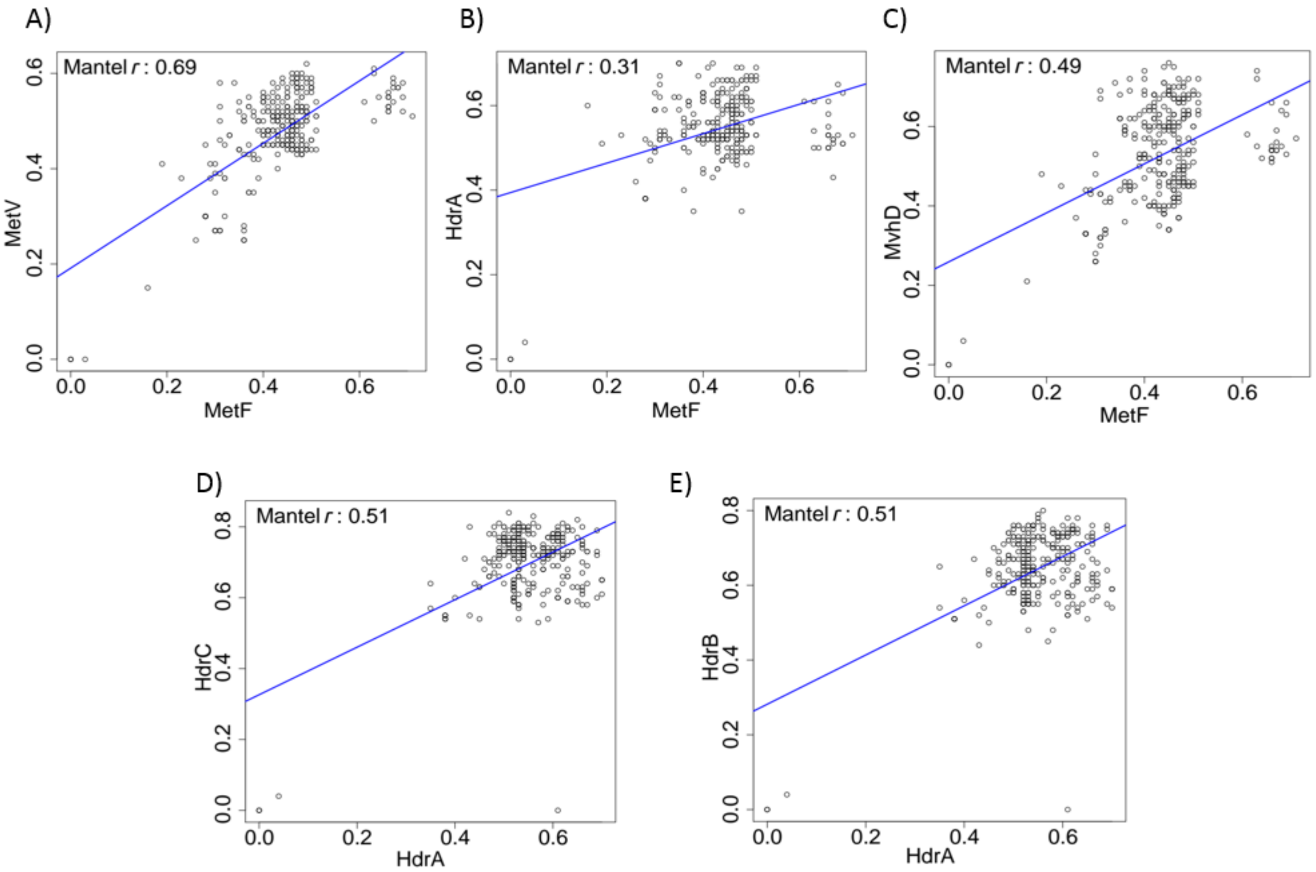

Supplementary Figure 21. Mantel regression of the pairwise distances of homologs of the subunits of Met plotted as a function of each other: **A**) MetF and MetV, **B**) MetF and HdrA, **C**) MetF and MvhD, **D**) HdrA and HdrC, **E**) HdrA and HdrB. Mantel *r* values are provided and the significance values (*p*-value) for all regressions shown were <0.001. Names for each abbreviated protein complex and their subunits are provided in **Table 1.**

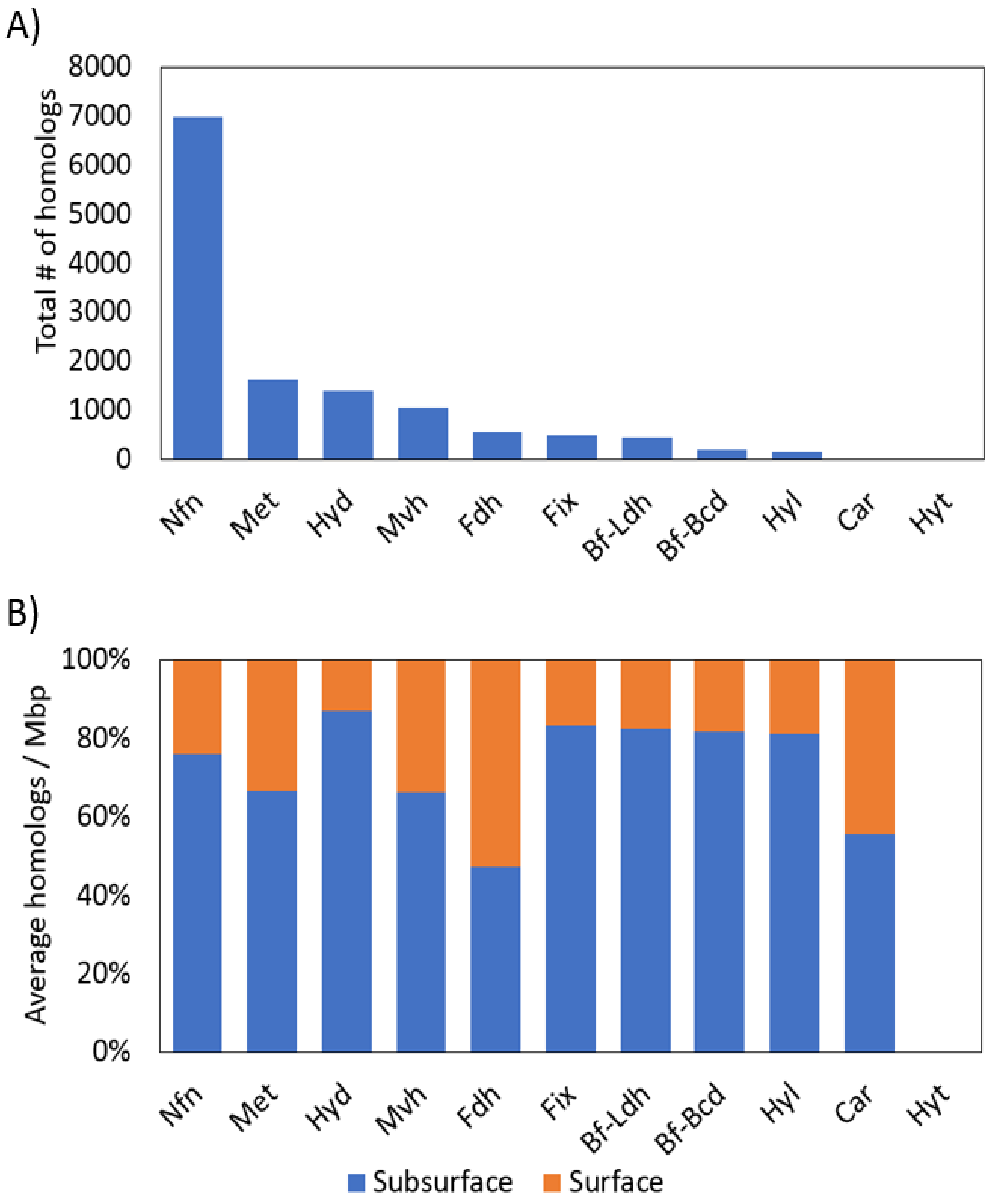

Supplementary Figure 22. Abundance and distribution of homologs of putative Bf enzymes among metagenomic sequences (n = 3,136 total metagenomes). **A**) Histogram depicting the total number of homologs for the specified type of Bf enzyme complex. **B**) The average abundances of homologs of all Bf enzyme complexes in non-redundant metagenomic contigs that were classified as 'surface' and 'subsurface' were normalized to the total number of megabase (Mb) pairs of sequence. Names for protein complexes are provided in **Table 1**.

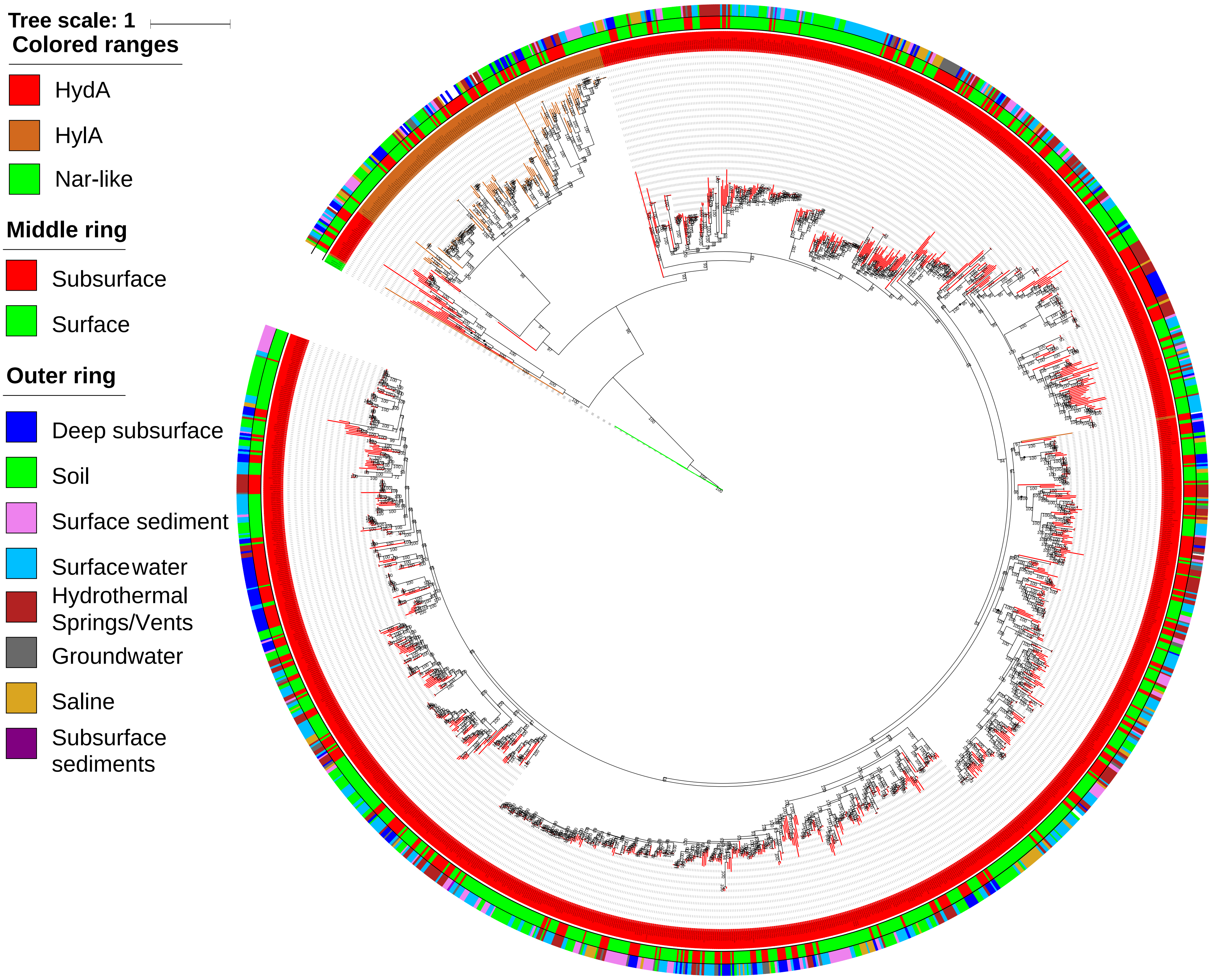

Supplementary Figure 23. Maximum-likelihood phylogenetic reconstruction of Bf homologs of Hyd (HydA and HylA; homologs of HytA were not identified among metagenomic sequences) in metagenomic sequences. The phylogeny was rooted with Nar-like proteins from *Homo sapiens* (NP_071938 and NP_036468), *Danio rerio* (A2RRV9), *Thalassiosira pseudonana* (XP_002289272) and *Ostreococcus lucimarinus* (XP_001416706). Sequence terminals are color coded, with the outer ring indicating the environment type where a given homolog was identified, the middle ring indicating whether the environment type that the homolog was identified in was classified as a surface or subsurface environment, and the inner ring indicating homology to HydA (highlighted in red), HylA (highlighted in rust), or the paralog Nar-like proteins (highlighted in green). If a protein homolog was identified in a metagenome that lacked metadata allowing for coding of the outer or middle ring, it was left blank (white). Names of abbreviated protein complexes are provided in **Table 1.** Note, monomeric (non-Bf) HydA or HylA were not included in this analysis. Bootstrap values for each node are shown as a percentage (out of 1000 bootstrap replicates).

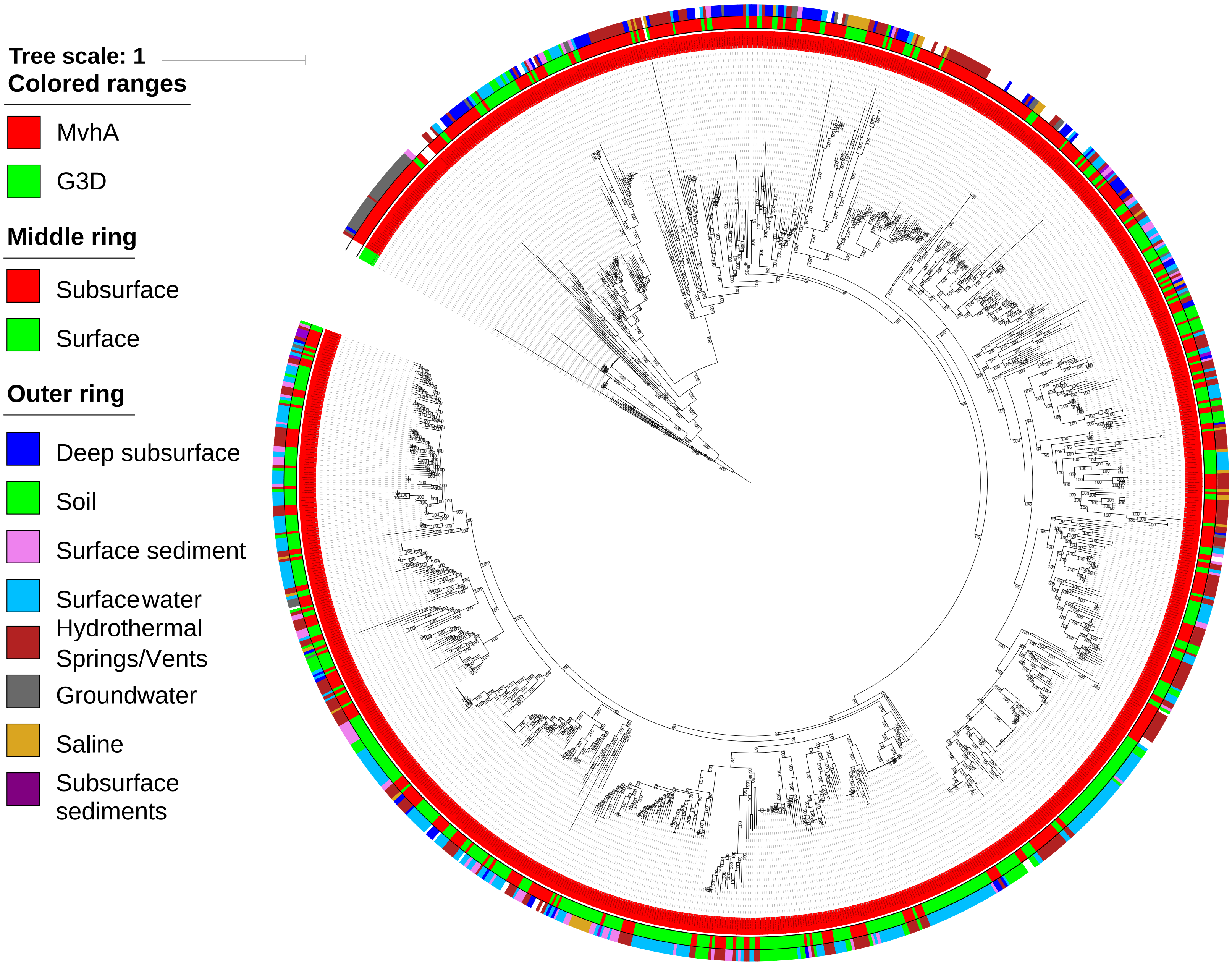

Supplementary Figure 24. Maximum-likelihood phylogenetic reconstruction of homologs of the large subunit of the Mvh complex (i.e., MvhA) identified in metagenomic sequences. The phylogeny was rooted with the large subunit of group 3d non-bifurcating [NiFe]-hydrogenase from *Cupriavidus necator* (WP_011154013), *Azoarcus olearius* (WP_011765148), *Paraburkholderia xenovorans* (WP_040123534), *Psychromonas ingrahamii* (WP_041766077), and *Rhodobacter capsulatus* (AAD38065). Sequence terminals are color coded, with the outer ring indicating the environment type where a given homolog was identified, the middle ring indicating whether the environment type that the homolog was identified in was classified as a surface or subsurface environment, and the inner ring indicating homology to MvhA (highlighted in red) or the paralog (group 3d non-Bf [NiFe]-hydrogenase highlighted in green). Protein homologs from metagenomes that lacked environmental classification were not colored in the middle and outer rings. Names of abbreviated protein complexes are provided in **Table 1.** Bootstrap values for each node are shown as a percentage (out of 1000 bootstrap replicates).

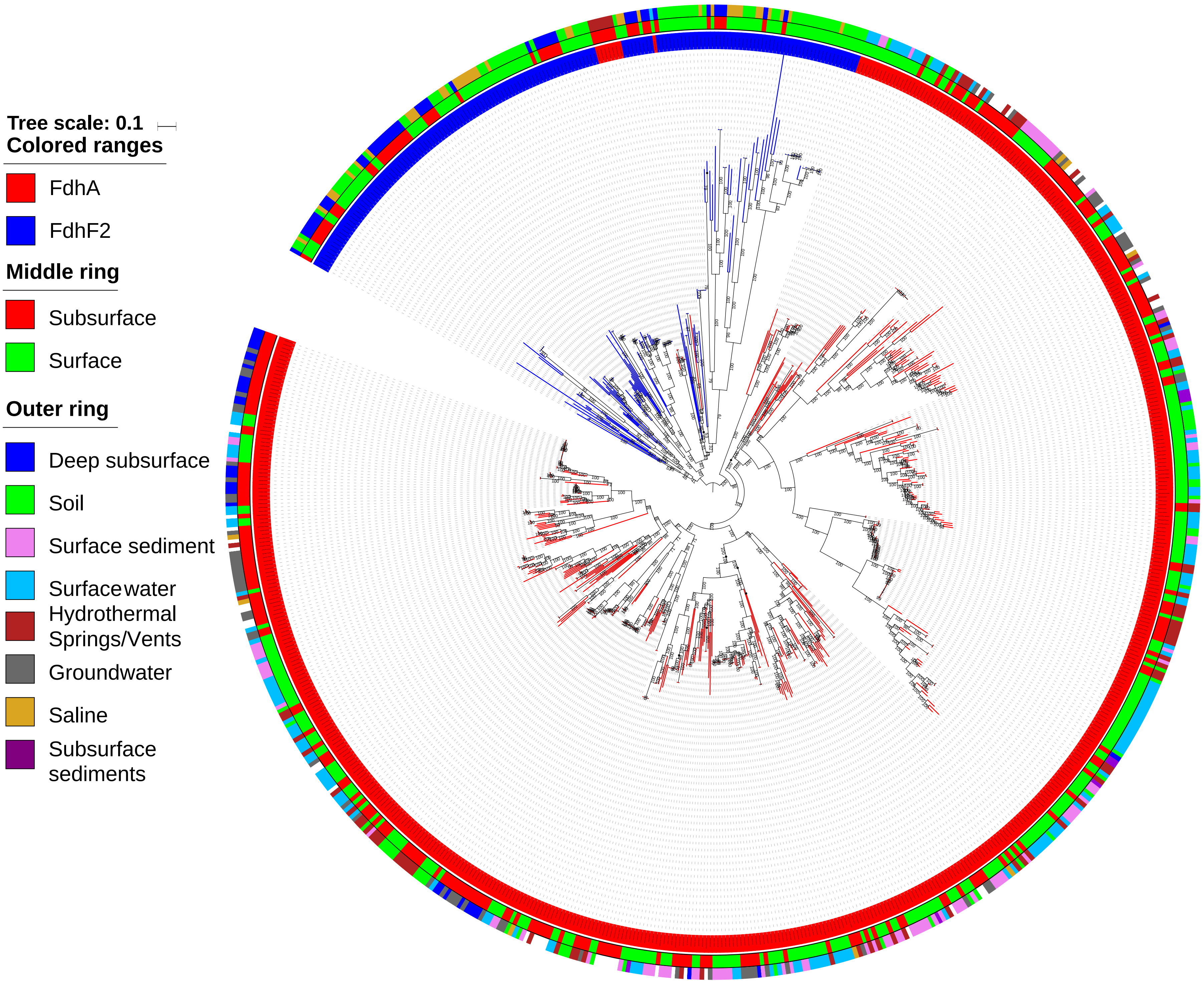

Supplementary Figure 25. Maximum-likelihood phylogenetic reconstruction of FdhA homologs that are predicted to form a Bf complex (i.e., Hyl and Hdr) in metagenomic sequences. FdhA homologs that are predicted to form a Bf complex with Hyt were not identified in metagenomic sequences and thus are not depicted. Mid-point rooting was used since FdhA and FdhF2 are paralogous. Sequence terminals are color coded, with the outer ring indicating the environment type where a given homolog was identified, the middle ring indicating whether the environment type that the homolog was identified in was classified as a surface or subsurface environment, and the inner ring indicating homology to FdhA (Hdr highlighted in red) or FdhF2 (Hyl highlighted in green). Protein homologs from metagenomes that lacked environmental classification were not colored in the middle and outer rings. Names of abbreviated protein complexes are provided in **Table 1.** Bootstrap values for each node are shown as a percentage (out of 1000 bootstrap replicates).

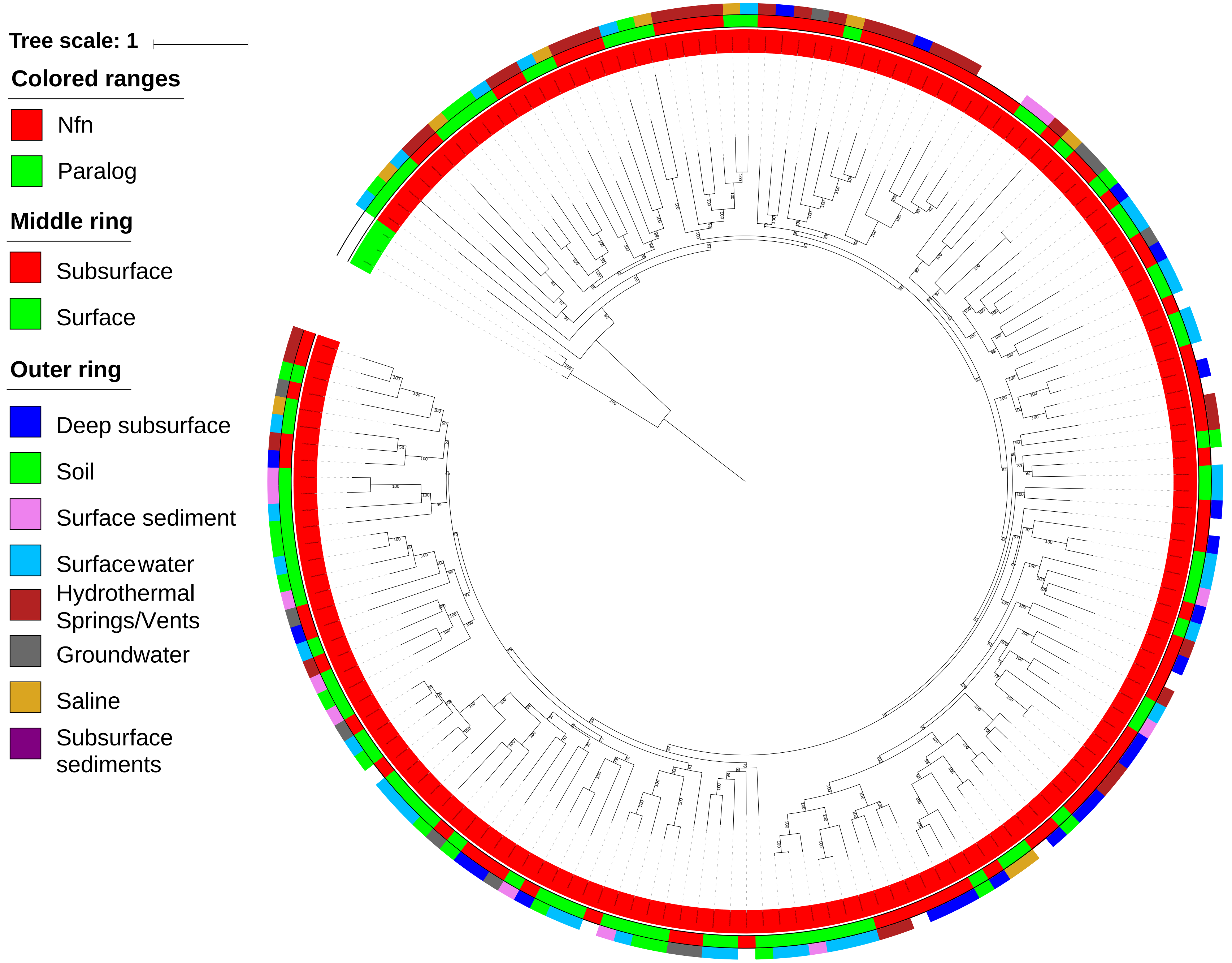

Supplementary Figure 26. Maximum-likelihood phylogenetic reconstruction of a concatenation of representative NfnSL homologs of each homolog 'bin' generated using a CD-HIT clustering approach applied to homologs identified in metagenomic sequences. All NfnSL homologs identified in metagenomes were first clustered into unique homolog 'bins' that contained closely related NfnSL homologs and the representative sequences of each 'bin' were extracted to reconstruct the phylogenetic tree of NfnSL. The phylogeny was rooted (green outer strip) with concatenated paralogous proteins which include dihydroorotate dehydrogenase from *Lactococcus lacticus* (WP_011835013) and glutamate synthase from *Azospirillum brasilense* (WP_035677957), dihydroorotate dehydrogenase from *Lactococcus garvieae* (BAK58851) and glutamate synthase from *Azospirillum oryzae* (WP_085087092), and dihydroorotate dehydrogenase from *Floricoccus tropicus* (WP_070791886) and glutamate synthase from *Azospirillum humicireducens* (WP_063635528). Sequence terminals are color coded, with the outer ring indicating the environment type where a given homolog was identified, the middle ring indicating whether the environment type that the homolog was identified in was classified as a surface or subsurface environment, and the inner ring indicating homology to Nfn (highlighted in red) or the paralog (dihydroorotate dehydrogenase highlighted in green). Protein homologs from metagenomes that lacked environmental classification were not colored in the middle and outer rings. Names of abbreviated protein complexes are provided in **Table 1.** Bootstrap values for each node are shown as a percentage (out of 1000 bootstrap replicates).

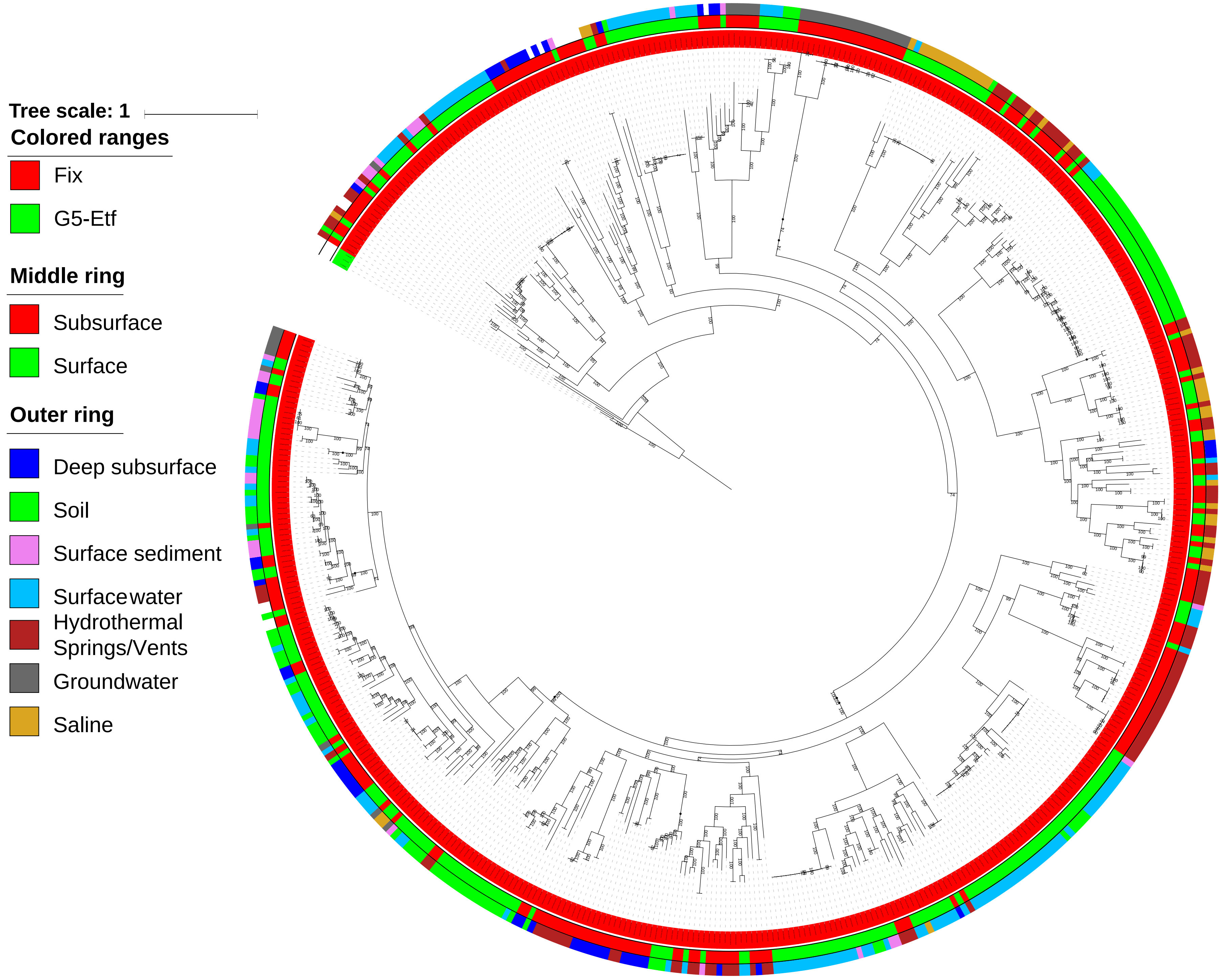

Supplementary Figure 27. Maximum-likelihood phylogenetic reconstruction of homologs of putative Bf EtfAB (i.e., Fix) identified in metagenomic sequences. The phylogeny was rooted with homologs of group 5 EtfAB from *Desulfitobacterium hafniense* (BAE84276-BAE84277), *Desulfotomaculum reducens* (ABO50304-ABO50305) and *Geobacillus kaustophilus* (BAD76971-BAD76972) that are not predicted to bifurcate (Garcia Costas et al., 2017). Sequence terminals are color coded, with the outer ring indicating the environment type where a given homolog was identified, the middle ring indicating whether the environment type that the homolog was identified in was classified as a surface or subsurface environment, and the inner ring indicating homology to Fix (highlighted in red) or the paralog (non-Bf Etf highlighted in green). Protein homologs from metagenomes that lacked environmental classification were not colored in the middle and outer rings. Names of abbreviated protein complexes are provided in **Table 1.** Bootstrap values for each node are shown as a percentage (out of 1000 bootstrap replicates).

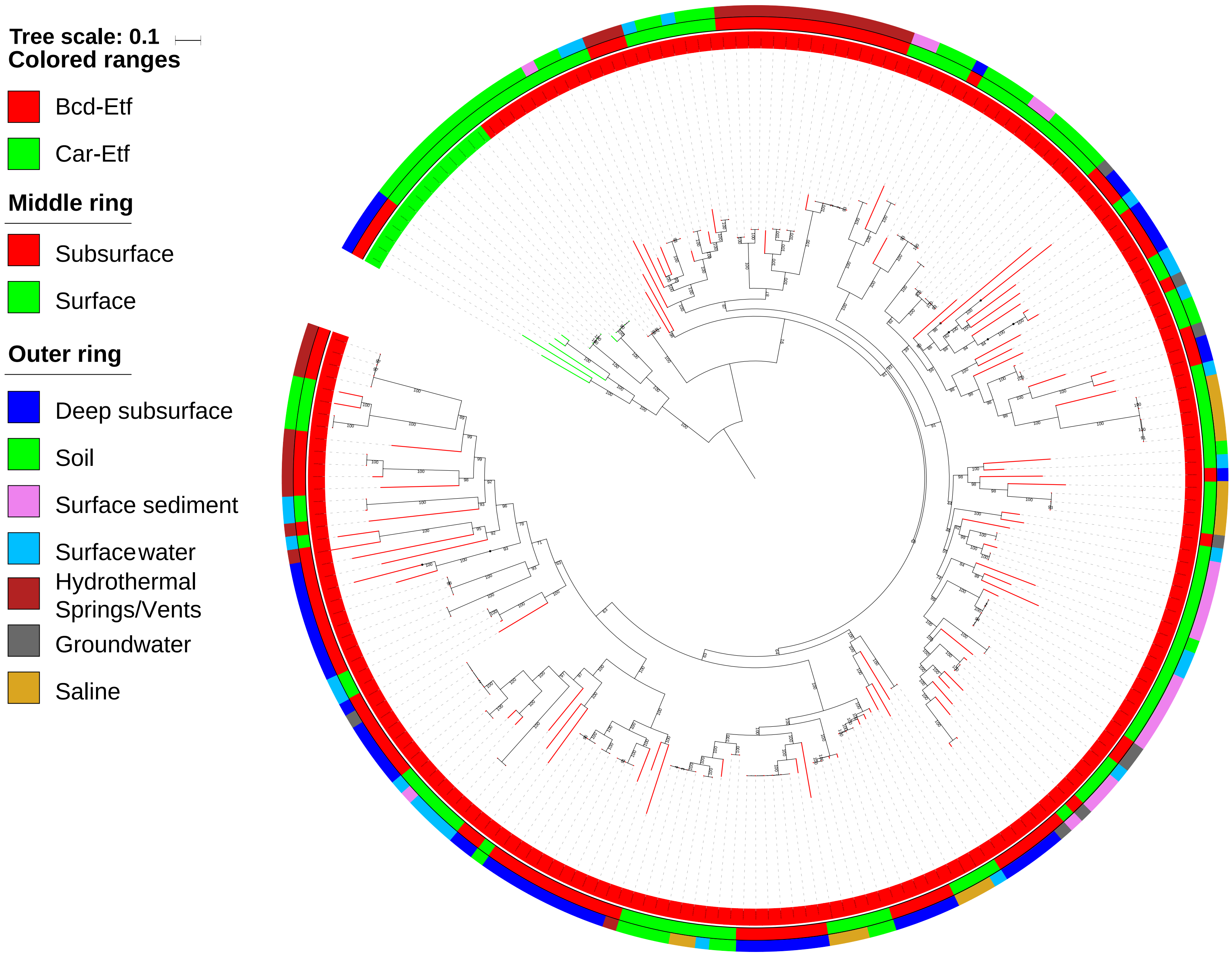

Supplementary Figure 28. Maximum-likelihood phylogenetic reconstruction of homologs of butyryl-CoA dehydrogenase (Bcd) and CarC identified in metagenomic sequences. Since, CarC and Bcd are paralogous enzymes, midpoint rooting was used. Sequence terminals are colored coded, with the outer ring indicating the environment type where a given homolog was identified, the middle ring indicating whether the environment type that the homolog was identified in was classified as a surface or subsurface environment, and the inner ring indicating homology to Bcd (highlighted in red) or Car (highlighted in green). Protein homologs from metagenomes that lacked environmental classification were not colored in the middle and outer rings. Names of abbreviated protein complexes are provided in **Table 1.** Bootstrap values for each node are shown as a percentage (out of 1000 bootstrap replicates).

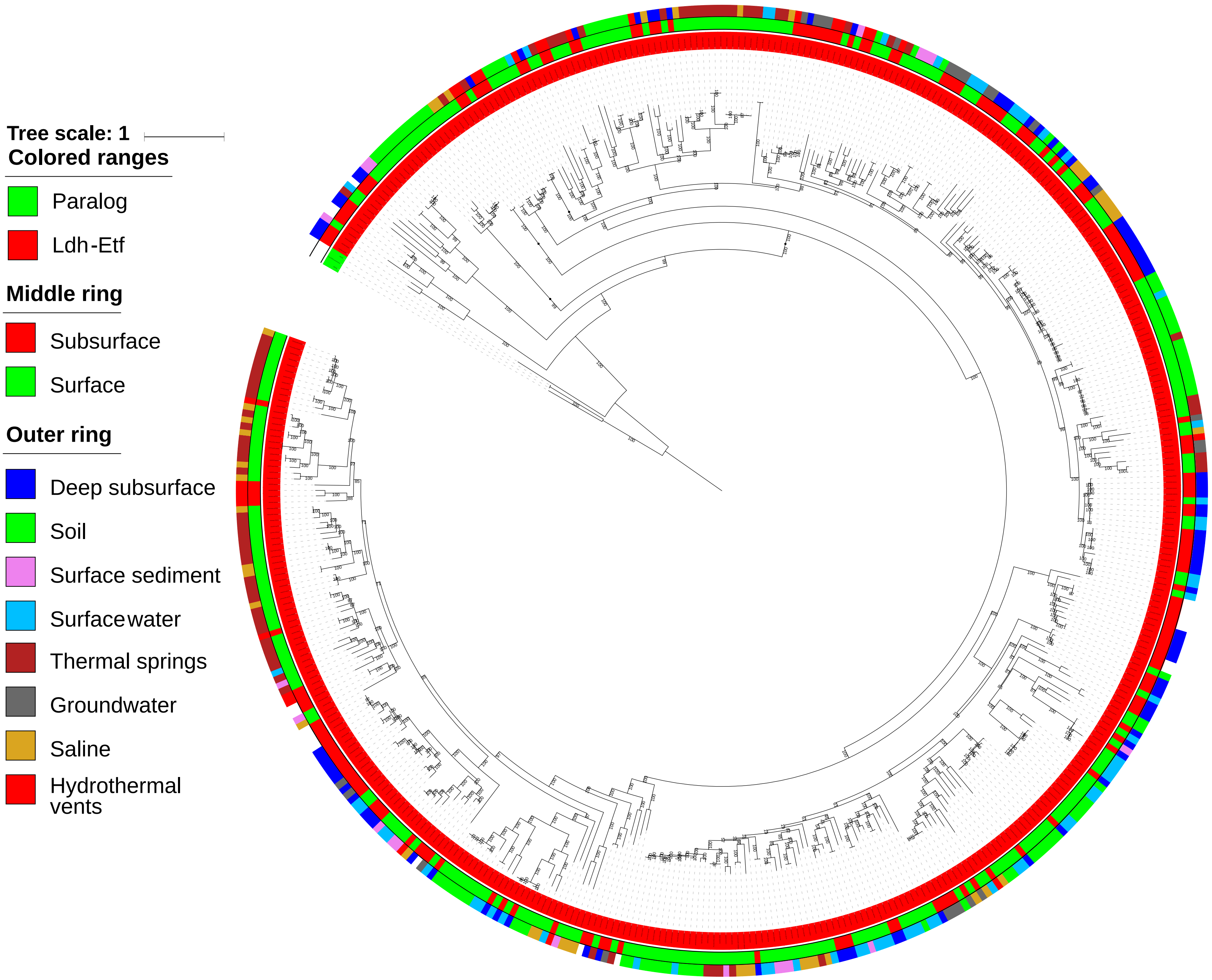

Supplementary Figure 29. Maximum-likelihood phylogenetic reconstruction of lactate dehydrogenase (Ldh) homologs that are predicted to form a complex with Bf Etf (i.e., Ldh) in metagenomic sequences. The phylogeny was rooted with the paralog alkyl dihyroxyacetone phosphate synthase from *Dictyostelium discoideum* (XP_637836) and *Trypanosoma brucei* (XP_845272). Sequence terminals are color coded, with the outer ring indicating the environment type where a given homolog was identified, the middle ring indicating whether the environment type that the homolog was identified in was classified as a surface or subsurface environment, and the inner ring indicating homology to Ldh (highlighted in red) or the paralog (alkyl dihyroxyacetone phosphate synthase highlighted in green). Protein homologs from metagenomes that lacked environmental classification were not colored in the middle and outer rings. Names of abbreviated protein complexes are provided in **Table 1.** Bootstrap values for each node are shown as a percentage (out of 1000 bootstrap replicates).

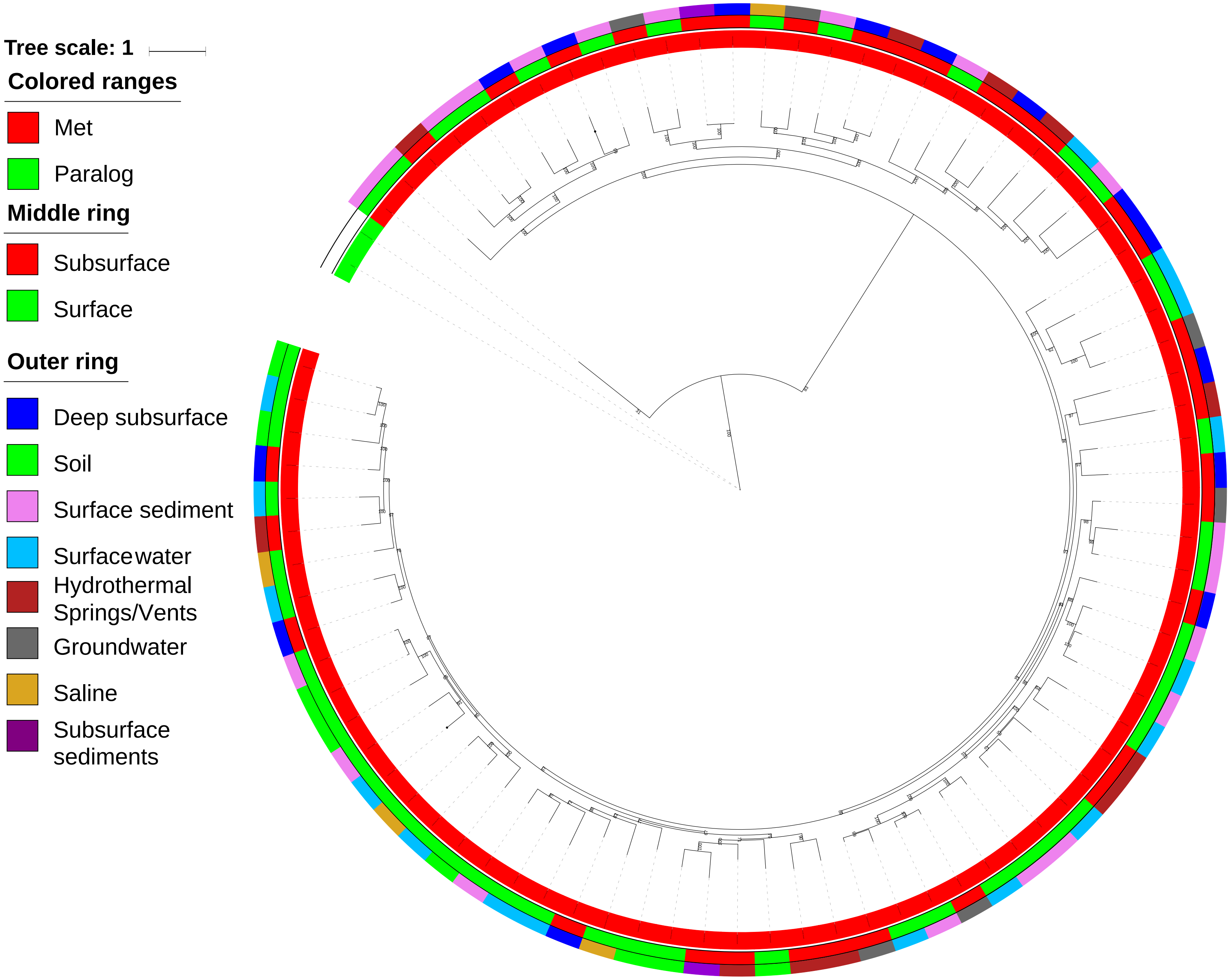

Supplementary Figure 30. Maximum-likelihood phylogenetic reconstruction of a concatenation of representative MetF homologs (that are predicted to form a complex with Bf Hdr) of each homolog 'bin' generated using a CD-HIT clustering approach applied to homologs identified in metagenomic sequences. All MetF homologs identified in metagenomes were first clustered into unique homolog 'bins' that contained closely related MetF homologs and the representative sequences of each unique 'bin' were extracted to reconstruct the phylogenetic tree of homologs of MetF. The tree was rooted with the paralog (terminals colored in green) methylenetetrahydrofolate reductase from *Escherichia coli* (WP_089580839) and *Shigella flexneri* (OUZ65700). Sequence terminals are color coded, with the outer ring indicating the environment type where a given homolog was identified, the middle ring indicating whether the environment type that the homolog was identified in was classified as a surface or subsurface environment, and the inner ring indicating homology to MetF-Hdr (highlighted in red) or the paralog (non-bifurcating methylenetetrahydrofolate reductase from highlighted in green). Protein homologs from metagenomes that lacked environmental classification were not colored in the middle and outer rings. Names of abbreviated protein complexes are provided in **Table 1.** Bootstrap values for each node are shown as a percentage (out of 1000 bootstrap replicates).
